# P4HA2 interacted with ATAD3A to modulate PINK1/parkin-dependent mitophagy and ^125^I brachytherapy sensitization in esophageal carcinoma

**DOI:** 10.1038/s41419-025-07864-x

**Published:** 2025-10-06

**Authors:** Xijuan Yao, Cheng Feng, Xing Huang, Songzhe Wu, Shuting Lu, Yang Gao, Tong Sun, Xiaxing Bai, Chenghui Li, Kaizhi Jia, Xue Han, Zhongkai Wang, Binda Chen, Xiaobin Wang, Jinhe Guo, Jian Lu

**Affiliations:** 1 Center of Interventional Radiology & Vascular Surgery, Department of Radiology, Cultivation and Construction Site of the State Key Laboratory of Intelligent Imaging and Interventional Medicine (Southeast University), Basic Medicine Research and Innovation Center of Ministry of Education, Zhongda Hospital, Medical School, Southeast University, 87 Dingjiaqiao Road, Nanjing, 210009 China; 2 Department of Pathology, Jiangsu Cancer Hospital& Jiangsu Institute of Cancer Research& Nanjing Medical University Affiliated Cancer Hospital, Nanjing, China; 3 School of Life Sciences and Technology, Advanced Institute for Life and Health, Southeast University, Nanjing, 210096 China; 4 Department of Radiology, Nanjing First Hospital, Nanjing Medical University, No. 68, Changle Road, Nanjing, 210006 China; 5 Department of Radiology, Jiangsu Cancer Hospital & Jiangsu Institute of Cancer Research & Affiliated Cancer Hospital of Nanjing Medical University, Nanjing, China; 6 Jaco Pharmaceuticals Co. Ltd., Ningbo, China; 7 Laboratory Animal Center, Southeast University, Nanjing, China

**Keywords:** Cancer therapeutic resistance, Prognostic markers, Targeted therapies

## Abstract

Interventional brachytherapy, such as iodine-125(^125^I), has improved the survival of obstructive late-stage esophageal cancer patients. However, most patients experience radioresistance after ^125^I brachytherapy. It is key to decipher the underlying mechanism of ^125^I radioresistance. In this study, we identified an endoplasmic reticulum-associated protein, P4HA2, which is upregulated and mediates resistance to ^125^I treatment. Mechanistically, P4HA2 enhances mitochondrial autophagy (mitophagy) via the PINK1/parkin pathway by binding to ATAD3A. Clinically, high expression of P4HA2 correlates with shorter overall survival and predicts poor prognosis with ^125^I brachytherapy. Moreover, the expression of P4HA2 is epigenetically increased by IGF2BP2 in an m^6^A-dependent manner. Notably, targeting P4HA2 with siRNA-based biocompatible nanomedicines significantly sensitizes ESCC to ^125^I brachytherapy. Collectively, our results show the molecular mechanism of mitophagy-mediated ^125^I radioresistance, which provides a potential therapeutic target and combinatorial strategy.

**Figure:**
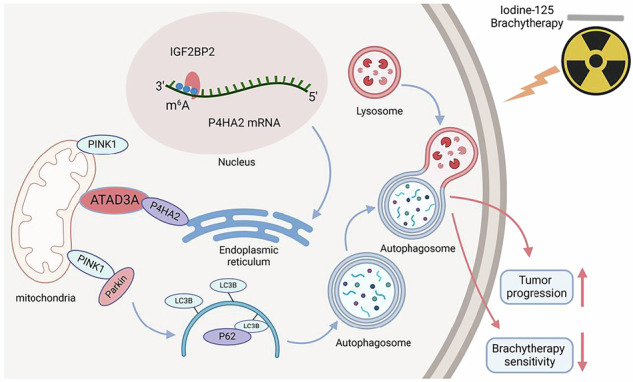
Schematic diagram of the role of P4HA2 in ^125^I brachytherapy for tumors

Schematic diagram of the role of P4HA2 in ^125^I brachytherapy for tumors

## Introduction

As one of the most common cancers, esophageal cancer is the sixth leading cause of cancer-related deaths, especially in Asia, where esophageal squamous cell carcinoma (ESCC) is more prevalent [1]. Notorious for its aggressive nature and difficulty of early diagnosis, the prognosis of esophageal cancer is poor overall, even worse for advanced cases, with a 5-year survival rate of less than 25% [2]. Our group introduced revolutionary internal brachytherapy for these patients by combining stent placement and ^125^I brachytherapy, which largely improved the prognosis and life quality of patients [3]. However, most of the patients receiving ^125^I-brachytherapy eventually experience radioresistance, which needs to be explored to understand the underlying mechanism and design more effective therapeutic strategies [4–7].

Mechanistically, brachytherapy (BT) has been found to induce reactive oxygen species (ROS)-mediated apoptosis and autophagy in tumor cells. Notably, significant endoplasmic reticulum (ER) stress in tumor cells is observed after BT radiation [8]. Inhibiting ER stress-mediated autophagy significantly enhances the anticancer efficacy of ^125^I brachytherapy (BT) [9–11]. However, the molecular mechanism by which ER stress-induced autophagy mediates the anticancer efficacy of BT remains elusive. Additionally, by eliminating damaged mitochondria, mitochondrial autophagy (mitophagy) aids some cancer cells in surviving post-radiotherapy, maintaining metabolic balance, and reducing the production of reactive oxygen species (ROS) [12, 13]. In most scenarios, modulating the activity of mitophagy can improve the efficacy of radiotherapy or help overcome resistance to radiotherapy [14–16]. However, the role of mitophagy in ^125^I brachytherapy is rarely reported.

Recently, P4HA2 (Prolyl-4-Hydroxylase Alpha Subunit 2) has been reported to play a pivotal role in oncology and is associated with poor prognosis [17]. P4HA2 stabilizes the HIF-1α protein, leading to a decrease in the levels of ROS in bladder cancer [18]. P4HA2 also promotes tumor proliferation, invasion, and migration [19–22], highlighting its potential as a cancer therapeutic target. However, the association of P4HA2 with continuous low-dose brachytherapy remains unreported. In this study, we revealed P4HA2-mediated ^125^I brachytherapy insensitivity, which enhances mitophagy by interacting with ATAD3A, a mitochondrial protein, as its critical effector mediating mitophagy. P4HA2 enhances the insensitivity of ^125^I brachytherapy by promoting mitophagy through modulation of the PINK1/parkin pathway. We further showed that biocompatible nanomedicines targeting P4HA2 augment the efficacy of ^125^I-brachytherapy, representing a genomics-guided targeted strategy to overcome radioresistance.

## Materials and methods

### Cell culture

Human ESCC cells (EC109 and TE-1) and ESCC cells (KYSE30 and KYSE150) were supplemented with 10% complete 1640 medium (Biochannel, China) and 1640/F12 medium (Biochannel, China), respectively Fetal bovine serum (FBS, Biochannel, China) and 1% penicillin/streptomycin (Gibco, USA). And HEK293T cells in complete Dulbecco modified Eagle medium (DMEM; Biochannel, China) supplemented with 10% fetal bovine serum (FBS, Biochannel, China) and 1% penicillin/streptomycin (Gibco, USA). All cell lines use short tandem repeat serial (STR) analysis for certification and use a month MycoAlert (Lonza, USA) to detect mycoplasma contamination.

### Patient samples

Between January 2017 and December 2018, a total of 185 esophageal squamous cell carcinoma (ESCC) tissue samples were collected from the Affiliated Cancer Hospital of Nanjing Medical University. These samples included both tumor and adjacent non-tumor areas and were processed into tissue microarrays. The inclusion criteria for patients were as follows: (1) pathologically diagnosed with primary esophageal squamous cell carcinoma; (2) no preoperative chemotherapy or radiotherapy; (3) adequately preserved paraffin-embedded tissue with clear structure; (4) complete medical records and follow-up information for over two years. For all patients, the pathological staging was conducted under the 8th edition of the American Joint Committee on Cancer (AJCC) Cancer Staging Manual, utilizing all available clinical and pathological data, including assessments of the tumor, nodes, and metastases. Additionally, detailed patient demographics and clinical-pathological characteristics, such as age, gender, tumor size, and histological grade, were collected. Detailed clinical data of the patients are provided in Table S1. Progression-free survival (PFS) was defined as the time interval from the date of the initiation of treatment until the disease progresses or death from any cause. Overall survival (OS) is defined as the time from the date of surgery to the date of death or the date of the last follow-up. All patients were divided into a high-expression group (*N* = 115) and a low-expression group (*N* = 70) based on P4HA2 expression. This study was approved by the ethics committee of the Affiliated Cancer Hospital of Nanjing Medical University. Samples were sourced from the biobank of Jiangsu Province Cancer Hospital (Jiangsu Province Cancer Research Institute and the Affiliated Cancer Hospital of Nanjing Medical University). All patients had signed informed consent forms for the donation of samples.

Twenty-four ESCC specimens were obtained from patients who underwent gastroscopy before brachytherapy at Zhongda Hospital, Southeast University. Detailed clinical data of the patients are provided in Table S2. Based on our previous study, 12 patients with an overall survival of less than 177 days were defined as ^125^I brachytherapy resistant (BT-RR). Conversely, 12 patients with an overall survival of more than 177 days were defined as brachytherapy sensitive (BT-RS). Overall survival (OS) is defined as the time from the date of ^125^I particle esophageal stent implantation to the date of death or the date of the last follow-up. For clinical tissue-related experiments, all studies were conducted in accordance with the international guidelines and ethical standards of the World Medical Association (Helsinki Declaration). This study received approval from the Ethics Committee of Zhongda Hospital affiliated with Southeast University.

### Cell and xenograft tumor model treated by brachytherapy

The ^125^I radiation model was performed according to the previous method [7], using polystyrene skin with a diameter of 35 mm to place one ^125^I seed in the center of a 60 mm cell culture, and the other eight ^125^I seeds were distributed equally across the circumference. 37 MBq radioactive particles from 6 mm, delivered a dose for an exposure time of 4 Gy, sustained over 90 h (calculated using the TPS system developed by Beijing University of Aeronautics and Astronautics) [23].

Animal experiments: 5-week-old male BALB/C nude mice were obtained from Vital River Laboratory (Beijing, China). All animal experimental methods were performed with the approval of the Ethics-by-Ethics Committee of Southeast University (20190225004). When the cell requirement was met, cells in the logarithmic growth phase were taken, digested, neutralized, and centrifuged to make a single-cell suspension in PBS, and the cell concentration was adjusted to 4 × 10^6^/ml. Nude mice with isoflurane anesthesia gas, take the right position, after disinfection, 75% alcohol in nude mice, left axillary subcutaneous injection of 0.1 ml single-cell suspension. Tumor volume was recorded every 3 days, and the formula for tumor volume was as follows: volume (*V*) = major diameter (*D*) × minor diameter (*W*)^2^ × 0.5. On the seventh day, the tumor-bearing mice were anesthetized with isoflurane. The mice were placed in the right position and sterilized with 75% alcohol. After preloading 29.6 MBq ^125^I seeds with an 18 G needle, the tumor was punctured 1 cm away from the edge of the tumor, and the tumor was punctured subcutaneously into the center. Advancing the needle core, inject ^125^I particles. Pull out the needle, local cotton ball oppression hemostasis. After surgery, patients were fed water freely. On the 15th day after surgery, all mice were anesthetized with isoflurane and then sacrificed by carbon dioxide inhalation. After the TPS system calculation, the average cumulative dose of 1 cm^3^ tumor was 33.09 Gy, 15 days after ^125^I seed implantation. Tumor specimens were collected and temporarily stored on ice, photographed, and weighed. The tumor was cut open, the position of ^125^I seeds was observed, and ^125^I seeds were recovered and placed in a lead tank.

### Tandem mass tag (TMT) labeling

A previous study demonstrated that ^125^I brachytherapy induced endoplasmic reticulum (ER) stress in ESCC cells, which was significantly elevated at a cumulative dose of 4 Gy (in EC109 cells) [10]. Additionally, it has been reported that irradiating tumor cells with 4 Gy and culturing them in vitro for four days can be used to identify the molecules necessary for tumor radioresistance [24]. Thus, EC109 cells were treated with or without a cumulative dose of 4 Gy of ^125^I BT, and total proteins were isolated using RIPA (Beyotime Biotechnology, China). Tandem Mass Tag (TMT) proteomics was submitted to Shanghai Biotree Biological Company for proteomics determination and analysis. The mean value between the ^125^I-treated group and control group (|log_2_ Fold change (FC)| > 1.2, *p* < 0.05) is supposed to be significantly upregulated.

### Plasmid transfection and siRNA knockdown

P4HA2 with a FLAG tag at the end, ATAD3A with an HA tag at the end, IGF2BP2 wild type, and mutant expression plasmids were cloned into the pLVX-IRES-Neo vector. P4HA2-FLAG and ATAD3A-HA expression plasmids were put into pcDNA3.1. Short hairpin RNA (shRNA for P4HA2, METTL14, and METTL3 was generated by cloning double-stranded oligonucleotides into vector pLVX-PURO. siRNAs for P4HA2, ATAD3A, and IGF2BP2 were purchased from Genepharma Inc. Plasmids and siRNAs were transfected with jetPRIME (Polyplus, France). By packaging with the plasmid vector pMD2.G, Lentivirus was prepared by co-transfecting specific plasmids with packaging vectors pMD2.G and psPAX2 into HEK293T cells at a ratio of 8:3:6. 72 h after transfection, the supernatant was collected, centrifuged at 4000*g* for 10 min, and the supernatant was used for infection.

### Cell invasion and migration assay

Migration and invasion assays were performed using transwell inserts (Corning, USA) with or without Matrigel (BD Biosciences, USA) according to the protocol. Migrating/invading cells were photographed and counted by light microscopy. A wound healing assay was used to evaluate cell migration ability. We seeded 5 × 10^5^ cells into six-well plates in triplicate. After cells had grown to approximately 90% confluence and had undergone specific treatments, a sterile 200 μl tube tip was used to scratch the center of the well. Cells were washed, cultured, and photographed with a light microscope at the indicated times.

### Cell proliferation assay

Cells from different treatment groups (3000 cells per well) were inoculated into 96-well plates six times. Then, after incubation with CCK-8(Vazyme, China) reagent at a specified time for 2 h, OD values (SpectraMax, China) were detected by an enzyme-labeled apparatus. To test the cloning ability of cells, 1000 cells were inoculated in triplicate into a six-well plate. Once the cells adhered to the surface, they were subjected to an radiation model where they accumulated a dose of 4 Gy. Post-radiation, the cells were removed from the radiation environment and subsequently cultured at 37 °C for 10–14 days to allow for colony formation. Colonies were stained with crystal violet and counted. Apoptosis was analyzed by flow cytometry (Agilent, China) using the Annexin V-FITC/PI Apoptosis Detection Kit (Vazyme, China). The cells were inoculated into 24-well plates (5 × 10^4^ cells/wells) in triplicate. After receiving a cumulative radiation dose of 4 Gy, the cells were incubated with 50 μM EdU (RiboBio, China) for 6 h. Subsequent dyeing and visualization are carried out according to the manufacturer’s instructions. The proportion of EdU-positive cells to total cells was calculated and analyzed in three random fields.

### Co-immunoprecipitation assay followed by mass spectrometry (MS)

Each group used three 10 cm dish cell plates, about 2 × 10^7^ cells. The cell medium was discarded, and the cells were rinsed with pre-cooled PBS solution 3 times. The cells were removed with a clean cell spatula and transferred to a 15 ml centrifuge tube. Cells were collected by centrifugation at 1000 rpm at 4 °C for 5 min. 1 ml of IP cracking solution (Beyotime Biotechnology, China) was added and placed on a 4 °C rotator for cracking for 30 min. The lysate was centrifuged in a centrifuge at 4 °C at 12,000*g* for 20 min, and the supernatant was collected. 10% of the volume of each group was absorbed as Input. Each group was added with 50 µl anti-FLAG M2 magnetic beads cleaned by IP lysate or corresponding antibody combined with Protein A/G (Thermo Fisher Scientific, USA), and the cell lysate and magnetic beads were placed on a rotator and incubated at 4 °C for 6 h. After the combination, the cell lysate was centrifuged at 4 °C and 500*g* for 5 min, and the supernatant was discarded. In total, 500 µl IP lysate was added, and the beads were transferred to a pre-cooled centrifuge tube. The magnetic beads were washed on a 4 °C rotator for 5 min, then centrifuged in a 4 °C centrifuge at 500*g* for 5 min, and finally, the supernatant was removed. Repeat the previous step eight times to drain the liquid from the beads. Boil an appropriate volume of 5× loading buffer. Alternatively, each group was diluted with 100 µl FALG peptide (Thermo Fisher Scientific, USA, 500 µg/µl) in 1× PBS and placed in a rotator at 4 °C for rotational elution for 30 min. Then centrifuge at 4 °C, 500*g* for 5 min and collect the supernatant as an elution sample. The elution sample was added into 5× loading buffer proportionally and boiled at 100 °C for 10 min for western blot analysis or frozen at −20 °C. Eluates were collected and visualized on 7.5% SDS-PAGE gels (Epizyme, China), followed by silver staining with a silver staining kit (Beyotime Biotechnology, China). Specific protein bands were retrieved and analyzed by LC–MS/MS.

### TnT® quick coupled transcription/translation systems

Follow the instructions to mix the P4HA2-FLAG (pcDNA3.1 vector) plasmid, ATAD3A-HA (pcDNA3.1 vector) plasmid, TnT® Rapid Premix (Promega, USA), methionine (1 mM), DNA template, and nuclease-free water to the appropriate volume. The reactants were incubated at 30 °C for 90 min. Then the protein was purified for the co-IP experiment.

### Quantitative PCR assay

Total RNA isolated using RNAiso Plus (Takara, Japan) was reverse transcribed using the RT SuperMix kit (Vazyme, China). qRT–PCR was performed using qRT–PCR SYBR Green Master Mix (Vazyme, China) and analyzed using the QuantStudio Flex real-time PCR System (Thermo Fisher Scientific, USA). PCR results, recorded as Ct values, were normalized against an internal control (GAPDH or 18S). Relative gene expression levels were analyzed using the 2^−^^ΔΔCT^ method. The primers used are listed in Table S4.

### RNA-sequence

Total RNA was extracted from cells using TRIzol Reagent (Thermo Fisher Scientific, China) and sent to Biotree Co., Ltd. (Shanghai, China) for high-throughput sequencing. Read the original count for DESeq2 differences in gene expression, GSEA analysis, etc. The complete dataset has been deposited in the Gene Expression Omnibus (GEO) database (GSE299511).

### Western blot analysis

ESCC and HEK293T cells were harvested and lysed in RIPA buffer (Beyotime Biotechnology, China) supplemented with protease inhibitors to obtain proteins according to the manufacturer’s instructions. For western blot, an equal amount of 10% or 12.5% (for LC3 I/II proteins) of protein was loaded onto an SDS-PAGE gel (Epizyme, China) per lane for separation and transferred to a polyvinylidene difluoride (PVDF) membrane. Membranes were blocked with 5% skim milk /BSA (BioFroxx, China) in 0.05% Tris-buffered saline/Tween (TBST) for 2 h and then at 4 °C with the corresponding antibodies (refer to Supplementary Table S5 for details) followed by horseradish peroxide-labeled secondary antibodies (1: 2000, Cell Signaling Technology, USA) for 2 h. Using a stationary protein root peroxidase substrate kit (Beyotime Biotechnology, China) for processing.

### Transmission electron microscopy detection

Exposed to continuous low-dose 4 Gy after radiation, the cell was fixed in the electron microscope fixed liquid (Servicebio, China), at 4 °C for the night. Then, the cells were maintained for 1 h in phosphate buffer containing 1% osmium tetroxide. After dehydration in a graded acetone series, cells were infiltrated and embedded in Epon. The embedded material was sectioned and stained with 3% uranyl acetate and lead citrate. We acquired images under an electron microscope (Japanese JEOL, 3000× or 6000× magnification).

### Mitophagy assay

To measure mitophagy flux, using Ad-GFP-LC3 (Hanbo Biological Technology Co., China) for the determination of immunofluorescence. Transfected and treated accordingly, the cells were incubated with Hoechst33342 (Yeasen, China) with a mitochondrial red fluorescent probe from MitoTracker Red CMXROS (Cell Signaling Technology, USA) for 30 min at 37 °C, washed three times with PBS, and cultured in Hank’s solution (Gibco, USA). Autophagy was observed by confocal microscopy (Olympus, Japan). Green fluorescence corresponds to changes in autophagic flux, and red fluorescence represents mitochondria. After the combination of red and green fluorescence images, yellow spots in the cell images are a symbol of mitophagy.

### Immunofluorescence (IF) and Immunohistochemistry (IHC)assay

Cells were seeded in confocal dishes, fixed with 4% paraformaldehyde (Biosharp, China), followed by three washes with PBST (PBS containing 0.1% Tween-20), and then incubated in immunofluorescence blocking solution (containing 3% BSA, 22.5 mg/ml glycine in PBS). They were incubated overnight with primary antibodies diluted according to Table S5. Subsequently, fluorescent secondary antibodies (Abcam, USA) were applied at room temperature for 1 h. Finally, cell nuclei were counterstained with DAPI containing an anti-fade agent (Beyotime Biotechnology, China).

All tissues were embedded in paraffin, sectioned to make slides, and all slides were placed in an incubator at 60 °C for 20 min, deparaffinized in xylene, and rehydrated in a gradient of ethanol. Will slide incubation with 3% hydrogen peroxide for 10 min, and then use 0.01 M citrate buffer (pH 6.0) for antigen repair for 30 min. After blocking with 5% BSA, the slides were incubated with primary antibodies at 4 °C overnight. Then, biotin-conjugated secondary antibodies were applied at room temperature for 1 h. IHC staining was visualized using a DAB (diaminobenzidine) reaction with hematoxylin counterstaining.

### RIP qPCR

In total, 2 × 10^7^ cells were washed twice with 10 ml ice-cold PBS and lysed in 400 μl of lysis Buffer containing 400 Uml^−1^ RNase inhibitor, and the supernatant was collected and centrifuged. Cell lysates were incubated with coated A/G magnetic beads (Millipore, USA) with antibodies to 2 μg of IGF2BP2 (Proteintech, China) for 6 h at 4 °C. A 10% supernatant of the RIP lysate was saved. The beads were washed six times in duplicate with 500 μl of precooled wash buffer (200 mM NaCl, 50 mM HEPES pH 7.6, 2 mM EDTA, 0.05%NP-40, 94 0.5 mM DTT, and 200 U ml^−1^ RNase inhibitor). 1 ml TRIzol was mixed and saved as IP samples, and previously extracted input samples were also mixed with 1 ml TRIzol. Total RNA was isolated by TRIzol and analyzed by qRT–PCR.

### m^6^A-RIP qPCR

Total RNA was extracted using TRIzol and treated with TURBO DNase (2 U/μl) (Invitrogen, AM2239) and then passed through RNA fragmentation reagent (Thermo, AM8740). Hundred micrograms of purified RNA was incubated with A/G magnetic beads (Millipore) coated with 3 μg of anti-m^6^A antibody (#202003, Synaptic Systems) for 6 h at 4 °C and dissolved in 500 μl NT2 buffer. Ten micrograms of purified RNA were saved as a starting sample. Then, with 500 μl icy NT2 buffer, washing beads were washed eight times and mixed with 1 ml TRIzol IP and saved as a sample. By TRIzol reagent, separation of total RNA was separated and analyzed through the qRT–PCR [25].

### Luciferase reporter assay

To assess the influence of m^6^A modification on the P4HA2 promoter, to isolate the pmirGLO-P4HA2-3′UTR-WT and pmirGLO-P4HA2-3′UTR mutants, or pGL3-P4HA2-5′UTR-WT and pGL3-P4HA2-5′UTR mutants, were transfected into IGF2BP2 KD cells for 48 h. A dual luciferase reporter gene assay system (Yeasen, China) was used to detect luciferase activity. Renal luciferase (R-Luc) is used to normalize F-Luc activity. mRNA abundance was determined by qRT–PCR of F-Luc and R-Luc, and the translation efficiency of P4HA2 was defined as the quotient of reported protein production (F-Luc/R-Luc) divided by mRNA abundance.

### mRNA stability

SiIGF2BP2 cells were treated with 5 μM actinomycin D (Act-D, #A9415, Sigma, USA) and incubated. Subsequently, the cells were harvested using TRIzol reagent at predetermined time points, following which RNA was isolated for qRT–PCR analysis.

### In vivo metastasis and in vivo biosafety assay measurement

In total, 4 × 10^6^ cells from the control and transfection groups were injected into the tail vein of nude mice to construct a mouse metastatic tumor model (*N* = 3 in each group). After 8 weeks, metastases were visualized by the IVIS Spectrum in vivo imaging system. Subsequently, all mice were euthanized. Lung tissues were harvested and further analyzed by H&E staining.

Nine healthy male Balb/c mice (5 weeks old) were randomly divided into three groups (*N* = 3 in each group). The groups received injections via the tail vein of either PBS, siLipo, or siLipo-FA. After 30 days, the mice were also euthanized, and the major organs (heart, liver, spleen, lung, and kidney) were collected for systemic pathological analysis and plasma biochemical analysis.

### Preparation and characterization of siLipo-FA

The FA-modified cationic liposome (FA-lipo) was prepared by the film dispersion-hydration method. Briefly, the mixture of DOTAP, cholesterol, DSPE-PEG2000, and DSPE-PEG2000-FA in the molecular ratio of 40:55:4.5:0.5 was dissolved in chloroform (5 mL). Then, the solvent was completely removed using rotary evaporators at 40 ± 2 °C until a thin film was obtained. The lipid film was hydrated with distilled water for 20 min and further sonicated for 5 min to form a preliminary liposome. The obtained liposome suspension was then filtered through a 0.22 μm polycarbonate membrane to obtain liposomes with a narrow size distribution. FA-lipo@SiRNA was prepared via electrostatic interaction using Lipo-FA and siRNA at a series of N/P ratios (molar ratio of DOTAP-nitrogen atoms to siRNA-phosphate) in RNase-free water. The zeta potential and average particle size of liposomes were determined by the dynamic light scattering (DLS) technique (Omec, China).

### Statistical analysis

Data are presented as the mean ± SEM of at least three independent biological replicates of experiments. Statistical differences were assessed by Student’s *t*-test or two-way ANOVA test. Kaplan–Meier method, Log-rank test, and Cox regression analysis were used to evaluate the prognostic value. All statistical analyses were performed with SPSS software, R software (version 3.6.0), or GraphPad Prism (version 8.0). *p < 0.05; **p < 0.01; *p* < 0.001, ns no significance.

## Results

### The upregulated level of P4HA2 in ESCC patients treated with ^125^I was positively associated with poor prognosis

**Fig. 1:**
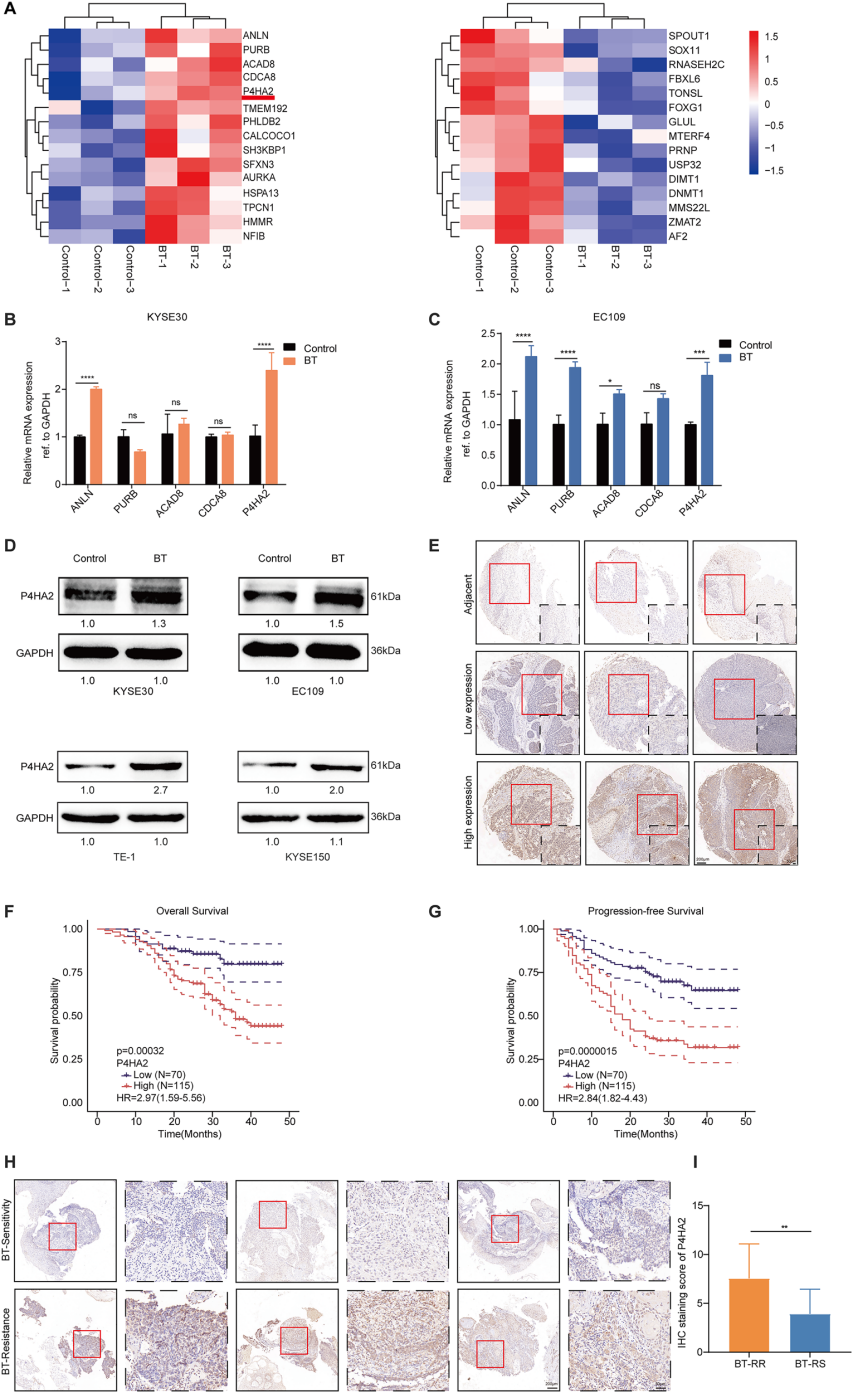
The upregulated level of P4HA2 in ESCC patients treated with ^125^I was positively associated with poor prognosis. **A** A Heatmap of the top 15 upregulated and 15 downregulated DEPs. Relative mRNA expression ratios of ANLN, PURB, ACAD8, CDCA8, and P4HA2 in the irradiated groups of KYSE30 (**B**) and EC109 cells (**C**). **D** Relative protein expression levels of P4HA2 in the irradiated group of KYSE30, EC109, TE-1, and KYSE150 cells. **E** Representative IHC images of P4HA2 in the histologic section of ESCC patients, and Kaplan–Meier curves for the overall survival (**F**) and progression-free survival (**G**) of patients with high P4HA2 expression (*N* = 115) and low P4HA2 expression (*N* = 70). Scale bars: 50 μm (20×), 200 μm (5×). **H** Representative IHC images of P4HA2 protein in tumor preoperative gastroscopic biopsy samples from patients with BT-RR and patients with BT-RS. Scale bars: 50 μm (20×), 200 μm (5×). **I** IHC score analysis in BT-RS (*N* = 12) and BT-RR (*N* = 12) patients. Data were presented with mean ± SD, ****p < 0.0001; ***p < 0.001; **p < 0.01 and *p < 0.05; ns not significant.

To investigate the molecular mechanisms of ^125^I brachytherapy for alleviating tumors, we performed Tandem Mass Tag (TMT) proteomics to analyze ESCC cells before and after receiving ^125^I, which was due to the limited high-quality preoperative biopsy samples from obstructive esophageal cancer patients. Compared to the untreated cell group, 67 dysregulated proteins (|log_2_ Fold change (FC)| > 1.2, *p* < 0.05) were identified in the ESCC cells treated with ^125^I, including 52 upregulated proteins and 15 downregulated proteins (Fig. 1A and S1A). Moreover, BT enhanced autophagy triggered by endoplasmic reticulum stress, leading to ESCC developing resistance to brachytherapy [10]. We further verified the mRNA expression of the top five upregulated proteins and found that the mRNA levels of ANLN and P4HA2 were increased in both KYSE30 and EC109 cells treated with ^125^I (Fig. 1B, C). Here, P4HA2, not ANLN, was located in the endoplasmic reticulum [26]. Therefore, we focused on the role of P4HA2 in ^125^I brachytherapy for esophageal squamous cell carcinoma (ESCC). Western blot assay also revealed an increase in the expression of P4HA2 at protein levels following brachytherapy in four ESCC cell lines, including KYSE30, EC109, TE-1, and KYSE150 cell lines (Fig. 1D and S1B). These results indicated the potential role of P4HA2 in the resistance of ESCC cells to ^125^I brachytherapy.

To explore the expression profile of P4HA2 in ESCC, we analyzed the Cancer Genome Atlas (TCGA) data using the GEPIA database [27] and found that the mRNA level of P4HA2 was significantly upregulated in ESCC specimens compared to adjacent normal tissues (Fig. S1C). Further, the same tissue microarray as in our previous study [28] was assessed to evaluate the clinical relevance of P4HA2 in 185 ESCC patients. The immunohistochemical (IHC) staining results showed that the P4HA2 expression was significantly elevated in tumor tissues from ESCC patients, compared to adjacent normal tissues (Fig. 1E and Table S1). The Kaplan–Meier curve analysis showed that the patients with high P4HA2 expression had shorter overall survival time and progression-free survival than those with low P4HA2 expression (Fig. 1F, G). Given that the overall survival of ^125^I radioactive stent therapy in patients with esophageal cancer was 177 days as a criterion [3], patients with an overall survival less than 177 days are classified as ^125^I brachytherapy-resistant (BT-RR), while those with an overall survival more than 177 days are considered to be ^125^I brachytherapy radiosensitive (BT-RS). Next, IHC staining (Fig. 1H) results revealed that the tumor tissues from BT-RR patients exhibited higher expression levels of P4HA2 than those from BT-RS patients (Fig. 1I and Table S2). Taken together, these results suggested that P4HA2 may act as an oncogene to promote ESCC progression and resistance to ^125^I brachytherapy.

### P4HA2 promotes ESCC metastasis in vitro and in vivo

**Fig. 2:**
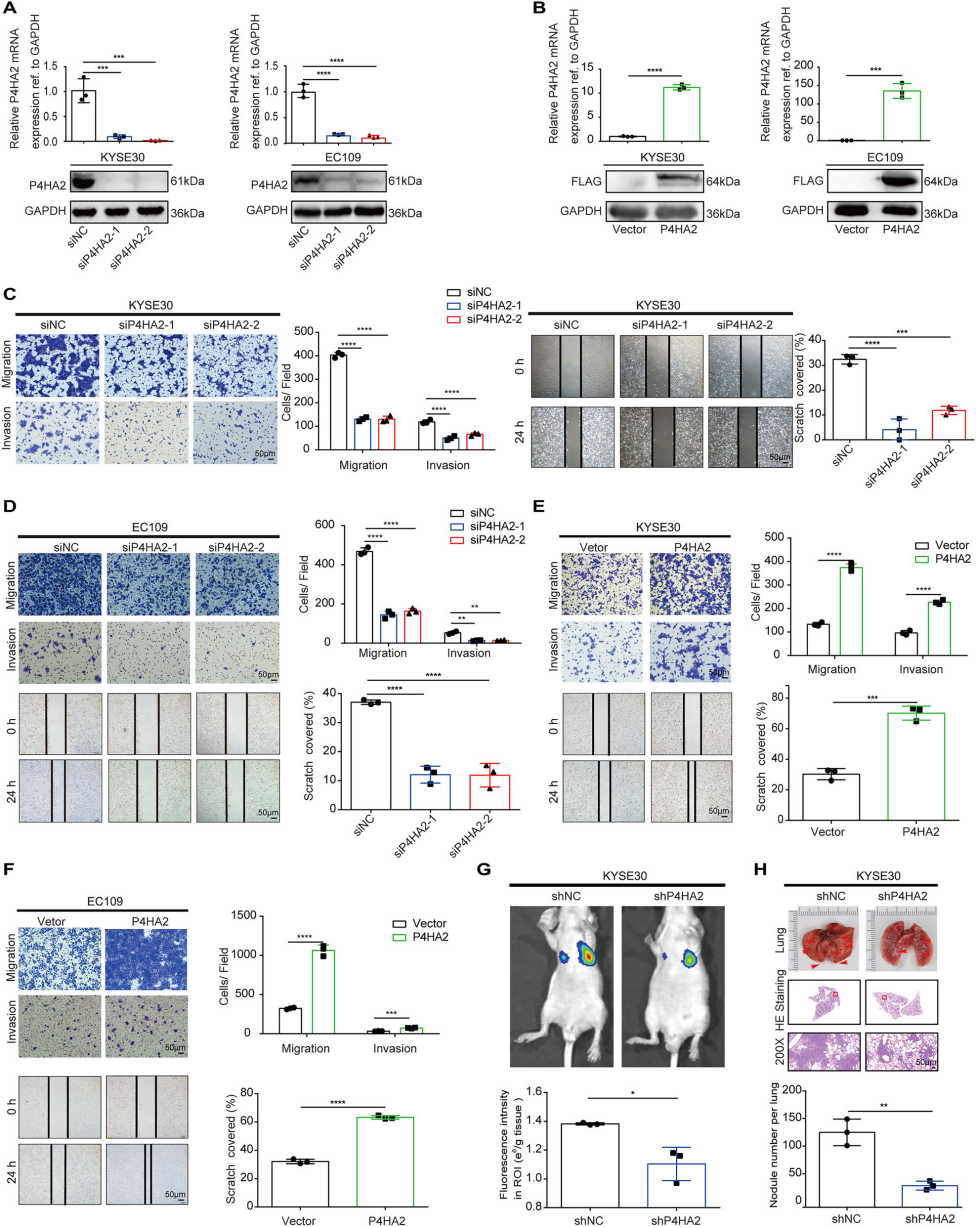
P4HA2 promotes ESCC metastasis in vitro and in vivo. **A** Knockdown and **B** Overexpression of P4HA2 were performed by qRT–PCR (upper panel) and western blot (WB) (lower panel) in KYSE30 and EC109 cells. The effects of P4HA2 loss (**C**, **D**) or gain (**E**, **F**) of function on in-cell migration, invasion, and wound healing assays. Upper, transwell assay, scale bar: 50 μm; lower, wound healing assay. Scale bar: 50 μm. **G** Representative bioluminescent images of lungs for each experimental group (*N* = 3). **H** Representative images of lung and H&E stains of lung metastases. Data were presented with mean ± SD, *p < 0.05; **p < 0.01; ***p < 0.001; ****p < 0.0001.

Previous studies linked high P4HA2 expression in prostate and cervical cancers to tumor metastasis [22, 29]. To further explore the biological function of P4HA2 in ESCC, the short interfering RNA (siRNA) targeting P4HA2 and lentiviruses expressing P4HA2 were transfected into KYSE30 and EC109 cells, respectively. The effectiveness of transfection was verified using qRT–PCR and western blot (Fig. 2A, B). In vitro, P4HA2 knockdown (KD) inhibited the migratory and invasive abilities of KYSE30 and EC109 cell lines (Fig. 2C, D). In contrast, overexpression (OE) of P4HA2 dramatically accelerated the cell migratory and invasive abilities of two ESCC cell lines (Fig. 2E, F). To detect the effect of P4HA2 on ESCC metastasis in vivo, we designed two short hairpin RNA (shRNA) lentiviruses to silence the expression of P4HA2 in KYSE30 cells with a high-migration capacity (Fig. S2A) and injected these cells into nude mice. As shown by the in vivo imaging system, the luciferase fluorescence signal of the lung region of nude mice indicated that P4HA2 KD reduced the ESCC metastasis (Fig. 2G), which was further confirmed by H&E staining results (Fig. 2H). Altogether, these data suggested that P4HA2 was able to enhance metastasis and invasion in ESCC.

### P4HA2 inhibits the sensitivity of ESCC to ^125^I brachytherapy

**Fig. 3:**
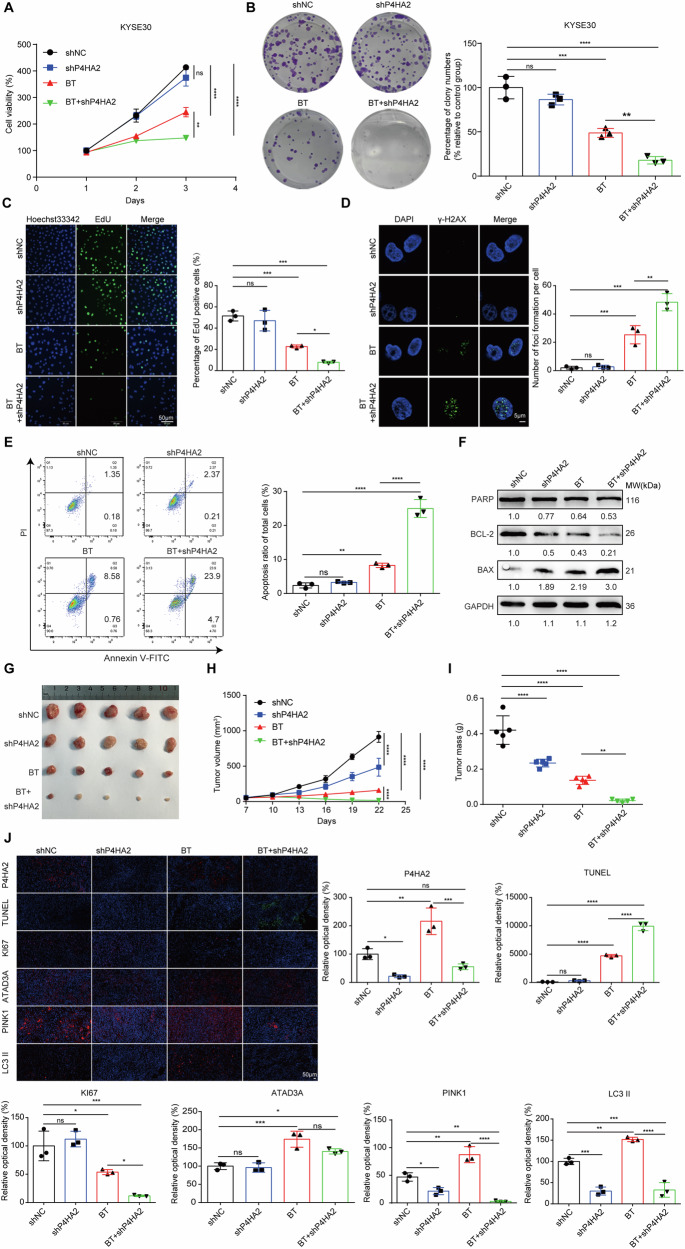
P4HA2 enhances the resistance of ESCC to ^125^I brachytherapy. **A** CCK-8 assay and **B** colony formation assays were performed to evaluate the cell proliferation ability of P4HA2-knockdown cells after radiation exposure in KYSE30 cells. **C** KYSE30 cells treated with or without radiation were stained with EdU (green) fluorescence. Scale bar: 50 μm. **D** IF was used to examine γ-H2AX foci in KYSE30 cells that were treated with radiation (4 Gy). Scale bar: 5 μm. **E** The effect of P4HA2 knockdown on apoptosis in the presence or absence of brachytherapy was determined by flow cytometry. **F** The protein levels of PARP, BCL-2, and BAX in KYSE30 cells with P4HA2 KD were assessed with or without radiation exposure. **G** Representative images of xenografts from the indicated treatment groups. **H** Tumor growth of nude mice was measured every three days (*N* = 5). **I** Tumor weight was measured at the end of the experiment (*N* = 5). **J** Representative images and quantitative fluorescence of IF staining for P4HA2, TUNEL, KI67, PINK1, and LC3 II in xenograft tumor tissues. Scale bar: 50 µm. Data were presented with mean ± SD, ****p < 0.0001; ***p < 0.001; **p < 0.01 and *p < 0.05; ns not significant.

Next, we explored whether P4HA2 in ESCC could alter the response to ^125^I brachytherapy and performed CCK-8, colony formation, and EdU assay. ^125^I brachytherapy treatment resulted in a pronounced inhibition of tumor cell proliferation and colony formation in P4HA2 KD cells compared with the control group. (Fig. 3A–C and S2B–D). In contrast, P4HA2 OE increased the growth ability of cells against radiation in both KYSE30 and EC109 cells (Fig. S3A–D). However, the ability of cell proliferation and colony formation in P4HA2 KD and OE cells compared with the control cells was similar. DNA damage was a primary and intrinsic factor and the most crucial operator in the response to radiation exposure, which may trigger an apoptotic signaling cascade that forces cells into programmed cell death [30]. Thus, we evaluated the effect of P4HA2 on DNA damage and cell apoptosis against radiation. Immunofluorescence (IF) of a DNA damage sensor γ-H2AX and flow cytometry results showed that P4HA2 KD (Fig. 3D and S2E) and OE (Fig. S3G) exerted little effect on the nuclear DNA damage and apoptosis in both KYSE30 and EC109 cells. However, upon radiation exposure, P4HA2 KD remarkably increased the number of γ-H2AX foci (Fig. 3D and S2E), resulting in the increase of apoptosis rates in both KYSE30 and EC109 cells (Fig. 3E and S2F). Conversely, P4HA2 OE elicited a significant reduction in the number of γ-H2AX foci (Fig. S3G) and in the apoptosis rates on KYSE30 and EC109 cells against radiation (Fig. S3E). Clearly, immunoblotting of apoptosis-related proteins, including Bax, Bcl-2, and PARP, also supported these findings (Fig. 3F, S2G, and S3F). Autophagy avoids tumor cell death, which results in the treatment tolerance of brachytherapy [31]. Moreover, inhibition of endoplasmic reticulum stress proteins reduced protective autophagy in ESCC cells [10]; thus, we investigated whether P4HA2 enhances the resistance of ESCC cells to brachytherapy through autophagy. Immunoblotting showed that P4HA2 KD reversed the increased expression of SQSTM1/P62 and the decreased expression of Beclin-1 in ESCC cells after exposure to low-dose radiation (Fig. S4A). Furthermore, we performed transcriptome sequencing and Gene Set Enrichment Analysis (GSEA). The results also showed that, compared to the control group, the P4HA2 KD with BT group could downregulate the autophagy pathway (Fig. S4B). These results suggested that P4HA2 enhanced the resistance of ESCC cells to brachytherapy through inducing autophagy.

To further investigate the role of P4HA2 in mediating the response of ESCC to ^125^I brachytherapy in vivo. P4HA2 KD and control KYSE30 cells were subcutaneously injected into nude mice. In vivo xenograft experiments showed that P4HA2 KD increased ESCC sensitivity to ^125^I brachytherapy, as evidenced by the decreased volume and mass of tumors from mice treated with the radiation. (Fig. 3G–I). Moreover, IF analysis on mouse tumors demonstrated in tumors with P4HA2 KD, KI67 distribution was significantly lower than that in the control group, and the lowest positive rate was observed in the synergy treatment group. Subsequently, TUNEL staining confirmed that P4HA2 regulated ESCC cell apoptosis in vivo, particularly in the combined treatment group. Post-brachytherapy, a slight increase in P4HA2 protein was observed in the shP4HA2 group (Fig. 3J). Altogether, these studies revealed that P4HA2 enhanced the radioresistance of ESCC during brachytherapy.

### ATAD3A interacts with P4HA2 in mitochondria and the endoplasmic reticulum

**Fig. 4:**
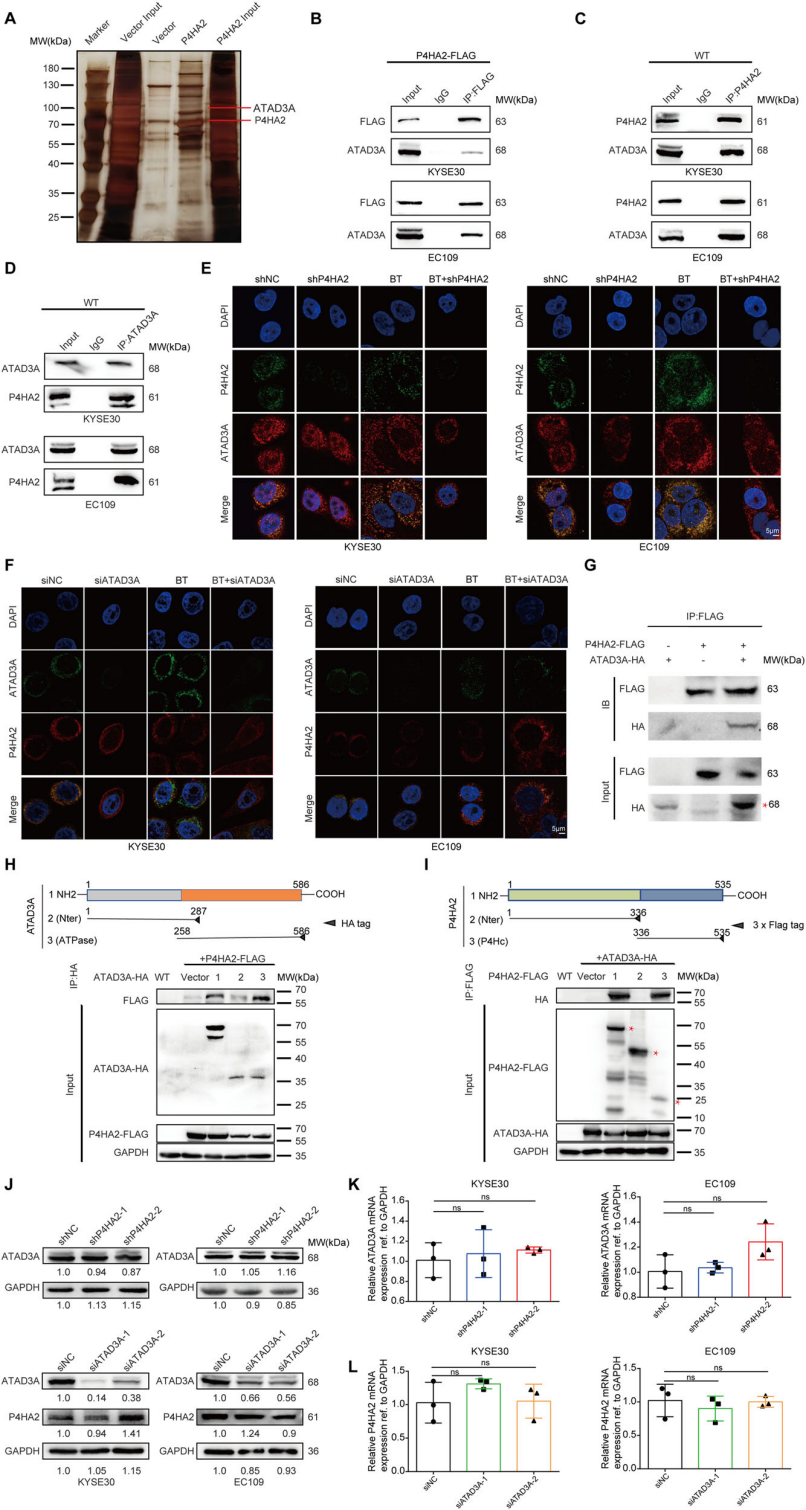
ATAD3A interacts with P4HA2 in mitochondria and the endoplasmic reticulum. **A** Silver-stained protein bands were visualized by IP with anti-FLAG microbeads using total protein extracts from EC109 cells expressing P4HA2-FLAG or vectors. **B** Lysates from P4HA2-FLAG overexpressing KYSE30/EC109 cells were used for the co-immunoprecipitation (co-IP) assay of P4HA2 and ATAD3A proteins. **C**, **D** The lysates of endogenous ATAD3A and P4HA2 interactions were detected by co-IP in KYSE30 and EC109 cells. **E**, **F** Confocal images showing the co-localization of ATAD3A and P4HA2 in ESCC cells treated with different groups. Scale bar: 5 μm. **G** Through in vitro transcription-translation systems. the interaction between P4HA2 and ATAD3A. Schematic of various ATAD3A-HA truncated (**H**) or P4HA2-FLAG truncated (**I**) and western blot analysis of co-IP of HEK293T cell lysates overexpressing P4HA2-FLAG and/or various ATAD3A-HA truncated (**H**) or ATAD3A-HA and/or various P4HA2-FLAG truncated (**I**). WT wild type, 1 full length, 2–3 as different truncated plasmids. **J** The protein levels of ATAD3A and P4HA2 in ESCC cells were assessed by WB. qRT–PCR assay for the expression of ATAD3A (**K**) and P4HA2 (**L**) genes in cells from different treatment groups. Data were presented with mean ± SD, ****p < 0.0001; ***p < 0.001; **p < 0.01 and *p < 0.05; ns not significant.

To understand the mechanism of P4HA2-mediated resistance of ESCC to ^125^I brachytherapy, FLAG-tagged P4HA2 (P4HA2-FLAG) was stably expressed in EC109 cells. Cell extracts were prepared and purified by anti-FLAG affinity microspheres, and the interaction partners of P4HA2 were identified by co-immunoprecipitation (co-IP) coupled with liquid chromatography-tandem mass spectrometry (LC-MS/MS) (Fig. 4A and Table S3). We focused on the ATPase family AAA domain-containing 3 A (ATAD3A) protein, which is enriched among all identified proteins and is critical for the regulation of mitophagy [32–34]. To further verify the interaction between P4HA2 and ATAD3A, using P4HA2-FLAG-expressed cells and wild type from KYSE30 and EC109, we performed co-IP and immunoblotting (IB) and confirmed its endogenous association between P4HA2 and ATAD3A (Fig. 4B–D). Moreover, immunofluorescence showed that P4HA2 and ATAD3A were co-localized in the cytoplasm, and this colocalization was increased after radiation (Fig. 4E, F). Further, through in vitro transcription-translation systems, we also found the direct binding between P4HA2 and ATAD3A proteins (Fig. 4G). To further determine the precise interaction of the two proteins, the HA or FLAG-tagged truncated plasmids of ATAD3A or P4HA2 were transfected into HEK293T cells. Immunoblot assays showed that the interaction between ATAD3A and P4HA2 required the amino acids 258-586 (ATPase domain) of ATAD3A (Fig. 4H). Similarly, the truncated mapping of P4HA2 showed that the binding with ATAD3A required the serialization of amino acids spanning fragments 336-535 (P4Hc domain) (Fig. 4I). These results supported that P4HA2 binds to ATAD3A.

To further characterize the molecular consequences of the P4HA2 and ATAD3A interaction, P4HA2 or ATAD3A KD reduced the co-localization between P4HA2 and ATAD3A but did not significantly change the fluorescence intensity of ATAD3A or P4HA2 (Fig. 4E, F). Consistently, qPCR and IB assays also confirmed that P4HA2 and ATAD3A failed to alter each other’s expression level (Fig. 4J–L). The molecular docking prediction results revealed a specific interaction interface between the P4HC domain of P4HA2 (depicted in blue) and the ATPase domain of ATAD3A (depicted in orange) (Fig. S4E). The predicted interaction residues were located within the P4HC and ATPase domains, which were experimentally confirmed to interact through co-immunoprecipitation (co-IP) assays. Further analysis demonstrated that P4HA2 interacts with ATAD3A through an arene-H bond, involving key residues such as Ser531, Arg321, Glu533, and Phe322. These residues form an intricate network of interactions, including arene-H bonds, indicating a stable and specific binding mode between P4HA2 and ATAD3A (Fig. S4E). In addition, immunofluorescence in mouse tumor tissues confirmed that after P4HA2 knockdown, ATAD3A expression levels remained unchanged. Even after iodine-125 irradiation, ATAD3A expression levels were maintained, but the BT combined with P4HA2 KD group still showed relatively low levels (Fig. 3J). These findings suggested P4HA2 and ATAD3A may act as partners to play roles in ESCC.

### P4HA2 and ATAD3A as complexes to regulate mitophagy via the PINK1/parkin pathway to suppress radiotherapy sensitivity

It has been reported that the ATAD3A forms complexes with numerous proteins in the endoplasmic reticulum (ER) to regulate mitophagy [35]. ATAD3A inhibits PINK1-dependent mitophagy to maintain cellular homeostasis [32, 34, 36, 37].

**Fig. 5:**
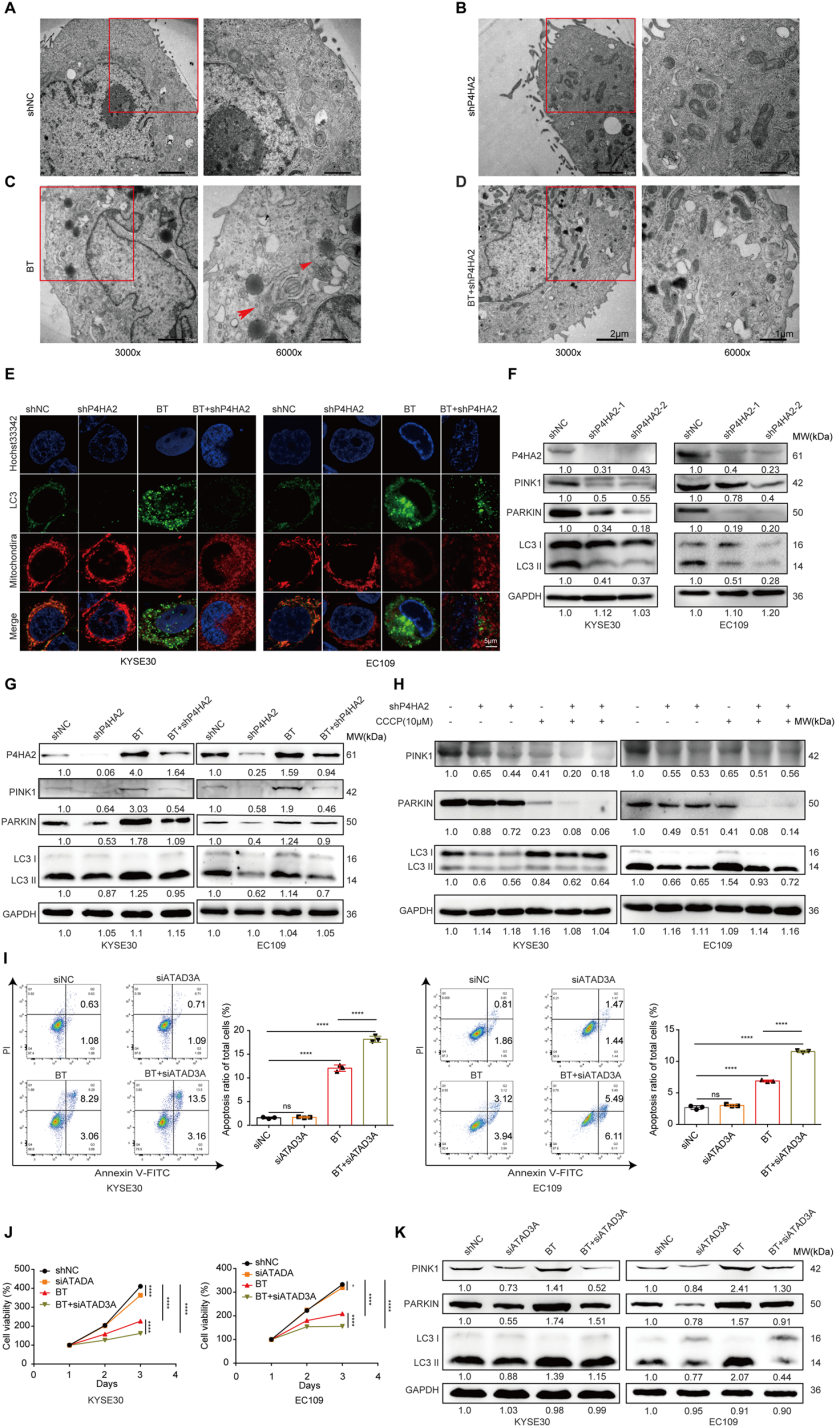
P4HA2 and ATAD3A as complexes to regulate mitophagy via the PINK1/parkin pathway to suppress radiotherapy sensitivity. **A** Normal morphology of mitochondria and organelles in KYSE30 cells was observed under transmission electron microscopy. **B** Mild swelling of some mitochondria was observed in the P4HA2 KD group. **C** Mitochondrial swelling, lysosomal accumulation, and mitophagy (indicated by arrows) were observed in cells after radiation alone. **D** Mitochondrial damage remained severe in cells after radiation combined with P4HA2 KD, but mitophagy was not observed. **E** Representative fluorescent images of LC3 (green), nuclei (blue), and Mitochondria Tracker (red) of cells transfected with GFP-LC3 were obtained from different treatment groups (60×). Scale bar: 5 μm. Representative graphs show P4HA2, PINK1, parkin, and LC3 II protein levels after silencing P4HA2 (**F**) and combining it with ^125^I brachytherapy (**G**). **H** PINK1, parkin, and LC3 II protein levels were measured after two hours of treatment with 10 μM CCCP in control and P4HA2 KD groups. **I** The effect of ATAD3A knockdown on apoptosis in the presence or absence of brachytherapy was determined by flow cytometry. **J** CCK-8 assay was performed to evaluate the cell proliferation ability of ATAD3A-knockdown cells after radiation exposure, both in KYSE30 cells and EC109 cells. **K** PINK1, Parkin, and LC3 II protein levels in ESCC cells with ATAD3A knockdown were assessed with or without radiation exposure. Data were presented with mean ± SD, ****p < 0.0001; ***p < 0.001; **p < 0.01 and *p < 0.05; ns not significant.

To further investigate whether P4HA2 regulates BT-sensitive cells through mitophagy, we examined cell substructures using transmission electron microscopy. Results showed that compared to control cells (Fig. 5A), there were no significant changes in mitochondria morphology in the P4HA2 knockdown group (Fig. 5B). However, upon radiation, there was a substantial increase in mitochondrial formation accompanied by evident damage and observation of lysosomes and mitophagosomes (Fig. 5C). Next, varying degrees of mitochondrial damage were observed ranging from moderate to severe. However, there was almost no evidence of autophagic lysosomes or mitophagy in P4HA2 KD under radiation (Fig. 5D and S4F), indicating an inhibition of autophagic flux. Moreover, the levels of fluorescence colocalization (yellow fluorescence) of autophagosomes (green fluorescence) and mitochondria (red fluorescence) were also assessed. Compared to the control group, the yellow fluorescence intensity significantly decreased in the P4HA2 KD group. Even under radiation exposure, P4HA2 KD markedly suppressed the yellow fluorescence intensity, indicating that P4HA2 could assist ESCC cells in resisting BT attacks through mitophagy (Fig. 5E). Changes in the levels of PINK1/parkin-dependent mitophagy-related proteins (including PINK1, parkin, LC3II) also demonstrated that P4HA2 regulates PINK1/parkin-dependent mitophagy (Fig. 5F). In addition, despite the elevation in PINK1/parkin and LC3 II levels after radiation exposure, their expression in the P4HA2 KD group continued to decrease (Fig. 5G). These findings demonstrated that P4HA2 enhances the radiation tolerance of ESCC cells by regulating mitochondria through the PINK1/parkin pathway.

Given that mitophagy is a dynamic physiological process, we further clarified the facilitation role of P4HA2 in mitophagy. Carbonyl cyanide m-chlorophenyl hydrazone (CCCP), a common agonist of mitophagy, was used to treat ESCC cells for 2 h, activating cellular mitophagy [38]. After CCCP treatment, P4HA2 KD continued to inhibit the production of LC3 II. Simultaneously, the expression of PINK1 and parkin was also suppressed (Fig. 5H). To evaluate the impact of P4HA2 on mitochondrial autophagy in iodine-125 brachytherapy in vivo, we analyzed the expression levels of PINK1 and LC3 II proteins through immunofluorescence. The results confirmed that when P4HA2 expression was reduced, the expression levels of PINK1 and LC3 II proteins were also downregulated. Although iodine-125 brachytherapy increased mitochondrial autophagy levels, the mitochondrial autophagy levels remained low in the P4HA2-KD combined with ^125^I BT group (Fig. 3J).

To further investigate whether ATAD3A regulates BT sensitivity in cells through mitochondrial autophagy, the effect of ATAD3A on the radiosensitization of ESCC cells was evaluated. Compared to controls, ATAD3A KD under brachytherapy increased apoptosis (Fig. 5I) and inhibited the proliferation (Fig. 5J) of ESCC cells. ATAD3A acts by regulating mitochondrial autophagy through modulation of the PINK1 pathway, and the effect on mitochondrial autophagy was subsequently assessed after irradiation of ESCC cells with ^125^I. The levels of PINK1/parkin-dependent mitochondrial autophagy-associated proteins (including PINK1, parkin, and LC3II) also showed that downregulation of ATAD3A increased radiosensitization (Fig. 5K) by inhibiting PINK1/parkin-dependent mitochondrial autophagy, as compared with irradiation alone. These results collectively revealed that P4HA2 promotes cancer cell mitophagy through the PINK1/parkin pathway, enhancing resistance to BT treatment.

### IGF2BP2 increases the expression of P4HA2 in an m^6^A-dependent manner

**Fig. 6:**
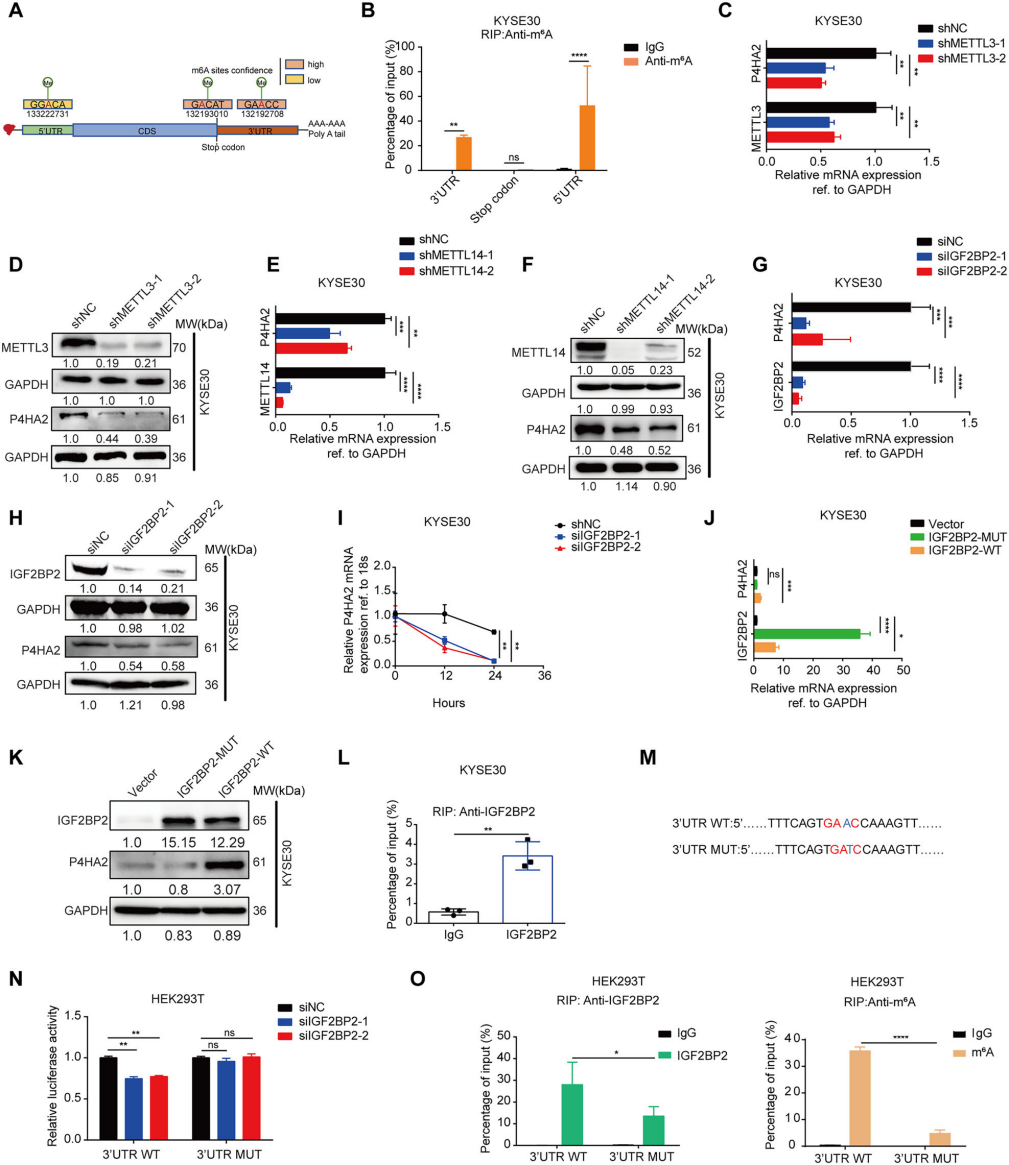
IGF2BP2 increases the expression of P4HA2 in an m^6^A-dependent manner. **A** Schematic representation of the predicted m^6^A modification sites of P4HA2 by the RMvar database and m^6^A RIP qRT–PCR showing enrichment of m^6^A modification at different positions in KYSE30 (**B**). qRT–PCR and western blot were used to analyze the expression levels of both the gene and protein of P4HA2 in shMETTL3 (**C**, **D**) and shMETTL14 (**E**, **F**) cells. qRT–PCR (**G**) and western blot (**H**) analysis of P4HA2 in IGF2BP2 KD in the KYSE30 cell. **I** Half-life of P4HA2 after treatment with 5 μM actinomycin D for the indicated times in the KYSE30 cell line with IGF2BP2 KD. qRT–PCR (**J**) and immunoblot (IB) (**K**) analysis of P4HA2 expression in KYSE30 cells transfected with IGF2BP2 wild-type or mutant. **L** RIP showed enrichment of IGF2BP2 in P4HA2 within the KYSE30 cell line. Schematic representation of the luciferase reporter mutation sites (**M**) and dual luciferase assays (**N**) shows the luciferase activity of these constructs. **O** RIP qRT–PCR detection of P4HA2 3′UTR WT and MUT in the enrichment of IGF2BP2 (middle) and m^6^A (right) in HEK293T cells. Data was presented with mean ± SD, ****p < 0.0001; ***p < 0.001; **p < 0.01 and *p < 0.05; ns not significant.

In addition to the previously reported transcriptional regulation of P4HA2 by NF-κB [21] and HIF-1 [39], we further investigated the potential post-transcriptional modifications of P4HA2. Among various RNA modifications, N^6^-methyladenosine (m^6^A), the most prevalent mRNA modification, has emerged as a crucial regulator of gene expression and diverse biological processes [40–42]. To determine whether m^6^A modifies the P4HA2 and regulates its expression, we predicted the potential m^6^A modification sites of P4HA2 using the RMvar database [43]. Two high confidence m^6^A sites in the 3′ untranslated region (3′UTR) and the stop codon, and a low confidence m^6^A sites in the 5′UTR were identified (Fig. 6A). We therefore performed m^6^A-RIP qPCR and confirmed that both the 5′UTR and 3′UTR of P4HA2 have m^6^A-modified in KYSE30 and EC109 cells (Fig. 6B and S5A), compared to a negative control IgG. To examine the effect of m^6^A modification on the P4HA2 expression, we KD methyltransferase-like protein 3 (METTL3) and methyltransferase-like protein 14 (METTL14), respectively, two key components of the large m^6^A methyltransferase complex in mammalian [44]. qRT–PCR and IB assays showed that the mRNA and protein levels of P4HA2 were reduced after METTL3 or METTL14 KD in KYSE30 (Fig. 6C–F) and EC109 cells (Fig. S5B–E). Together, these results confirmed that m^6^A modifies the *P4HA2* and increases its expression in ESCC.

To identify which m^6^A readers mediate the regulation of METTL3/METTL14 on P4HA2 expression, we performed an expression correlation analysis between P4HA2 and eight major m^6^A reader regulators in TCGA Pan-Cancer. Spearman correlation analysis revealed that IGF2BP2 was significantly positively correlated with P4HA2 in ESCC (Fig. S5F). To determine the effect of IGF2BP2 on the P4HA2 gene, we transfected siRNAs targeting IGF2BP2 and found that the mRNA and protein levels of P4HA2 were decreased in both KYSE30 (Fig. 6G, H) and EC109 (Fig. S5G, H) cells. Moreover, IGF2BP2 KD remarkably inhibited the half-life of the P4HA2 mRNA in both KYSE30 (Fig. 6I) and EC109 (Fig. S5I) cells. Next, wild-type IGF2BP2 (IGF2BP2-WT) and flag-tagged mutant IGF2BP2 (IGF2BP2-Mut) with mutations of GxxG to GEEG in the KH3–4 domains to abolish its m^6^A recognition and binding [45] were transfected into both KYSE30 (Fig. 6J, K) and EC109 (Fig. S5J, K) cells. IGF2BP2-WT OE, but not IGF2BP2-Mut, increases the mRNA and protein levels of P4HA2. Further, IGF2BP2 RIP–qPCR also showed the enrichment of IGF2BP2 on the *P4HA2* transcript in KYSE30 (Fig. 6L) and EC109 (Fig. S5L) cells, suggesting that IGF2BP2 bound and stabilized the *P4HA2* in an m^6^A-dependent manner. To further confirm the exact regulation of m^6^A modification and IGF2BP2 on *P4HA2*, we first constructed luciferase reporters for the 5′UTR (Fig. 6M) and 3′UTR (Fig. S5M) in *P4HA2* that contain the wild-type or mutated RRACH m^6^A motif [46]. Luciferase reporter assays showed that IGFBP2 KD reduced the luciferase activity of the 3′UTR wild-type reporter but not the mutant reporter (Fig. 6N). However, IGFBP2 KD did not alter the luciferase activity of the wild-type and mutant 5′UTR reporter (Fig. S5N), suggesting that the m^6^A site “GAAC” in the 3′UTR of *P4HA2* is required for the regulation of IGF2BP2 on *P4HA2* expression. Furthermore, IGF2BP2 and m^6^A RIP–qPCR assays showed that IGF2BP2 and m^6^A were more effectively bound to the WT 3′UTR of *P4HA2* compared with the mutated 3′UTR in HEK293T cells (Fig. 6O). Further, RNA-EMSA results revealed that IGF2BP2 bound to the 3′UTR region of *P4HA2*. However, when the m^6^A site in the 3′UTR of *P4HA2* was mutated, IGF2BP2 was unable to bind to the *P4HA2* 3′UTR region (Fig. S5O). This finding provided direct evidence that the m^6^A modification at the 3′UTR of *P4HA2* is essential for IGF2BP2 binding and stabilization of P4HA2 mRNA. Taken together, these data suggested that IGF2BP2 stabilizes P4HA2 and increases its expression in an m^6^A-dependent manner.

### Folate-targeted cationic liposome carrying siRNA ameliorates the brachytherapy therapeutic effect

**Fig. 7:**
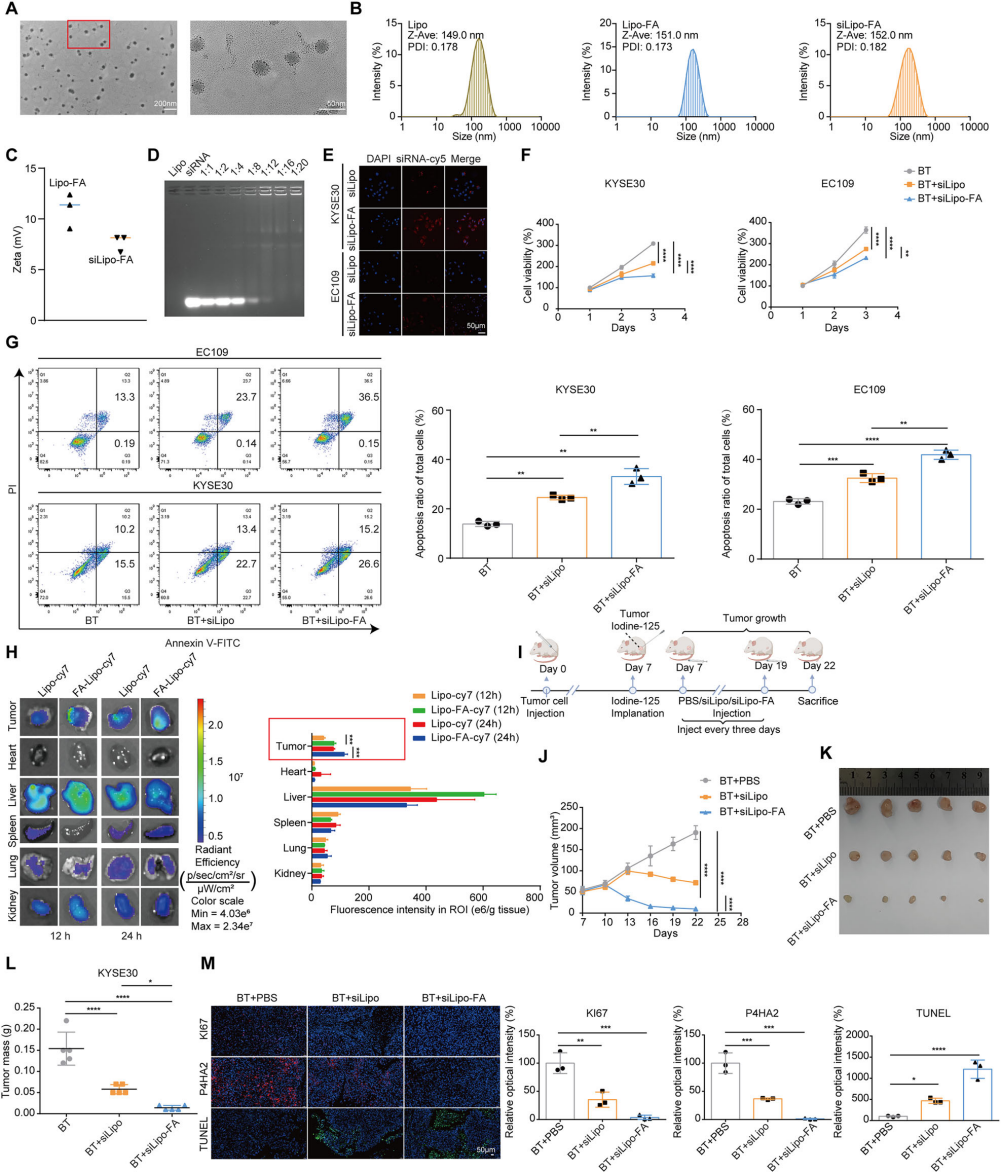
Folate-targeted cationic liposome carrying siRNA ameliorates the brachytherapy therapeutic effect. **A** TEM observation of siLipo-FA. Scale bars: 200 nm and 50 nm. **B** Size distribution of Lipo-FA, Lipo, and siLipo-FA. **C** Zeta potential distribution of Lipo-FA and siLipo-FA. **D** Agarose gel electrophoresis to evaluate the cationic liposome wrapped siRNA delivery ratio. The Cationic lipid (N)/nucleic acid molecule (P) ratios of siRNA to Lipo-FA were 1, 2, 4, 8, 12, 16, and 20. **E** Representative confocal images showing the uptake of siLipo and siLipo-FA by ESCC cells. Scale bar: 50 μm. **F** CCK-8 analysis of siLipo or siLipo-FA in the presence of ^125^I brachytherapy. **G** Flow cytometric analysis of the apoptosis of siLipo and siLipo-FA exposed to radiation. **H** Ex vivo Cy7 fluorescence images of mouse organs and tumors 12 or 24 h after NPs injection (left) and semi-quantitative analysis (middle). **I** Schematic diagram of synergistic therapy. **J** Changes in tumor volume in mice after tail vein injection of siLipo-FA, siLipo, and PBS. Photographs of tumors isolated on day 22 of treatment (**K**) and tumor weights of mice (**L**). **M** Representative IF images and quantitative fluorescence results evaluating KI67, TUNEL, and P4HA2 fluorescence intensity (middle) and quantitative data (right) in treated mouse tumors. Scale bar: 50 µm. Data were presented with mean ± SD, ****p < 0.0001; ***p < 0.001; **p < 0.01 and *p < 0.05; ns not significant.

In our endeavor to regulate P4HA2 function and assess its therapeutic potential in vivo, we have developed a folate-targeted liposome nanoplatform specifically designed for siRNA delivery, and we have evaluated its efficacy in inhibiting tumor growth. The physicochemical properties of the synthesized nanoliposomes were first investigated. The siRNA-loaded folate-targeting cationic liposome (siLipo-FA) exhibited a spherical structure under TEM (Fig. 7A). The average particle sizes of liposome (Lipo), liposome-FA(Lipo-FA), and siRNA-liposome-FA (siLipo-FA) were 149, 151, and 152 nm, respectively (Fig. 7B). The average zeta potential of Lipo-FA was 10.95 mV, which was reduced to 7.68 mV for siLipo-FA, due to the partial neutralization by the negatively charged P4HA2 siRNA (Fig. 7C). To evaluate the reproducibility of our nanoparticle formulation, we performed multiple batches of liposome preparation under identical conditions. The results indicated that the particle size remained consistent with no significant differences before and after siRNA loading, which suggested good batch-to-batch reproducibility (Fig. S6A). To obtain the optimal N/P ratio for the Lipo-FA/siP4HA2 formulation, the electrostatic interactions between Lipo-FA and siP4HA2 were evaluated by agarose gel electrophoresis. The Cationic lipid (N)/nucleic acid molecule (P) ratios of siRNA to Lipo-FA were 1, 2, 4, 8, 12, 16, and 20. The complete combination of siRNA and Lipo-FA, signified by the disappearance of bright bands on the gel, was achieved at an N/P ratio of 20 (Fig. 7D). These results indicated that the siLipo or siLipo-FA were well prepared and could be used for the following in vitro and in vivo evaluations. Next, the confocal microscopy images showed that the ESCC cells had a much higher uptake of siLipo-FA than siLipo (Fig. 7E). The CCK-8 assay results indicated that siLipo-FA was most effective in inhibiting cell growth under brachytherapy conditions (Fig. 7F). This observation was further corroborated by flow cytometry analysis, which measured the apoptosis rate of cells post-radiation (Fig. 7G). After establishing the efficacy of siLipo-FA in enhancing cellular responsiveness to brachytherapy, we first examined its biodistribution. Intravenous injection of Cy7-labeled Lipo and Lipo-FA in mice bearing tumors showed pronounced enrichment of the composite nanoparticles (NPs) in the tumor region 12 h post-injection. Notably, Lipo-FA enrichment in the tumor region was significantly higher than that of the Lipo group, especially after 24 h (Fig. 7H). We then evaluated whether NPs could effectively knock out P4HA2 in vivo and augment the anti-tumor effect post-^125^I implantation. Radioactive ^125^I was implanted into the central region of the tumor using needle guidance, 7 days after injecting esophageal cancer cells. Concurrently, the mice received intravenous treatments of 1 nmol siRNA every 3 days (Fig. 7I). After five consecutive treatments, the siLipo-FA group exhibited a more pronounced ability to inhibit tumor growth compared to the PBS and control NP groups (Fig. 7J–L). IF results further confirmed that siLipo-FA was the most effective in reducing the expression of P4HA2 and KI67 and inducing apoptosis (Fig. 7M). To further evaluate the potential in vivo side effects, we first tested the cytotoxicity of Lipo-FA liposomes and found that the amount injected into the mice was within the safe range (Fig. S6A). Blood parameter analysis in healthy mice, post 30 days intravenous administration of liposomes (1 nmol siRNA per mouse, *N* = 3), indicated that levels of alanine aminotransferase (Fig. S6B), aspartate aminotransferase (Fig. S6C), urea nitrogen (Fig. S6D), and creatinine (Fig. S6E) were within normal ranges. In addition, histological analysis showed no significant changes in the heart, liver, spleen, lung, and kidney tissues (Fig. S6F). Taken together, these results suggested that siLipo-FA demonstrates low in vivo toxicity and is an effective strategy for integrating si-P4HA2 delivery with ^125^I brachytherapy of esophageal cancer.

## Discussion

As one of the internal radiation therapies, brachytherapy (BT) can precisely deliver extremely high doses to target lesions with minimal damage to adjacent normal tissues, achieving superior tumor control over conventional external beam radiation therapy (EBRT) or stereotactic body radiation therapy (SBRT). In the case of esophageal obstruction, our team introduced BT combined with metal stents that can turn the treatment of luminal tumors from palliative to potentially curative with better tumor control. Unfortunately, most esophageal cancer patients will finally develop radioresistance, also commonly observed in patients with other cancers receiving BT. Our group and others have established a critical role of ER stress-induced autophagy in radioresistance. In this work, we proposed that P4HA2, an ER-related protein, mediated mitophagy via direct interaction with ATAD3A and protected ESCC cells from ionizing radiation damage. Molecular mechanisms revealed by this study hold the promise to design a strategy to overcome the resistance and achieve better treatment outcomes.

Our early findings indicated that ^125^I brachytherapy (BT) could induce reactive oxygen species (ROS)-mediated apoptosis and autophagy in human esophageal squamous cell carcinoma (ESCC) cells [8]. Moreover, BT enhanced autophagy triggered by endoplasmic reticulum stress, leading to ESCC developing resistance to brachytherapy [10]. ER stress-mediated mitophagy highlighted a potential direct interaction between the ER and mitochondria, leading to subsequent apoptosis. Even though several factors have been implicated in these processes, including those directly linking ER to mitochondria, this intricate biological process remains elusive. We noticed that P4HA2, an ER protein, was significantly upregulated in response to radiation and correlated with the prognosis of esophageal cancer.

P4HA2, a prolyl-4-hydroxylase subunit, has been widely recognized as an oncogenic gene that promotes tumor growth and metastasis in various cancers. In diseases such as cholangiocarcinoma, lung cancer, and hepatocellular carcinoma, P4HA2 promoted cell proliferation by activating collagen deposition [21, 47, 48]. The role of P4HA2 in promoting or inhibiting tumor metastasis varies between different cancers. For instance, P4HA2 inhibited metastasis in prostate cancer [29] but increased metastasis in cervical [22], breast [49], lung [48], and gastric cancers [50]. In this study, P4HA2 was observed to promote ESCC cell migration and invasion.

Some studies suggested that HIF-1 is a crucial transcription factor for P4HA2 [39]. Additionally, P4HA2 stabilizes HIF-1 protein, and HIF-1 also directly binds to the P4HA2 promoter, resulting in erdafitinib resistance in bladder cancer [18]. This suggests a close association between P4HA2 and hypoxic conditions. Previous research revealed an upregulation of the HIF-1 pathway in subcutaneous esophageal cancer after ^125^I radiation [7]. These results further emphasized the potential importance of P4HA2 in brachytherapy. The current study found that P4HA2 KD increases the sensitivity of ESCC cells to brachytherapy, while overexpression of P4HA2 leads to phenotypic recovery. Considering previous research showing that endoplasmic reticulum stress can activate autophagy, enhancing tumor cell resistance to BT, we discovered that P4HA2 can promote autophagy, augmenting the responsiveness of tumor cells to ^125^I brachytherapy radiation.

To the best of our knowledge, no reported role of P4HA2 has been linked to autophagy, particularly under endoplasmic reticulum (ER) stress. Our study revealed a novel mechanism by which P4HA2, through its interaction with ATAD3A, modulates mitophagy under radiation-induced ER stress. ER stress, triggered by misfolded protein accumulation, activates the unfolded protein response (UPR) to restore protein homeostasis. One of the key outcomes of ER stress is the induction of autophagy, a process that helps cells cope with damaged organelles and protein aggregates. In our study, we observed that ^125^I brachytherapy induced ER stress in esophageal squamous cell carcinoma (ESCC) cells, leading to the upregulation of P4HA2. This upregulation was associated with enhanced autophagy, which contributed to the development of resistance to brachytherapy.

The study confirmed P4HA2 as a new cofactor of ATAD3A, a critical mitochondrial-associated membranes (MAMs) component, using proteomic and co-immunoprecipitation approaches. ATAD3A was demonstrated to be associated with several mitochondrial functions [51], and it’s reasonable to postulate that P4HA2-ATAD3A interaction, at least partially, accounts for the ER-mitochondria interaction upon treatment with radiation. To fully understand to what extent the interaction between P4HA2 and ATAD3A contributes to mitophagy-mediated radiation resistance, more efforts are warranted to reveal (1) whether disruption of the P4HA2-ATAD3A interaction would rescue the radiation resistance; (2) whether and how radiation-induced upregulation of P4HA2 influences the physiological function of ATAD3A. ATAD3A has been demonstrated to influence PINK1-dependent mitophagy. Based on the subcellular localization of these two proteins, we speculated that they may form MAMs, although this requires further investigation. Previous studies have suggested that HIF-1 promotes cell survival by inducing autophagy-mediated mitochondrial degradation [52]. Several reports also indicated that ^125^I brachytherapy could elevate ROS levels in tumor cells, promoting mitophagy to resist ionizing radiation attacks [53]. Therefore, our research confirmed that P4HA2 promotes PINK1-dependent mitophagy, and the knockdown of P4HA2 could reverse radiation-induced autophagy and enhance the sensitivity of tumor cells to radiation. For the future, inhibition of P4HA2 may activate compensatory signaling pathways that promote tumor survival. For instance, the downregulation of P4HA2 could lead to the upregulation of other prolyl-hydroxylases or hypoxia-inducible factors (HIFs), which might sustain tumor growth and resistance. Additionally, the interplay between P4HA2 and ATAD3A in regulating mitophagy suggested that disruption of this interaction might trigger alternative mitochondrial quality control mechanisms, such as increased mitochondrial fission or fusion, to bypass the need for mitophagy.

In addition to transcriptional regulation, post-transcriptional modifications such as m^6^A methylation played a crucial role in gene expression. N^6^-methyladenosine (m^6^A) modification is an important post-translational modification of genes. m^6^A modification is modified by m^6^A methyltransferases or writers (such as METTL3/14/16, RBM15/15B, ZC3H3, VIRMA, CBLL1, WTAP, and KIAA1429) and is recognized by m^6^A-binding proteins including YTHDF1/2/3, YTHDC1/2, IGF2BP1/2/3, and HNRNPA2B1 [54]. Through correlation analysis using the TCGA database, we found that in esophageal cancer, P4HA2 has the most significant correlation with the m^6^A reading protein IGF2BP2. Further research indicated that IGF2BP2 mainly modified *P4HA2* in the 3′UTR region via m^6^A modification. These findings underscore the complexity of P4HA2 regulation and highlight the potential role of epitranscriptomic mechanisms in ESCC pathogenesis. Future studies should explore the interplay between transcriptional and post-transcriptional regulatory mechanisms in controlling P4HA2 expression and their implications for cancer progression and treatment.

Conventional gene silencing therapies are challenged by inherent drawbacks, including poor targeting, easy uptake by phagocytes, and degradation by endogenous nucleases [55]. Liposomes (LPs) are considered promising nanodrug delivery systems due to their low immunogenicity, stability, low toxicity, and low cost. However, due to the lack of tumor targeting, LPs drugs find it difficult to achieve a good therapeutic effect [56]. While the folate receptor is highly expressed in cancer cells and exhibits a strong affinity for its ligand folate, providing a more specific targeting mechanism for tumor cells compared to non-cancer cells [57]. To improve the efficacy of gene therapy, we proposed a concept validation: using folate actively targeted cationic liposomes for nuclear-targeted siRNA-P4HA2 delivery, to downregulate P4HA2 expression in vivo. Although our developed siLipo-FA delivery system effectively silenced P4HA2 expression in vitro and in vivo, there are still many challenges to overcome for clinical translation, including the controllable preparation and scalable production of nanoparticles (NP). Beyond scalability, challenges also exist in terms of liposome stability, in vivo release control, and targeting efficiency. In particular, the long-term stability of liposomes during storage, as well as their biodistribution and clearance characteristics in vivo, remain major obstacles for clinical application. Additionally, while liposomes could effectively encapsulate drugs, precise regulation of drug release within the body still requires further research. To improve the clinical feasibility of liposome-based systems, ongoing studies are focusing on enhancing liposome stability [58], optimizing drug release kinetics [59], and improving targeting capabilities [60]. Additionally, the long-term in vivo toxicity needs to be systematically assessed.

## Conclusion

In summary, a comprehensive analysis of our data leads to the conclusion that P4HA2 is a protein associated with sensitivity to brachytherapy. It exerts a significant anti-tumor effect by interacting with its molecular partner ATAD3A and regulating the level of cellular mitophagy through the PINK/parkin pathway (refer to schematic diagram). This study provides potential diagnostic and therapeutic targets for the treatment of refractory tumors using ^125^I in brachytherapy.

## Supplementary information

Supplementary material

Raw data for western blot of the supplementary materials

Raw data for western blot of the manuscript

patients ethics

animal ethics

STR for EC109 cell line

STR for KYSE30 cell line

STR for KYSE150 cell line

STR for TE-1 cell line

## Data Availability

The authors confirm that the data supporting the findings of this study can be found in the article and its supplementary materials.
