# Supplementary material for: P4HA2 interacted with ATAD3A to modulate PINK1/parkin-dependent mitophagy and 125I brachytherapy sensitization in esophageal carcinoma
Source: Cell Death Dis. 2025 Oct 6;16(1):685. doi: 10.1038/s41419-025-07864-x (PMC12501296; doi:10.1038/s41419-025-07864-x)
Supplement: Supplementary file 1 — Supplementary material [file 41419_2025_7864_MOESM1_ESM.docx]

**P4HA2 interacted with ATAD3A to modulate PINK1/parkin-dependent mitophagy and ^125^I brachytherapy sensitization in esophageal carcinoma**

Xijuan Yao^1#^, Cheng Feng^1#^, Xing Huang^2#^, Songzhe Wu^3^, Shuting Lu^4^, Yang Gao^5^, Tong Sun^1^, Xiaxing Bai^1^, Chenghui Li^1^, Kaizhi Jia^1^, Xue Han^1^, Zhongkai Wang^1^, Binda Chen^6^, Xiaobin Wang^7^, Jinhe Guo^1^*, and Jian Lu^1^*,

1. Center of Interventional Radiology & Vascular Surgery, Department of Radiology, Cultivation and Construction Site of the State Key Laboratory of Intelligent Imaging and Interventional Medicine (Southeast University), Basic Medicine Research and Innovation Center of Ministry of Education, Zhongda Hospital, Medical School, Southeast University, 87 Dingjiaqiao Road, Nanjing 210009, China
2. Department of Pathology, Jiangsu Cancer Hospital& Jiangsu Institute of Cancer Research& Nanjing Medical University Affiliated Cancer Hospital
3. School of Life Sciences and Technology, Advanced Institute for Life and Health, Southeast University, Nanjing 210096, China.
4. Department of Radiology, Nanjing First Hospital, Nanjing Medical University, No 68, Changle Road, Nanjing 210006, China
5. Department of Radiology, Jiangsu Cancer Hospital & Jiangsu Institute of Cancer Research & Affiliated Cancer Hospital of Nanjing Medical University, Nanjing, China.
6. JACO PHARMACEUTICALS CO., LTD
7. Laboratory Animal Center, Southeast University

^#^These authors contributed equally: Xijuan Yao, Cheng Feng, and Xing Huang.

* Correspondence authors:

Jian Lu, Ph.D., Center of Interventional Radiology & Vascular Surgery, Department of Radiology, Zhongda Hospital, Southeast University, 87 Dingjiaqiao Road, Nanjing 210009, China; E-mail: lujian43307131@126.com; Phone: +86-25-83262230.

Jinhe Guo, M.D., Center of Interventional Radiology & Vascular Surgery, Department of Radiology, Zhongda Hospital, Southeast University, 87 Dingjiaqiao Road, Nanjing 210009, China; E-mail: jinheguo@sina.com; Phone: +86-25-83262231.

**This supplementary information contains:**

- 19 Pages
- Supplementary Materials and Methods
- Supplementary Figures (7 Figures)
- Supplementary Tables (6 Tables)

**Supplementary Materials and Methods**

**Molecular Docking and Protein-Protein Interaction Analysis**

The potential binding sites and interaction details between P4HA2 and ATAD3A were performed using AlphaFold3, a state-of-the-art deep learning model for protein structure prediction(Accurate structure prediction of biomolecular interactions with AlphaFold 3). Five parallel predictions were conducted, and the structure with the highest predicted Template Modeling (pTM) score was selected for further analysis. The predicted complex structure was visualized and analyzed using PyMOL (version 3.0.1). The molecular interaction interface and binding characteristics between P4HC domain of P4HA2 and ATPase domain of ATAD3A were thoroughly examined. Final structural figures were prepared and rendered using Adobe Illustrator 2021 CC.

**Electrophoretic mobility shift assay(EMSA)**

Sangong Biotech (Shanghai, China) provided biotin-labeled RNA oligonucleotides. The LightShift^®^ Chemiluminescent EMSA Kit (Thermo Fisher Scientific, USA) was utilized to perform the RNA EMSA assay, following the guidelines provided by the manufacturer. For EMSA, 4 μg of IGF2BP2 protein (Abcam, USA) was incubated with 1 pmol biotin-labeled probe in the binding buffer for 20 min at room temperature. For competitive EMSA, 100 pmol of unlabeled probes were added into the reaction mixture 20 min prior to the addition of a constantamount of the labeled positive probe. The reaction mixtures were resolved on 6.0% non-denaturing polyacrylamide gels and transferred to nylon membranes (Servicebio, China). UV crosslinkingwas used to bind the RNA oligomers to the membrane, and the chemiluminescent imagingsystem was employed to detect the labeled probes.

**Supplementary Figures**


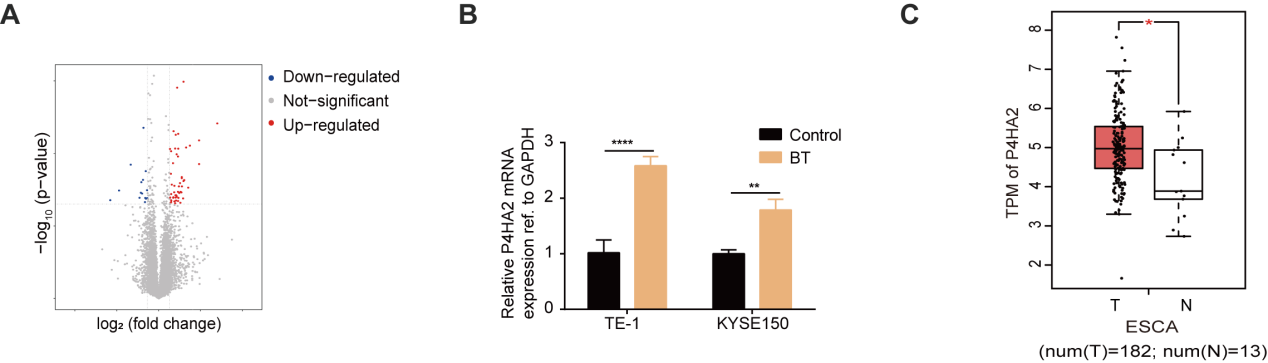


**Fig.S1. The upregulated level of P4HA2 ESCC patients treated with ^125^I.** (A) Volcano diagram of differentially expressed proteins (DEPs) between radiation cells and non-radiation cells. (B) Relative mRNA expression levels of P4HA2 in the irradiated group of TE-1 and KYSE150 cells. (C) GEPIA analysis of P4HA2 expression in esophageal cancer tissues versus normal tissues, including 13 normal and 182 neoplastic samples. Data was presented with mean ± SD, ****p < 0.0001; ***p < 0.001; **p < 0.01.


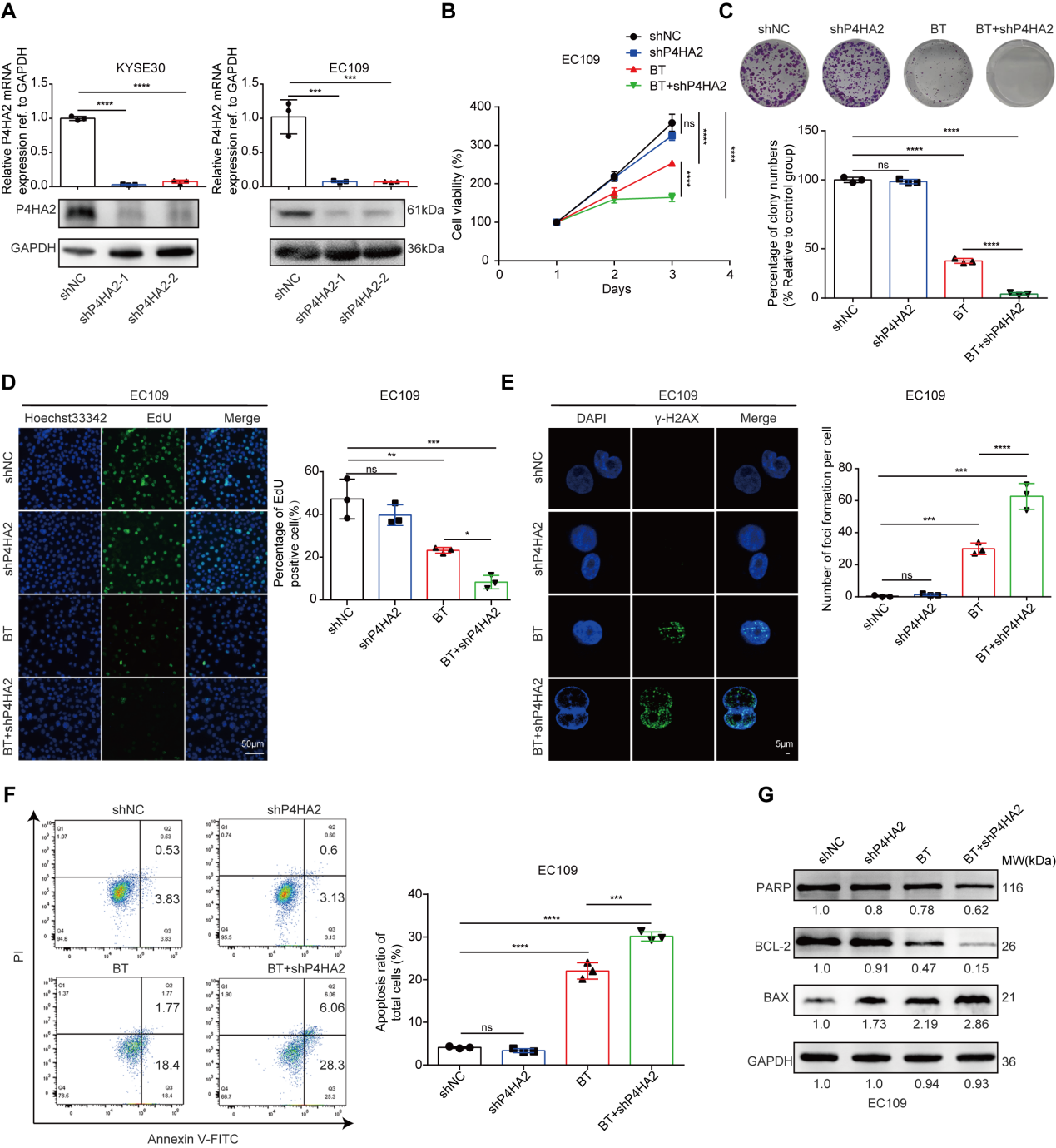


**Fig. S2. P4HA2 enhances the resistance of ESCC to ^125^I brachytherapy.** (A) Silencing of P4HA2 was performed by qRT-PCR (upper panel) and western blot (lower panel) in KYSE30 and EC109 cells. (B) CCK-8 assay and (C) Colony formation assays indicated the growth rate of EC109 cells transfected with lentivirus for P4HA2 knockdown with or without brachytherapy. (D) EdU incorporation assay was performed to evaluate DNA synthesis in ESCC cells. Green fluorescence represents EdU-positive cells, while blue fluorescence represents total cells. Scale bar: 50 μm. (E) Confocal images were used to observe the changes of γ-H2AX lesions in irradiated EC109 cells. Scale bar: 5 μm. (F) Apoptosis resulted from P4HA2 decreased in ESCC cells or co-treated with ^125^I brachytherapy. (G) WB showed PARP, BCL-2, and BAX protein levels after exposure to the radiation in different groups. Data was presented with mean ± SD, ****p < 0.0001; ***p < 0.001; **p < 0.01 and *p < 0.05; ns, not significant.

**
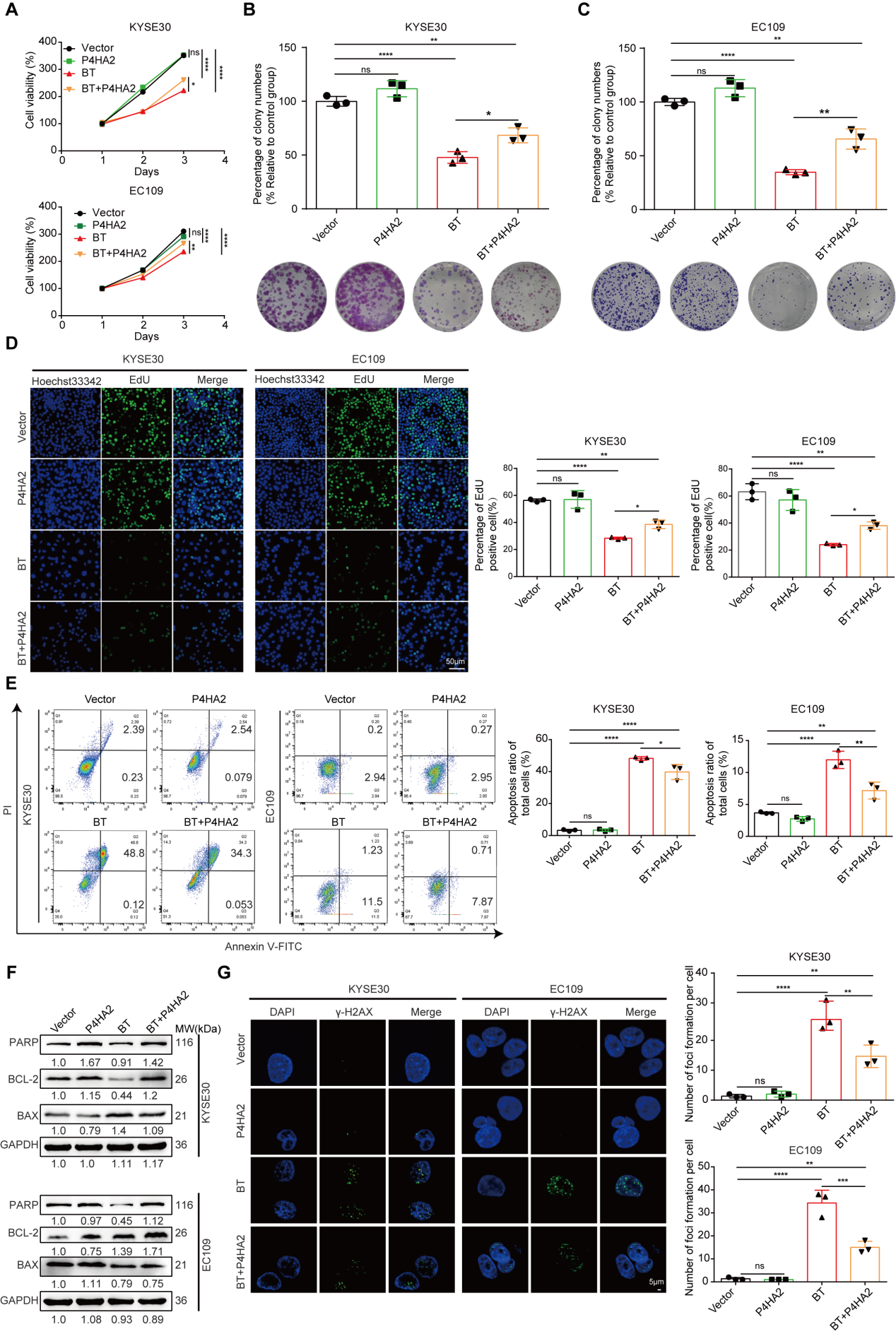
**

**Fig. S3. P4HA2 enhances the resistance of ESCC to ^125^I brachytherapy.** (A) CCK-8 assays and colony-formation assays to assess cell colony-forming capacity and survival of P4HA2-overexpressing cells after radiation exposure in Kyse30(B) and EC109 cells(C). (D) EdU incorporation assay was performed to evaluate DNA synthesis in ESCC cells. Scale, 50 μm. (E) Early and late apoptotic percentage cells and representative annexin V/PI staining plots were quantified by flow cytometry. (F) The protein levels of PARP, BCL-2, and BAX in ESCC cells with growing P4HA2 expression or synergistic brachytherapy were assessed by WB. (G) IF was used to assess the number of γ-H2AX lesions in irradiated ESCC cells overexpressing P4HA2. Scale bar: 5 μm. Data was presented with mean ± SD, ****p < 0.0001; ***p < 0.001; **p < 0.01 and *p < 0.05; ns, not significant.


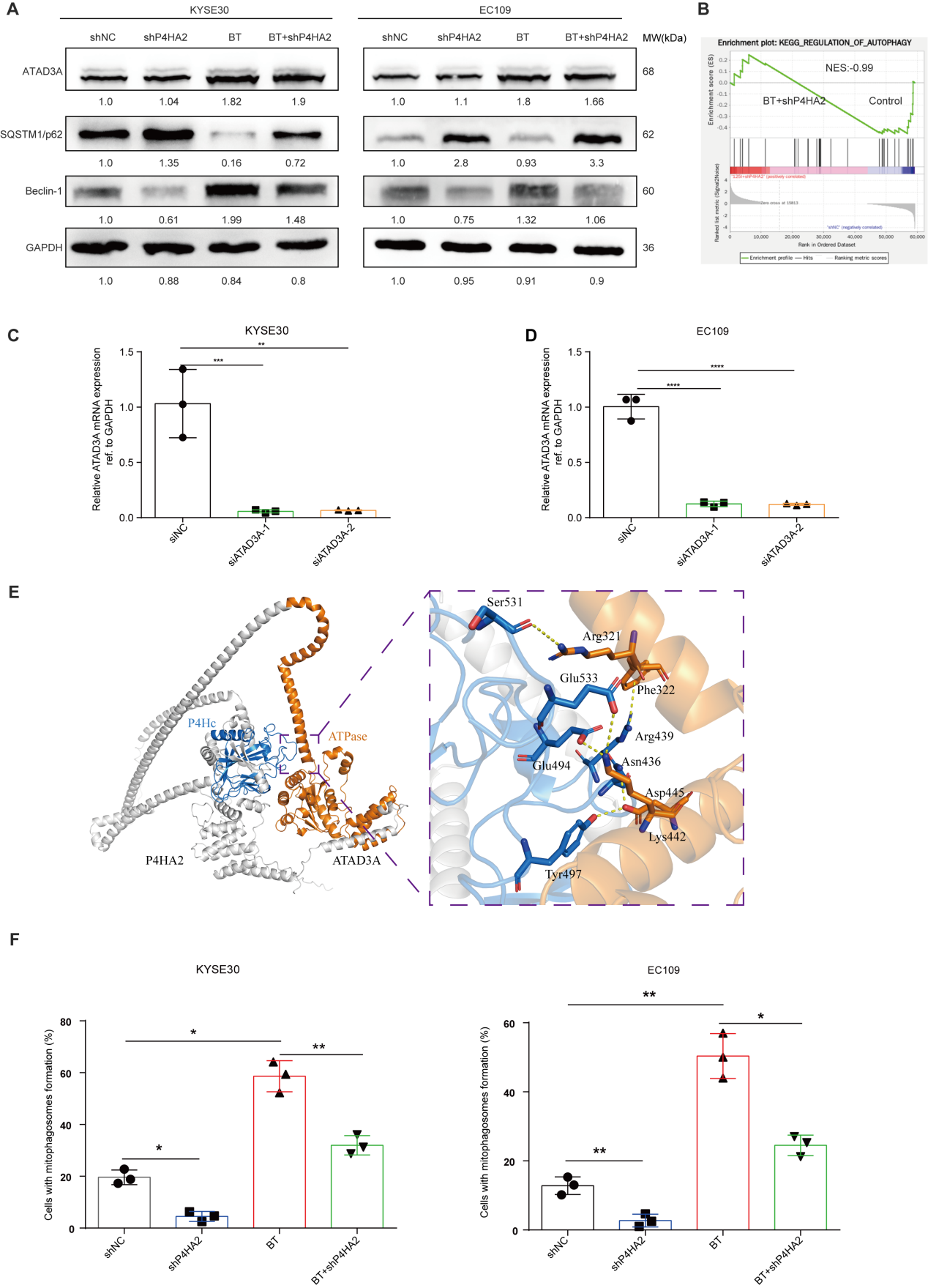


**Fig. S4. P4HA2 increased the resistance of ESCC to ^125^I through inducing autophagy.** (A) The protein levels of ATAD3A, SQSTM1/P62, and Beclin-1 in ESCC cells with decreasing P4HA2 expression or synergistic brachytherapy were assessed by WB. (B) GSEA analysis shows the enrichment of “Regulation of autophagy” among the BT+shP4HA2 vs control cells. The expression level of the ATAD3A gene in Kyse30 (C) and EC109 (D) cells was analyzed by qRT PCR. (E) Molecular docking of P4HA2 and ATAD3A. (F) Quantification immunofluorescence mitophagosomes (double-labeling LC3 and mitochondrial). Data was presented with mean ± SD, ****p < 0.0001; ***p < 0.001; **p < 0.01 and *p < 0.05; ns, not significant.


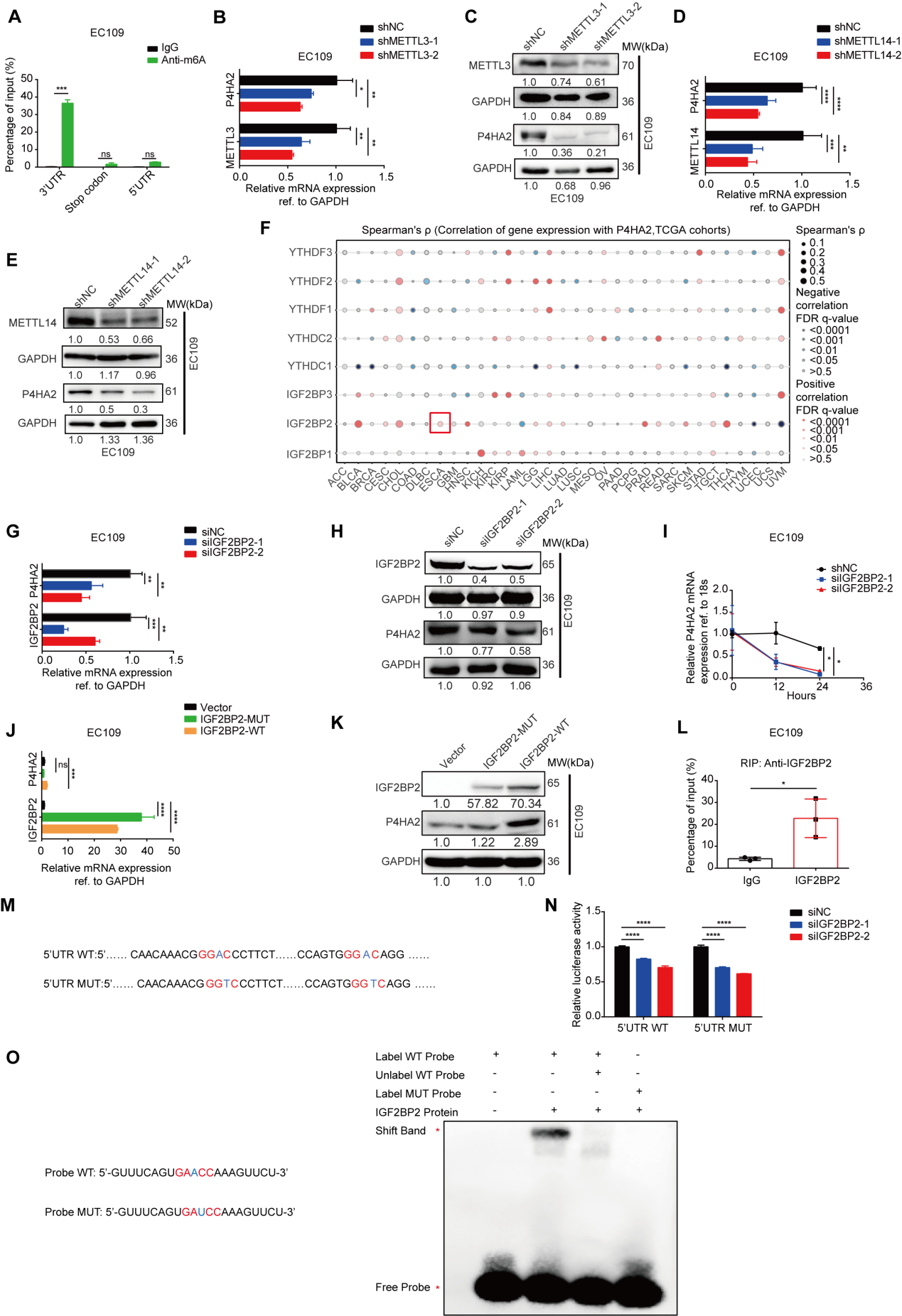


**Fig. S5. IGF2BP2 increases the expression of P4HA2 in an m^6^A-dependent manner.** (A) m^6^A RIP qRT-PCR showing enrichment of m^6^A modification at different positions in EC109 cell. qRT-PCR and western blot assays were employed to assess the gene and protein levels of P4HA2 in cells with METTL3 (B-C) and METTL14 (D-E) knockdown. (F) Spearman correlation analysis of P4HA2 and methylation-related genes in 33 cancer types in the TCGA dataset showed that IGF2BP2 was positively correlated with P4HA2 in esophageal cancer. qRT-PCR (G) and western blot (H) analysis of P4HA2 in IGF2BP2 KD cell lines. (I) The half-life of P4HA2 after treatment with 5 μM actinomycin D for the indicated times in the EC109 cell line with IGF2BP2 KD. qRT-PCR (J) and IB (K) analysis of P4HA2 expression in the EC109 cell with IGF2BP2 wild-type or mutant. (L) RIP qRT-PCR showed enrichment of IGF2BP2 in P4HA2 within the EC109 cell line. Schematic representation of P4HA2-5’UTR luciferase reporter mutation sites (M) and dual-luciferase assay results showing the luciferase activity of these constructs (N). (O) RNA-EMSA assay results showing that purified IGF2BP2 binds to the 3'UTR region of P4HA2. Data was presented with mean ± SD, ****p < 0.0001; ***p < 0.001; **p < 0.01 and *p < 0.05; ns, not significant.


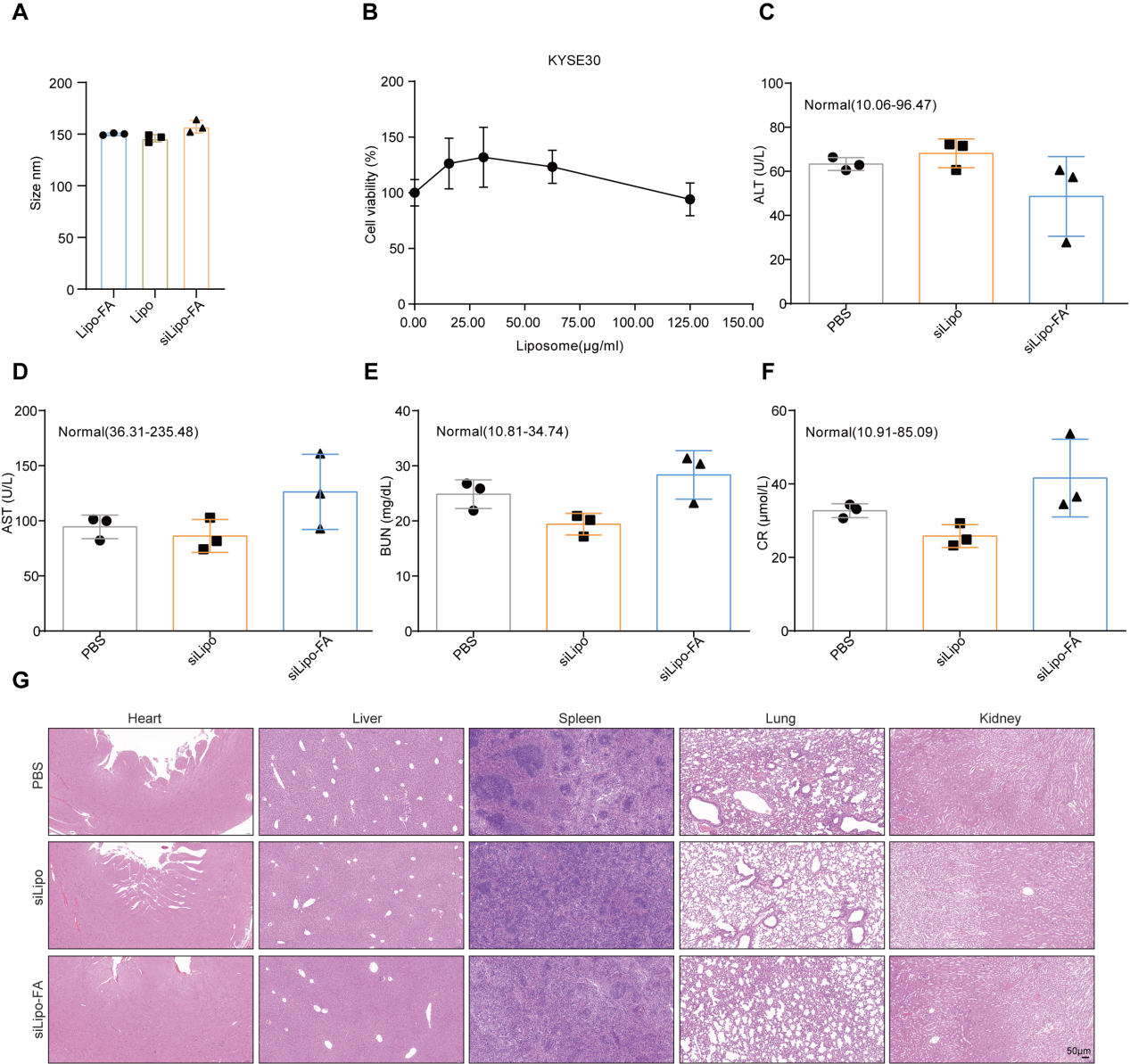


**Fig.S6. Safety assessment of siLipo-FA and siLipo *in vivo* and *in vitro*.**

(A) Size distribution of Lipo-FA, Lipo, and siLipo-FA. (B) CCK-8 was used to detect the toxicity of different concentrations of Liposome to ESCC cells. (C) Quantitative data of ALT(B)/AST(D) in the blood of mice after 30 days of PBS/siLipo/ siLipo-FA intravenously injected. The normal range in healthy mice was 10.06-96.47 U/L and 36.31-235.48 U/L, respectively. Quantitative data of blood BUN(E) and CR(F) in mice after different treatment groups. The normal ranges were 10.81-34.74 mg/dL and 10.91-85.09 µmol/L, respectively. (G) HE stains of heart, liver, spleen, lung, and kidney in different groups of mice. Scale bar: 50 µm.


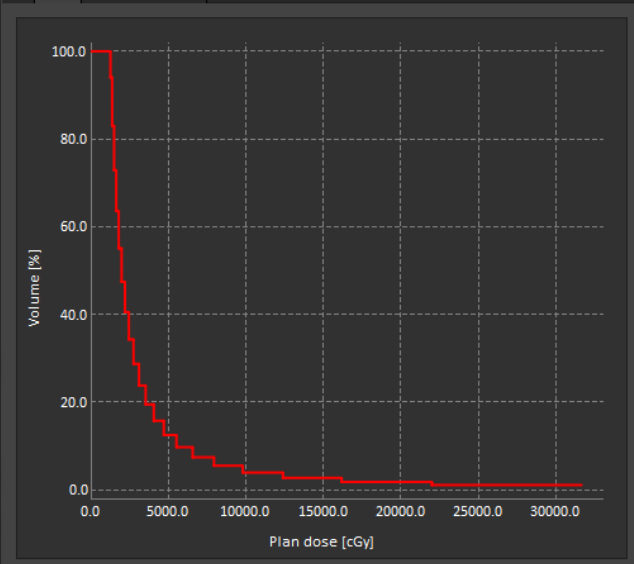


**Fig.S7.** **Dose-volume histogram (DVH).** After implanting a 29.6 MBq particle into a subcutaneous xenograft in mice via percutaneous puncture, the dose-volume histogram (DVH) obtained through calculations by the TPS system (developed by Beihang University, Beijing).

**Supplementary Tables**

**Table S1. Correlations Between P4HA2 Expression and Clinical Characteristics in 185 ESCC Patients**

| Variables | Total  No. (%) | P4HA2 expression | | P-value |
| --- | --- | --- | --- | --- |
|  |  | Low group  No. (%) | High group No. (%) |  |
| Number of patients | 185 | 70 (37.8) | 115 (62.2) |  |
| Gender |  |  |  | 0.875 |
| Male | 32 (17.3) | 13 (18.6) | 19 (16.5) |  |
| Female | 153 (82.7) | 57 (81.4) | 96 (83.5) |  |
| Age (years) |  |  |  | 0.198 |
| ≤60 | 51 (27.6) | 15 (21.4) | 36 (31.3) |  |
| ＞60 | 134 (72.4) | 55 (78.6) | 79 (68.7) |  |
| Pathologic_T |  |  |  | 0.015 |
| T1 | 40 (21.6) | 23 (32.9) | 17 (14.8) |  |
| T2 | 38 (20.5) | 13 (18.6) | 25 (21.7) |  |
| T3 | 107 (57.8) | 34 (48.6) | 73 (63.5) |  |
| Pathologic_N |  |  |  | 0.037 |
| N0 | 59 (31.9) | 26 (37.1) | 33 (28.7) |  |
| N1 | 72 (38.9) | 31 (44.3) | 41 (35.7) |  |
| N2 | 46 (24.9) | 13 (18.6) | 33 (28.7) |  |
| N3 | 8 (4.3) | 0 (0.0) | 8 (7.0) |  |
| Metastasis |  |  |  | NA |
| M0 | 185 (100.0) | 70 (100.0) | 115 (100.0) |  |
| Tumor stage |  |  |  | 0.027 |
| I | 29 (15.7) | 13 (18.6) | 16 (13.9) |  |
| II | 64 (34.6) | 31 (44.3) | 33 (28.7) |  |
| III | 92 (49.7) | 26 (37.1) | 66 (57.4) |  |
| Lymphovascular space invasion |  |  |  | 1.000 |
| Yes | 126 (68.1) | 48 (68.6) | 78 (67.8) |  |
| No | 59 (31.9) | 22 (31.4) | 37 (32.2) |  |
| Perineural invasion |  |  |  | 0.683 |
| Yes | 141 (76.2) | 55 (78.6) | 86 (74.8) |  |
| No | 44 (23.8) | 15 (21.4) | 29 (25.2) |  |
| Smoke |  |  |  | 0.99 |
| Smoking | 99 (53.5) | 38 (54.3) | 61 (53.0) |  |
| Non-smoking | 86 (46.5) | 32 (45.7) | 54 (47.0) |  |
| Drink |  |  |  | 0.699 |
| Drinking | 109 (58.9) | 43 (61.4) | 66 (57.4) |  |
| Non-drinking | 76 (41.1) | 27 (38.6) | 49 (42.6) |  |

**Table S2. Clinical information of 12 ^125^I brachytherapy radioresistant (BT-RR) ESCC patients and 12 brachytherapy radiosensitive (BT-RS) patients**

| Variables | Total  No. (%) | BT-RR group | BT-RS group | P-value |
| --- | --- | --- | --- | --- |
| Number of patients | 24 | 12 (50.0) | 12 (50.0) |  |
| Gender |  |  |  | 1.000 |
| Male | 20 (83.3) | 10 (83.3) | 10 (83.3) |  |
| Female | 4 (16.7) | 2 (16.7) | 2 (16.7) |  |
| Age (years) |  | 75.00±9.56 | 76.92±8.27 | 0.554 |
| Sites |  |  |  | 0.223 |
| Upper | 5 (20.8) | 3 (25.0) | 2 (16.7) |  |
| Middle | 13 (54.2) | 8 (66.7) | 5 (41.7) |  |
| Lower | 6 (25.0) | 1 (8.3) | 5 (41.7) |  |
| Histology |  |  |  | 1.000 |
| Squamous cell carcinoma | 24 (100.0) | 12 (100.0) | 12 (100.0) |  |
| Histologic grade |  |  |  | 0.278 |
| 1 | 2 (8.3) | 2 (16.7) | 0 (0.0) |  |
| 2 | 17 (70.8) | 8 (66.7) | 9 (75.0) |  |
| 3 | 5 (20.8) | 2 (16.7) | 3 (25.0) |  |
| Clinical tumor stage |  |  |  | 0.180 |
| I | 1 (4.2) | 0 (0.0) | 1 (8.3) |  |
| II | 7 (29.2) | 4 (33.3) | 3 (25.0) |  |
| III | 9 (37.5) | 2 (16.7) | 7 (58.3) |  |
| IV | 7 (29.2) | 6 (50.0) | 1 (8.3) |  |
| Clinical_T |  |  |  | 0.125 |
| T1 | 1 (4.2) | 0 (0.0) | 1 (8.3) |  |
| T2 | 7 (29.2) | 3 (25.0) | 4 (33.3) |  |
| T3 | 13 (54.2) | 6 (50.0) | 7 (58.3) |  |
| T4 | 3 (12.5) | 3 (25.0) | 0 (0.0) |  |
| Clinical_N |  |  |  | 0.310 |
| N0 | 7 (29.2) | 3 (25.0) | 4 (33.3) |  |
| N1 | 6 (25.0) | 3 (25.0) | 3 (25.0) |  |
| N2 | 5 (20.8) | 1 (8.3) | 4 (33.3) |  |
| N3 | 6 (25.0) | 5 (41.7) | 1 (8.3) |  |
| Metastasis |  |  |  | 1.000 |
| M0 | 22 (91.7) | 11 (91.7) | 11 (91.3) |  |
| M1 | 2 (8.3) | 1 (8.3) | 1 (8.3) |  |

**Table S3. Mass spectrometry analysis of P4HA2-associated proteins.**

| Accession | Protein Identity | # Unique Peptides | # Peptides | # PSMs |
| --- | --- | --- | --- | --- |
| Q05DA4 | P4HA2 | 13 | 13 | 20 |
| Q9NVI7 | ATAD3A | 5 | 5 | 5 |

**Table S4. List of qRT-PCR primers.**

|  | Forwards (5'-3') | Reverses (5'-3') |
| --- | --- | --- |
| ANLN | TCAGACCCAAAGGTTGAGCA | ACTGTTTGTGCCAATGGTGC |
| PURB | TTCTTGGTGCGTGAGAACCG | CTCCGGGAGCTCTCCATACA |
| CDCA8 | GCAGGAGAGCGGATTTACAAC | CTGGGCAATACTGTGCCTCTG |
| ACAD8 | GGCTGATATGGCAACAAGGC | TCCTCATCACTTCATTGCTACCT |
| IGF2BP2 | AGCTAAGCGGGCATCAGTTTG | CCGCAGCGGGAAATCAATCT |
| METTL3 | TTGTCTCCAACCTTCCGTAGT | TTGTCTCCAACCTTCCGTAGT |
| METTL14 | TGGTTCAAGTGACACTACCA | TTGGTTGGACTACTTTCTGCTA |
| ATAD3A | CAAGAATGCCACGCTTGTCG | GTACAGGCTGCGGTTCTTCT |
| P4HA2 | CAAACTGGTGAAGCGGCTAAA | GCACAGAGAGGTTGGCGATA |
| P4HA2-5’UTR | GAGACCCGTATAATTCGTTAA | TCTTCCAATCCGCCTGTC |
| P4HA2-Stop codon | CACAATAGATAGAAATGCCATAA | CACAATAGATAGAAATGCCATAA |
| P4HA2-3’UTR | TTGACATGGGCTGAAGGA | TTGACATGGGCTGAAGGA |
| GAPDH | GGAGCGAGATCCCTCCAAAAT | TGCCAGAGTCTCGTTCGTTATCG |
| 18S | CGGACAGGATTGACAGATTGATAGC | CCACAAATTATGCAGTCGAGTTTCCC |

**Table S5. Sequences of siRNA/shRNA for KD.**

| Forwards (5’-3’) | |
| --- | --- |
| Si-P4HA2-1 | CGAAUUCUUCACCUCUAUUTT |
| Si-P4HA2-2 | GGUACUUUGAGCAGUUAUUTT |
| Si-IGF2BP2-1 | GCGAAAGGAUGGUCAUCAUTT |
| Si-IGF2BP2-2 | GGGACCAAGAUAACAAUCUTT |
| Si-ATAD3A-1 | GCUUGUCGCCGGCCGCUUCTT |
| Si-ATAD3A-2 | GGAACAUCCUGAUGUACGGTT |
| Sh-P4HA2-1 | GCCGAATTCTTCACCTCTATT |
| Sh-P4HA2-2 | GCAGTCTCTGAAAGAGTACAT |
| Sh-METTL3-1 | GATGAGTCTTTAGGTGACTGCTCTT |
| Sh-METTL3-2 | GCTACCTGGACGTCAGTATCT |
| Sh-METTL14-1 | GATCCGGATGAAGGAGAGACAGATGATTCAAGAGATCATCTGTCTCTCCTTCATCCTTTTTTG |
| Sh-METTL14-2 | GATCCGCTGGACTTGGGATGATATTATTCAAGAGATAATATCATCCCAAGTCCAGCTTTTTTG |

**Table S6. List of primary antibodies.**

| Antibodies | Manufacturer | Application |
| --- | --- | --- |
| P4HA2 | Abcam, #ab70887 | 1:1000 for WB, 3μg for IP |
| P4HA2 | Proteintech, # 13759-1-AP | 1:400 for ICC/IF |
| SQSTM1/P62 | Abcam, #ab109012 | 1:1000 for WB |
| Beclin 1 | Proteintech, # 11306-1-AP | 1:1000 for WB |
| Bcl2 | Proteintech, #68103-1-lg | 1:1000 for WB |
| BAX | Proteintech, # 50599-2-Ig | 1:1000 for WB |
| PARP | Cell Signaling Technology,  # 9532S | 1:1000 for WB |
| LC3 II | Cell Signaling Technology, #43566T | 1:1000 for WB,1:200 for IF |
| ATAD3A | Proteintech, #16610-1-AP | 1:1000 for WB, 3μg for IP, 1:400 for ICC/IF |
| IGF2BP2 | Proteintech, # 116001-1-AP | 1:1000 for WB |
| METTL3 | Proteintech, # 15073-1-AP | 1:1000 for WB |
| METTL14 | Proteintech, # 26158-1-AP | 1:1000 for WB |
| PINK1 | Proteintech, #23274-AP | 1:250 for WB, 1:200 for IF |
| PARK2/Parkin | Proteintech, #14060-1-AP | 1:1000 for WB |
| GAPDH | Proteintech, #60004-1-lg | 1:2000 for WB |
| HA | Proteintech, #66006-2-Ig | 1:2000 for WB |
| FLAG | Proteintech, # 20543-1-AP | 1:2000 for WB |
| Anti-gamma H2AX | Abcam, #ab 81299 | 1:400 for ICC/IF |
