# Supplementary material for: P4HA2 interacted with ATAD3A to modulate PINK1/parkin-dependent mitophagy and 125I brachytherapy sensitization in esophageal carcinoma
Source: Cell Death Dis. 2025 Oct 6;16(1):685. doi: 10.1038/s41419-025-07864-x (PMC12501296; doi:10.1038/s41419-025-07864-x)

**Raw data of western blot**

Figure S2:


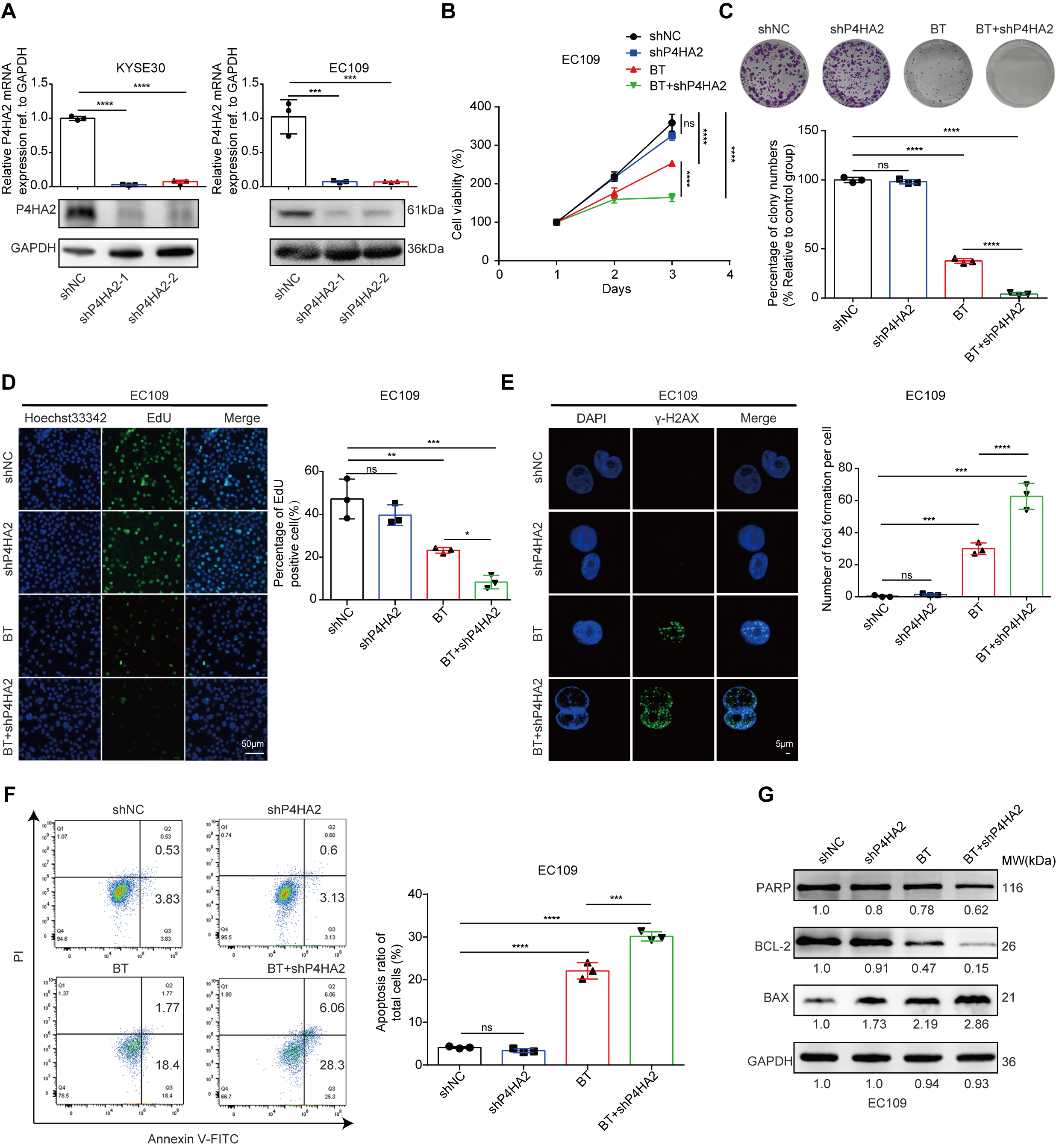


1. Left:
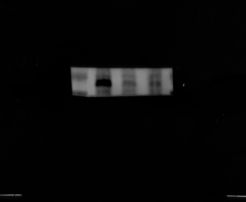

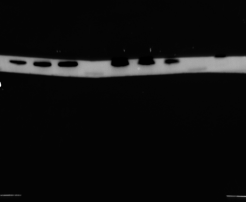

2. right:
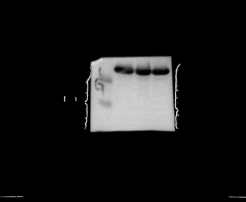

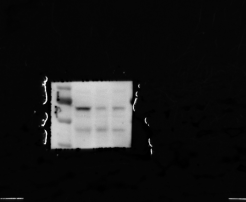


G-PARP:
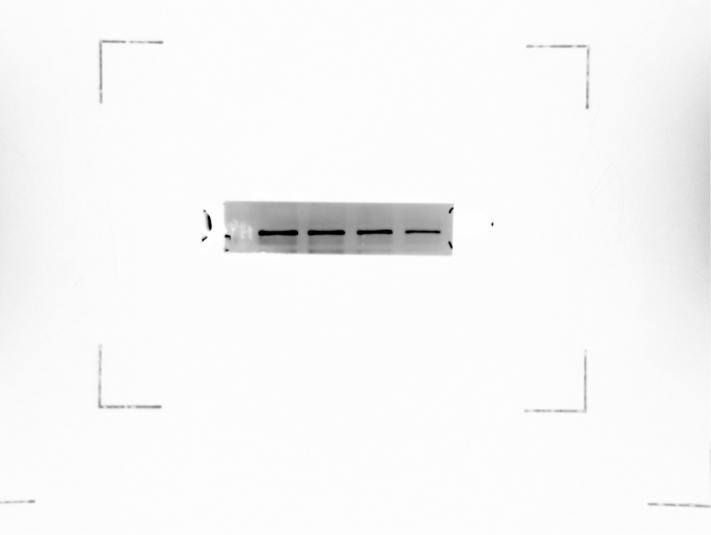


G-BCL-2:
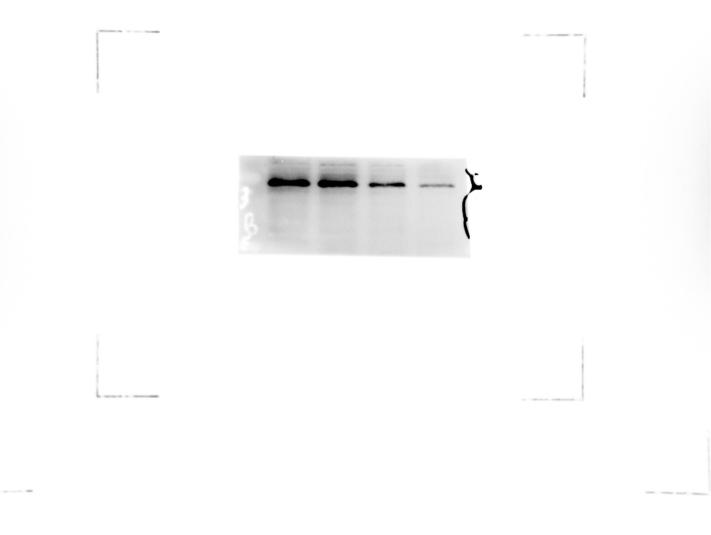


G-BAX:
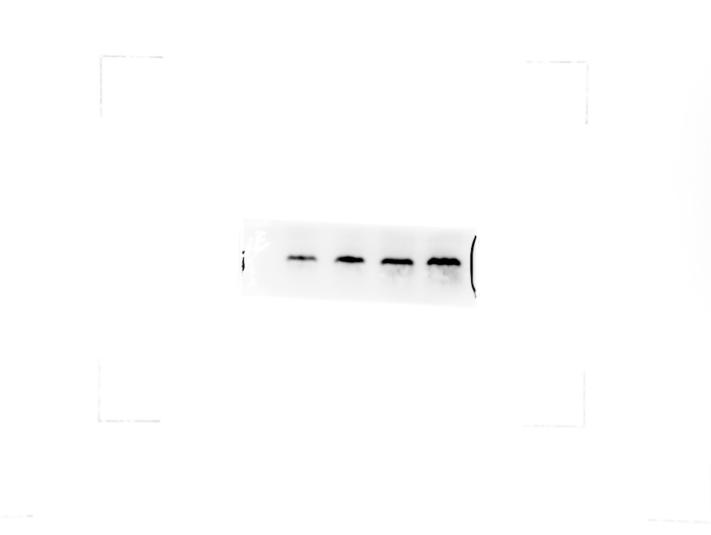


G-GAPDH:
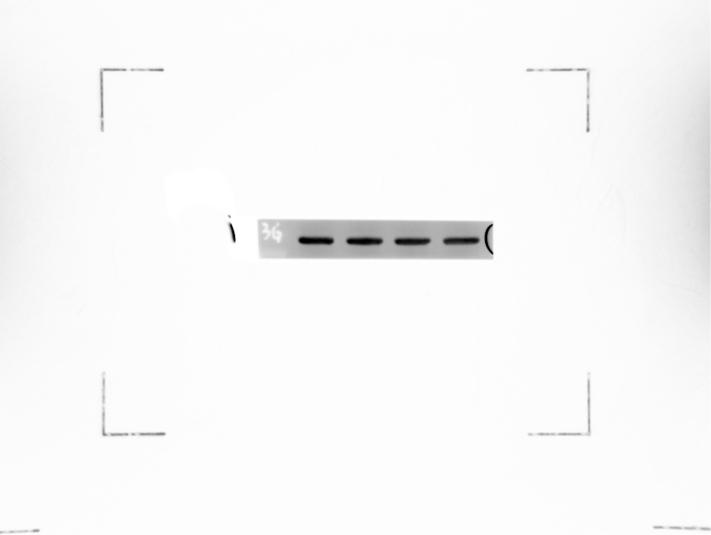


Figure S3F:


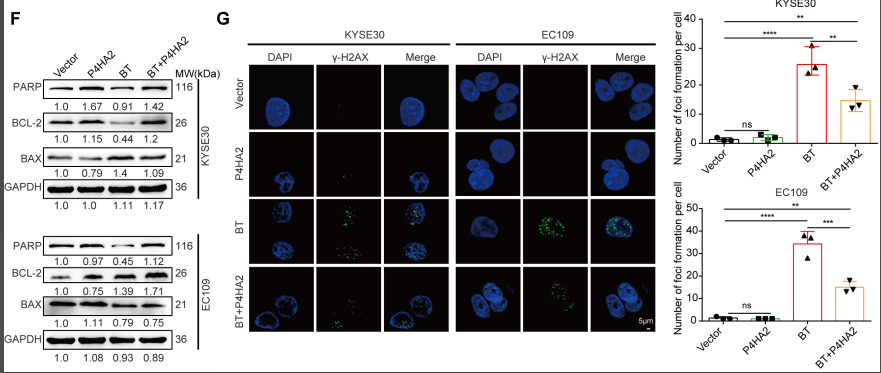


F-KYSE30:


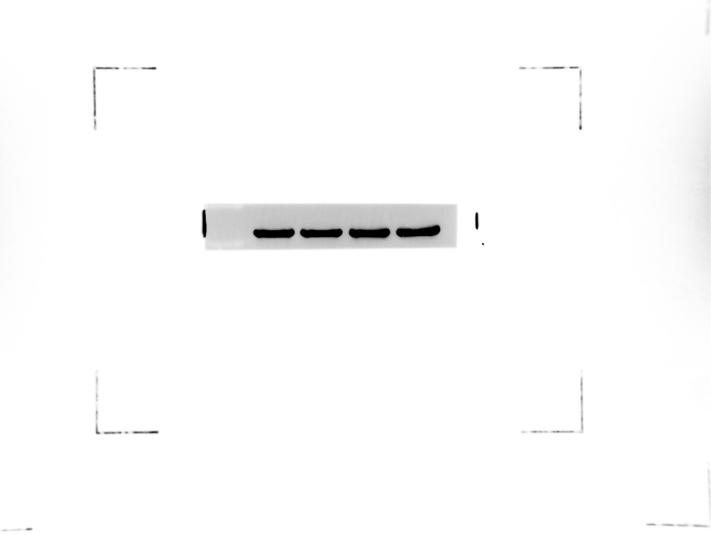

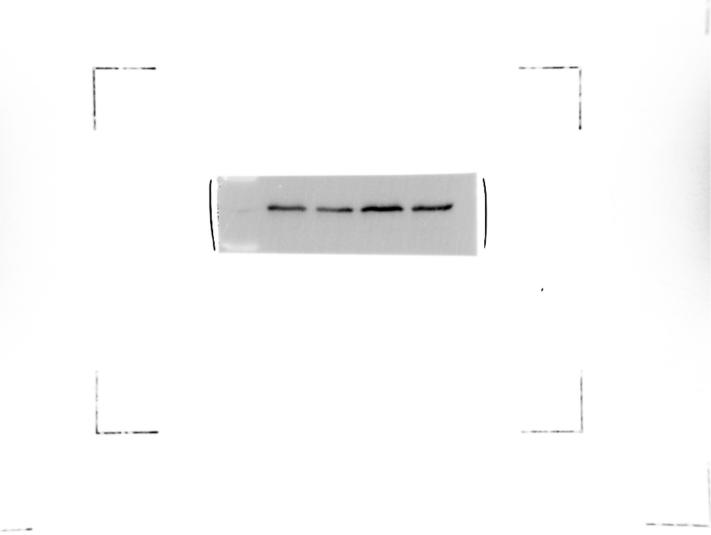

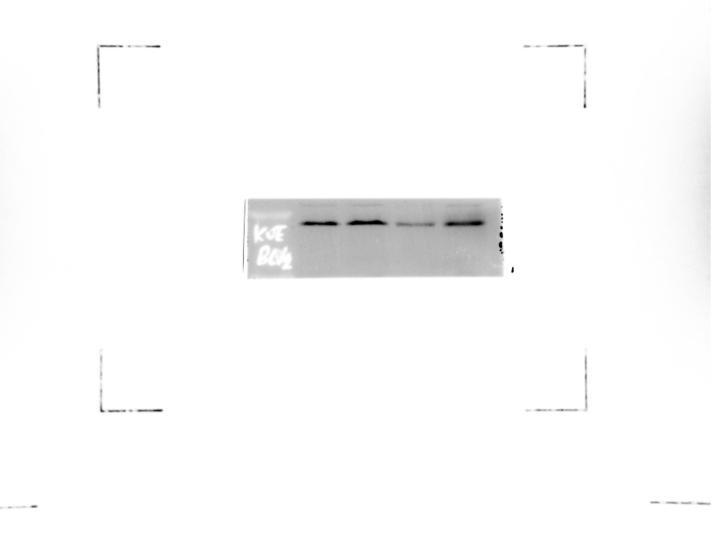

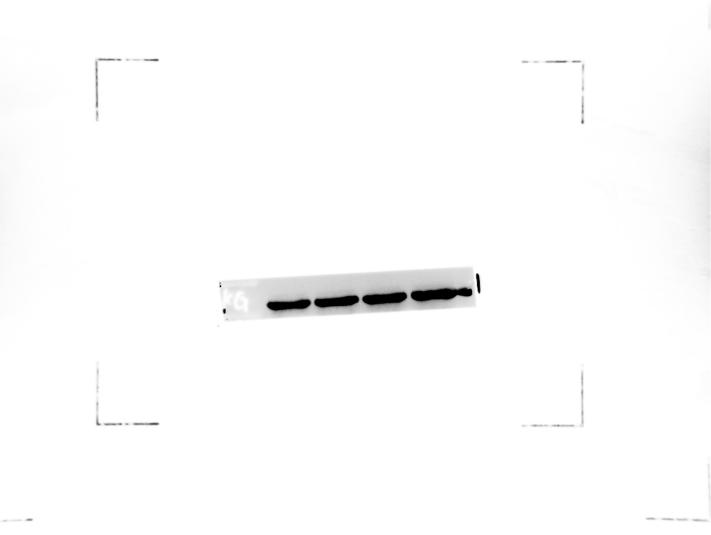

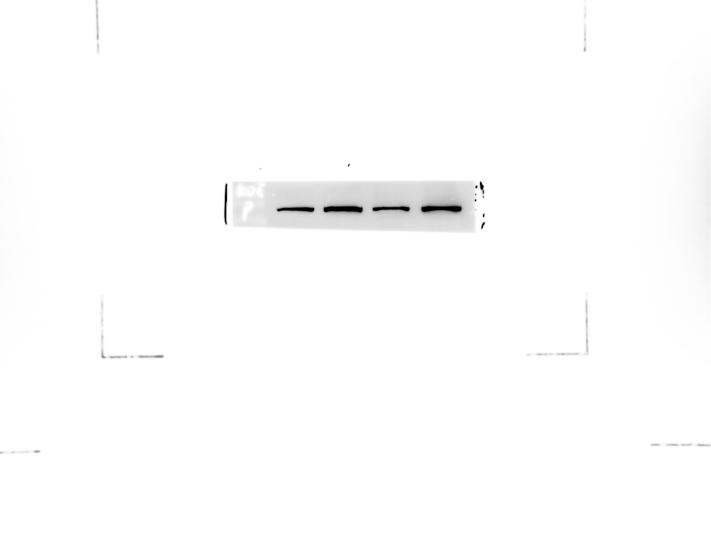


F-EC109:


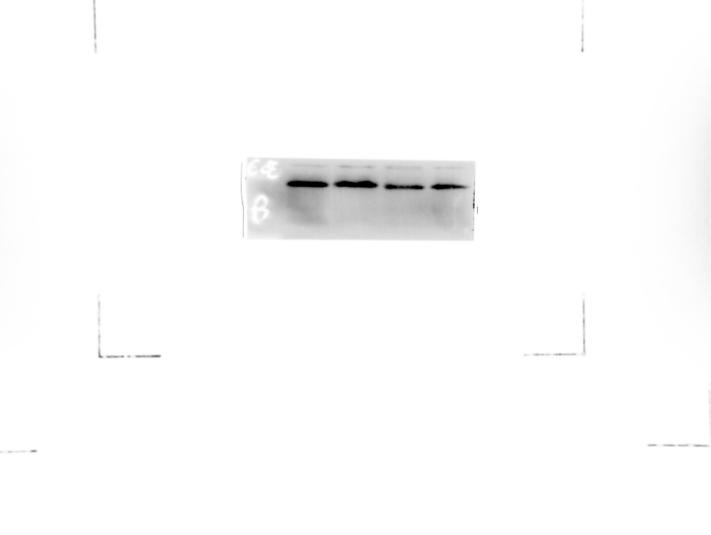

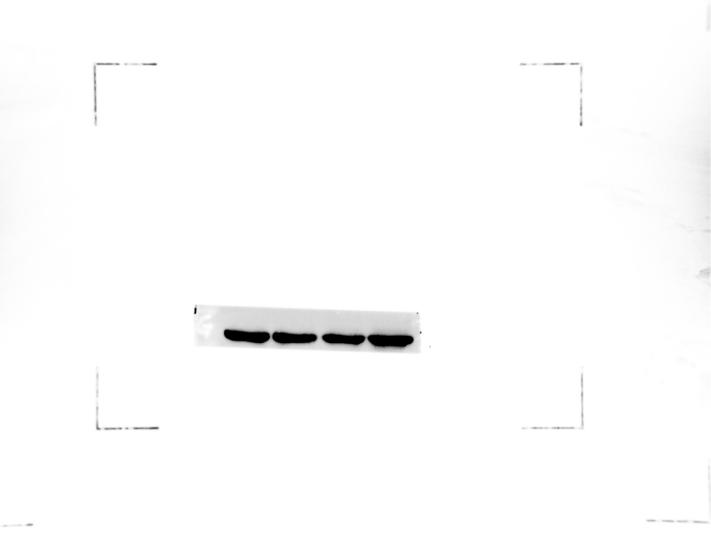

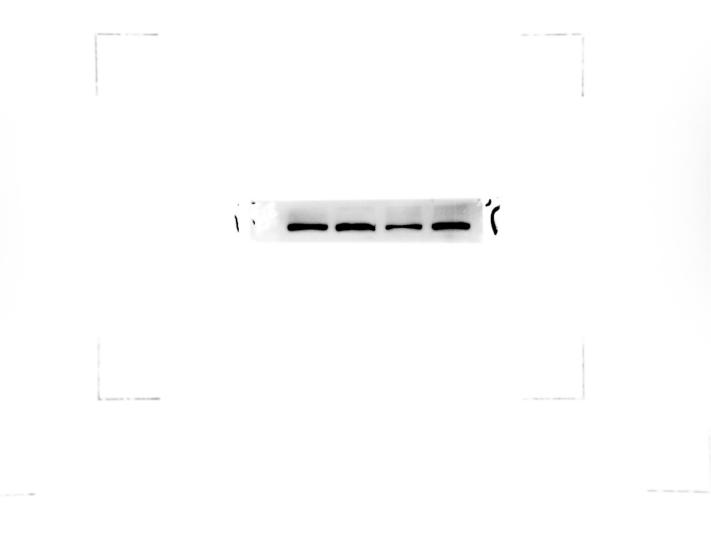

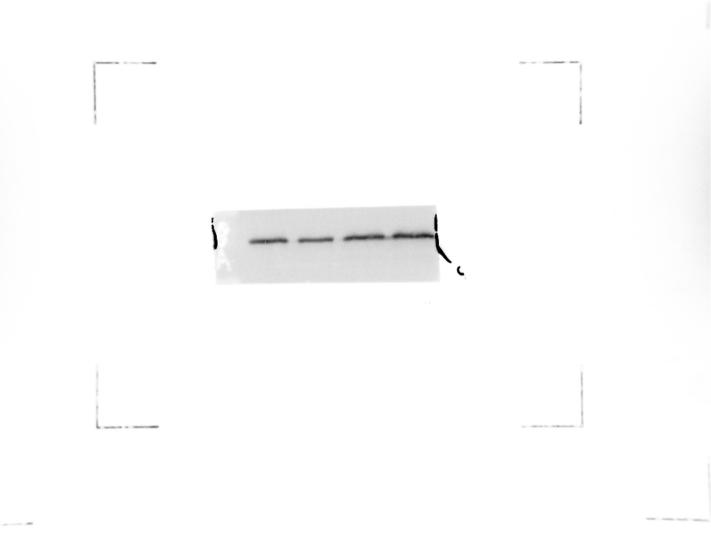

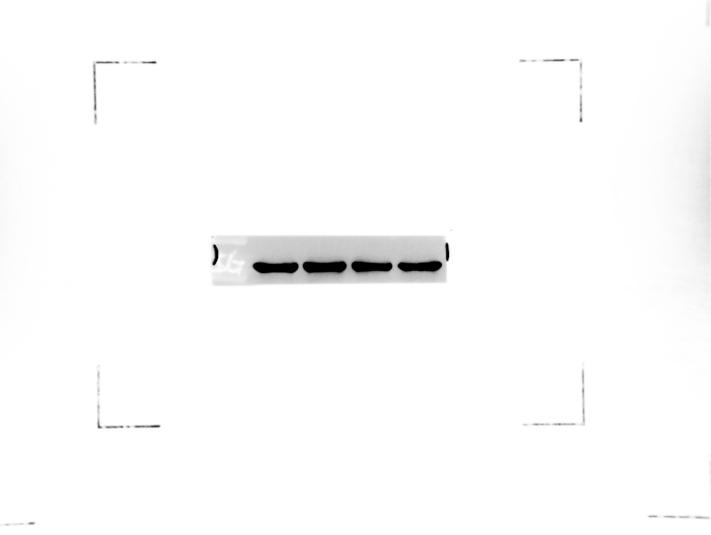


Figure S4A:


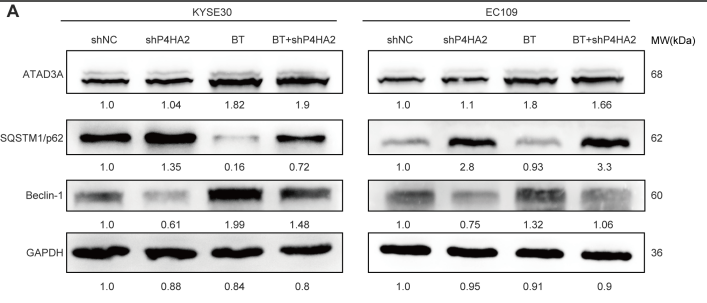


1. KYSE30:


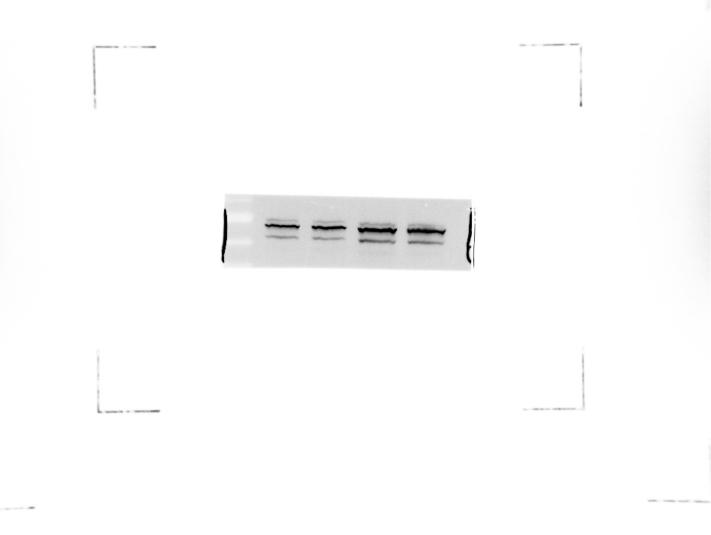


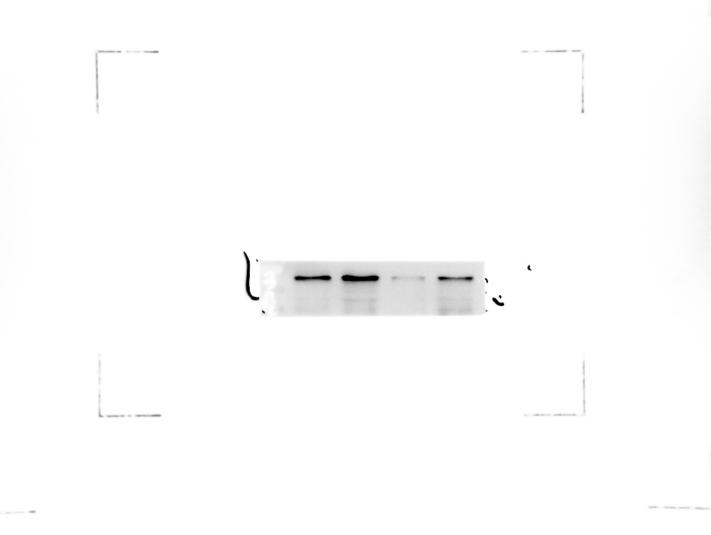


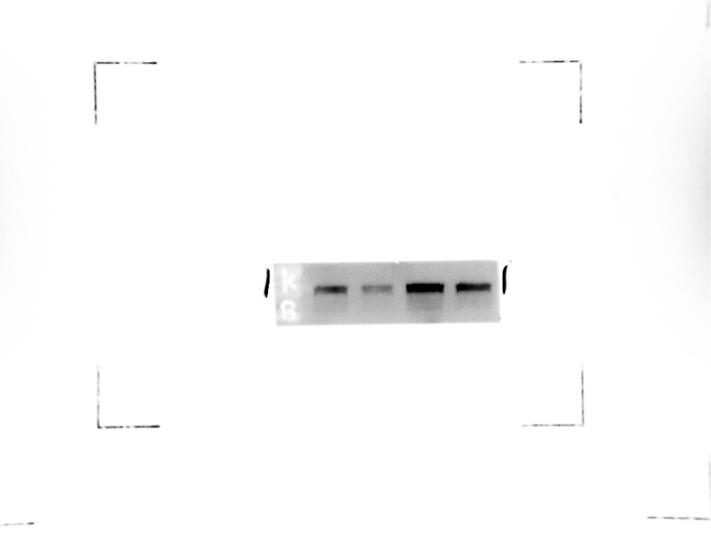


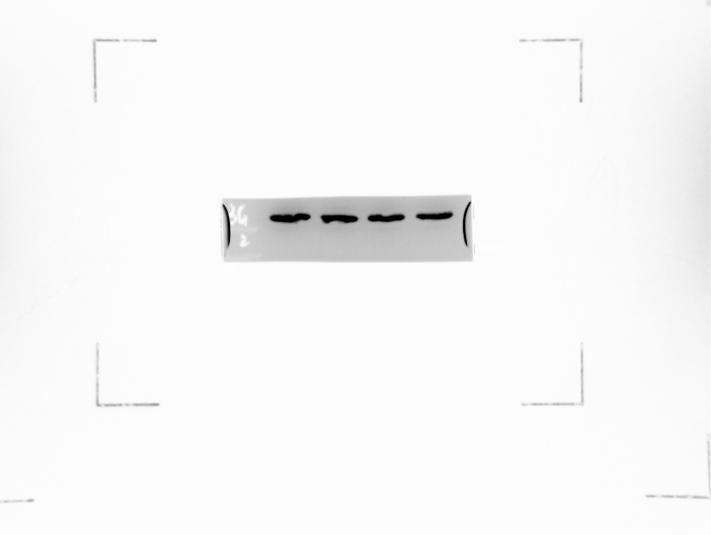


1. EC109:


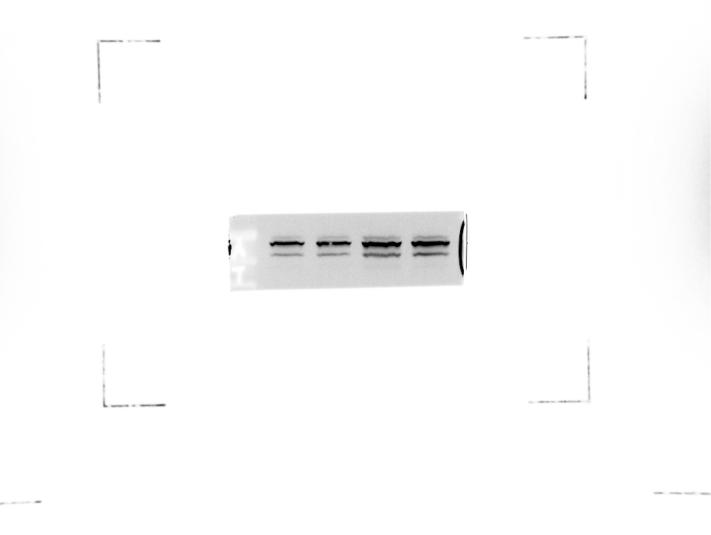

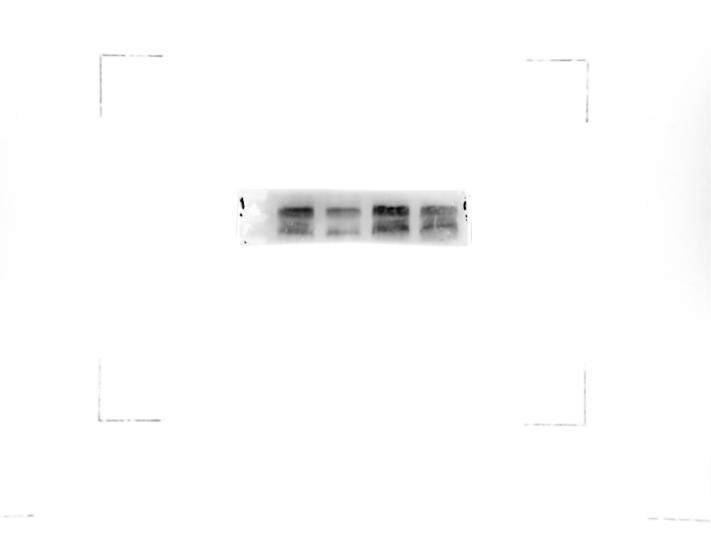

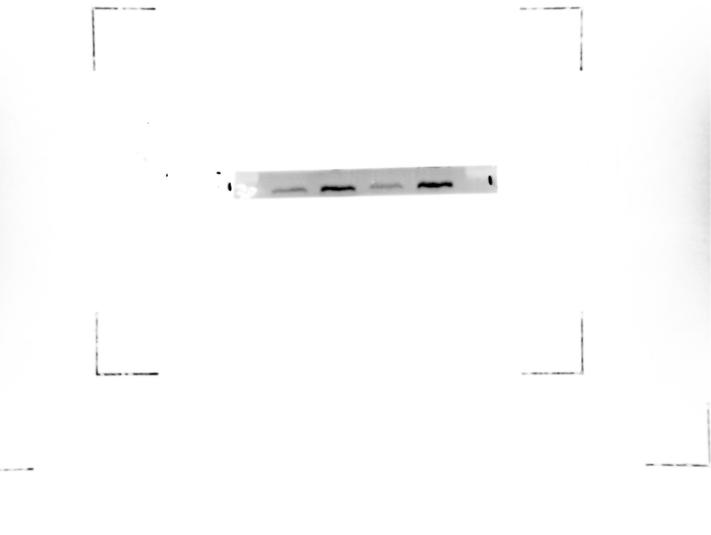

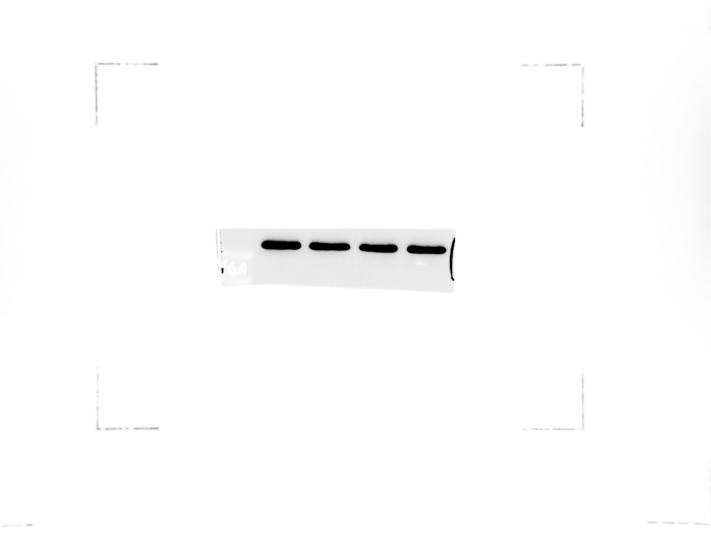

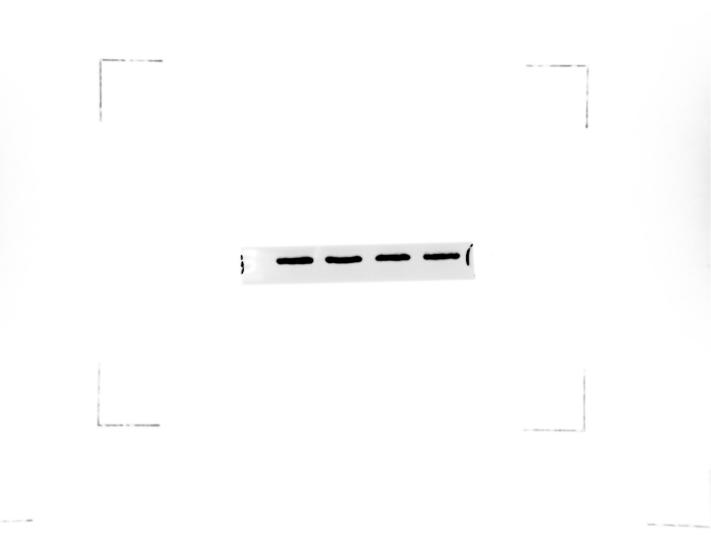

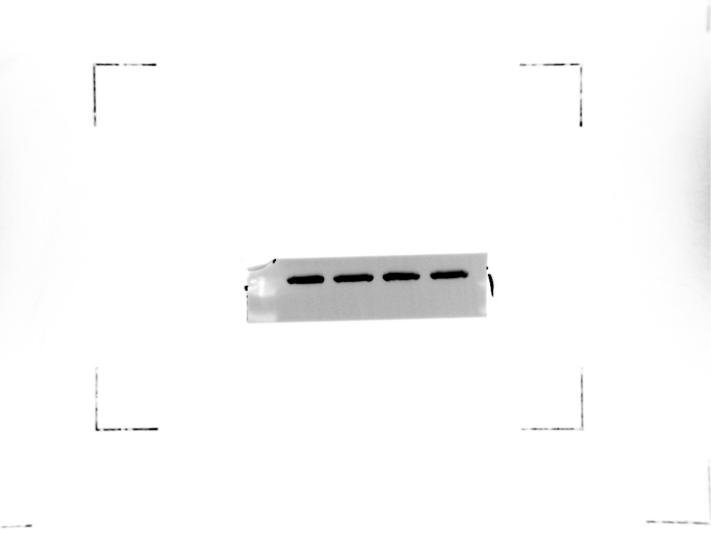


Figure S5:


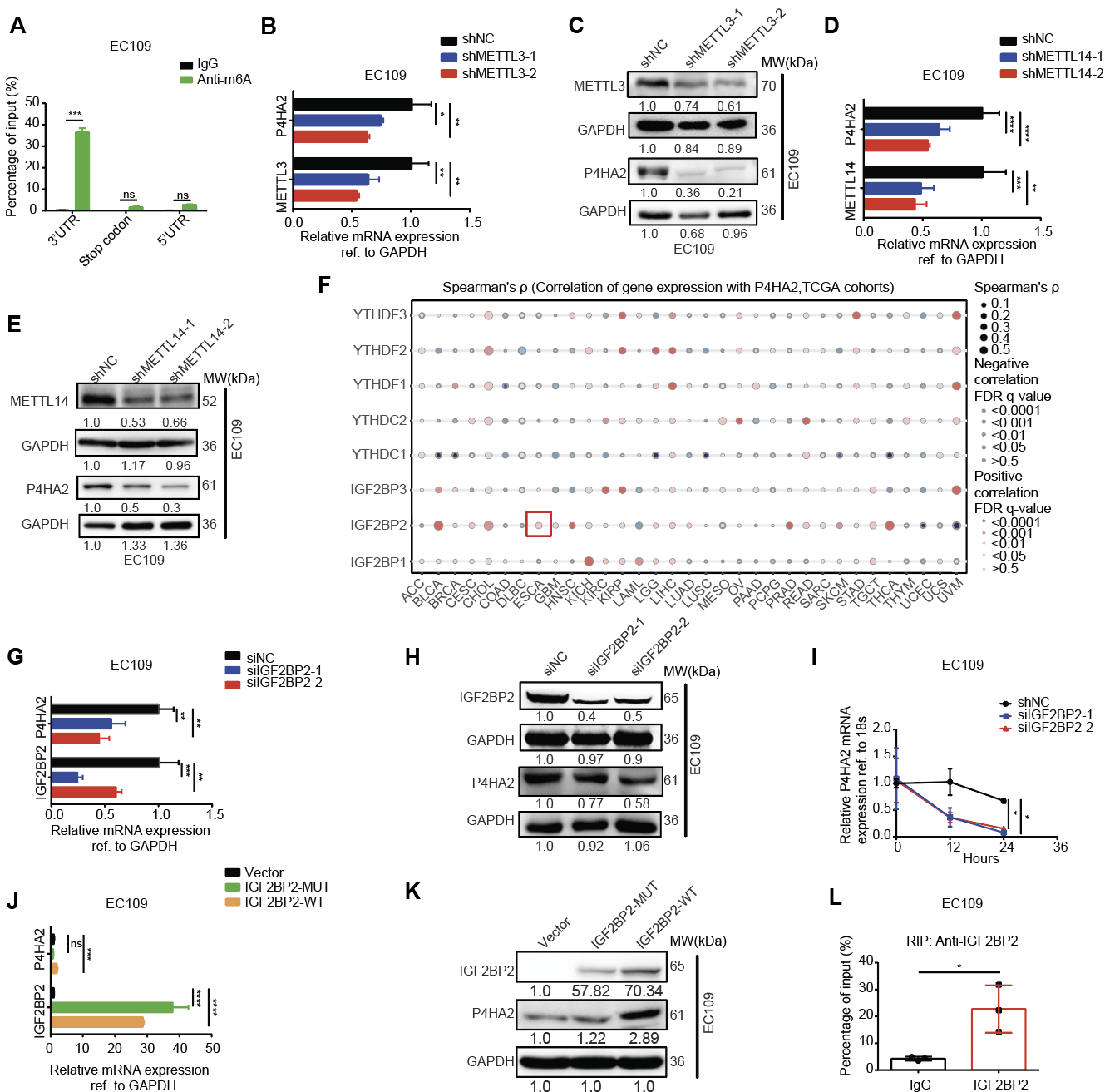


C:


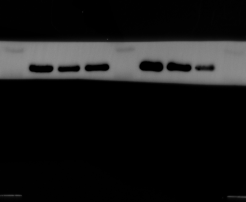

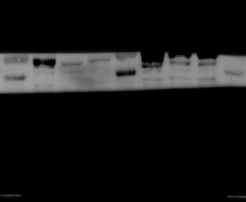

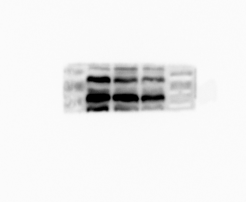

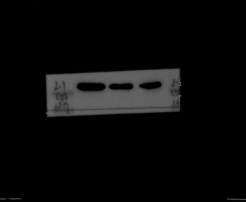


E:


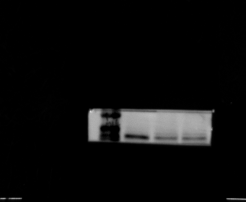

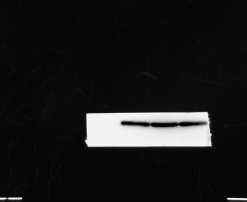

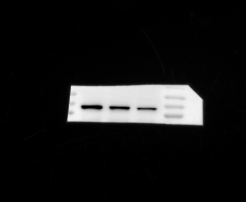

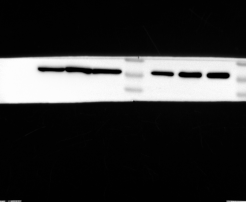


H：


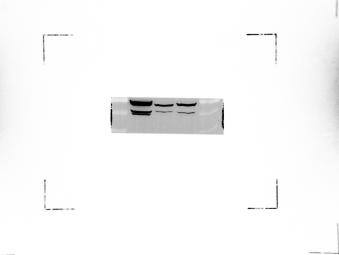

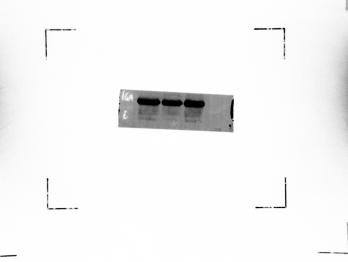

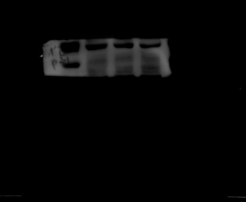

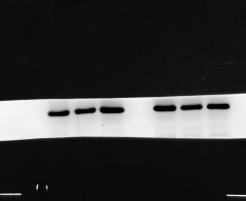


K:


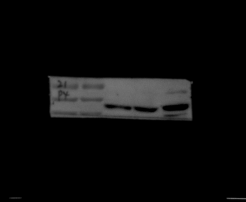

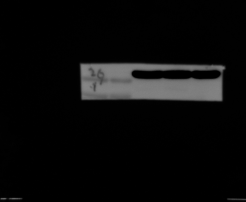

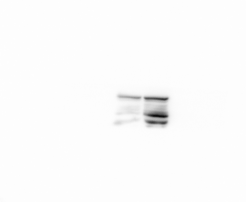

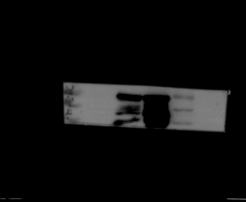

Supplement: Supplementary file 2 — Raw data for western blot of the supplementary materials [file 41419_2025_7864_MOESM2_ESM.docx]
