# Supplementary figures and images for: P4HA2 interacted with ATAD3A to modulate PINK1/parkin-dependent mitophagy and 125I brachytherapy sensitization in esophageal carcinoma
Source: Cell Death Dis. 2025 Oct 6;16(1):685. doi: 10.1038/s41419-025-07864-x (PMC12501296; doi:10.1038/s41419-025-07864-x)

**Raw data of western blot**

Figure 1:


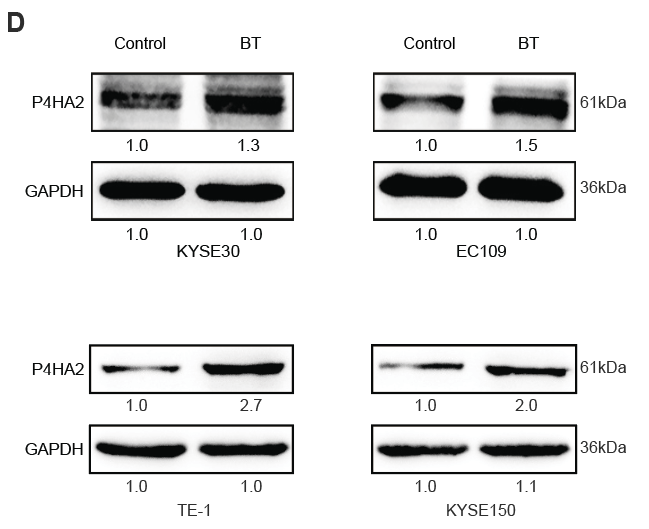


D:


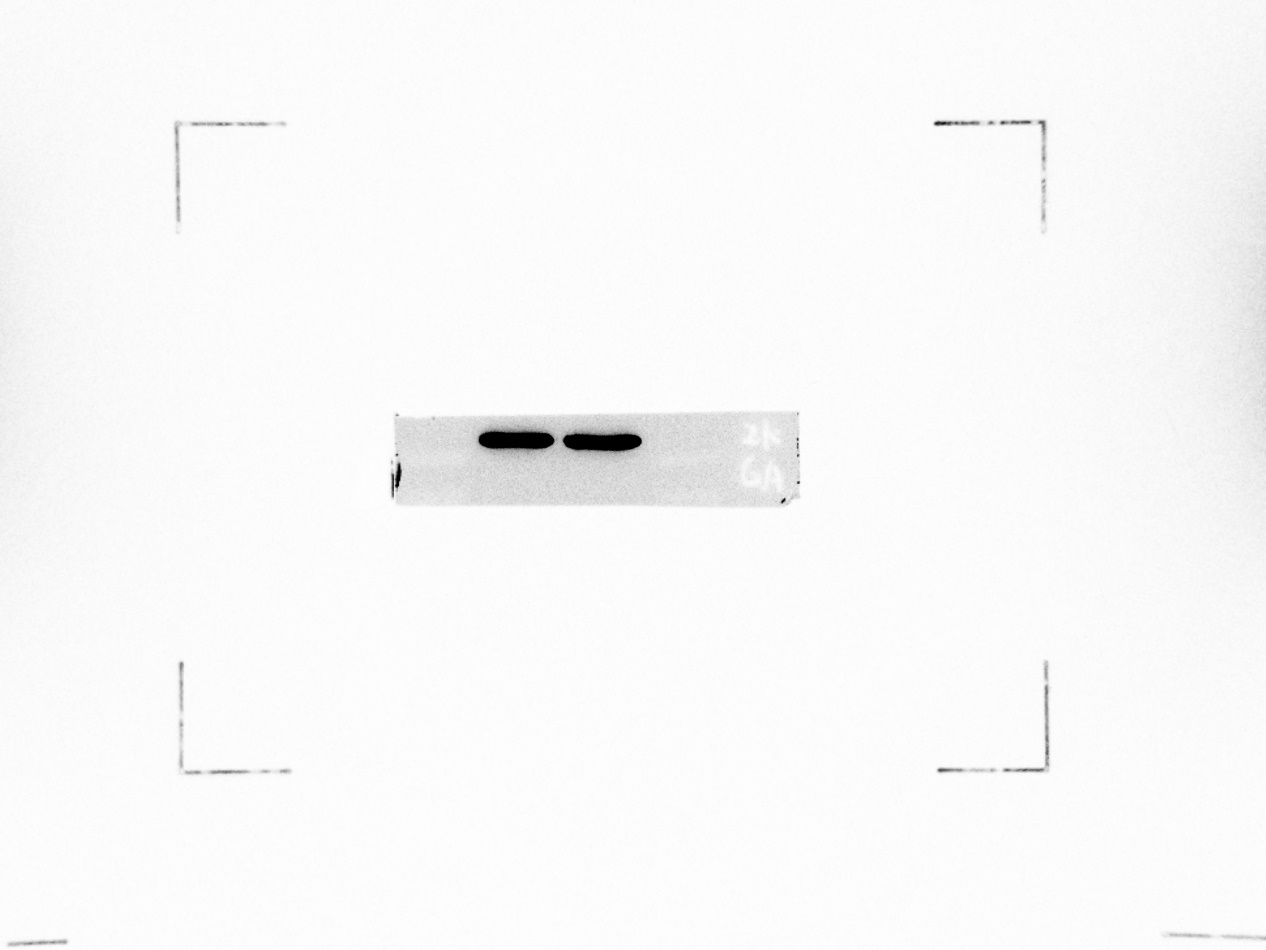

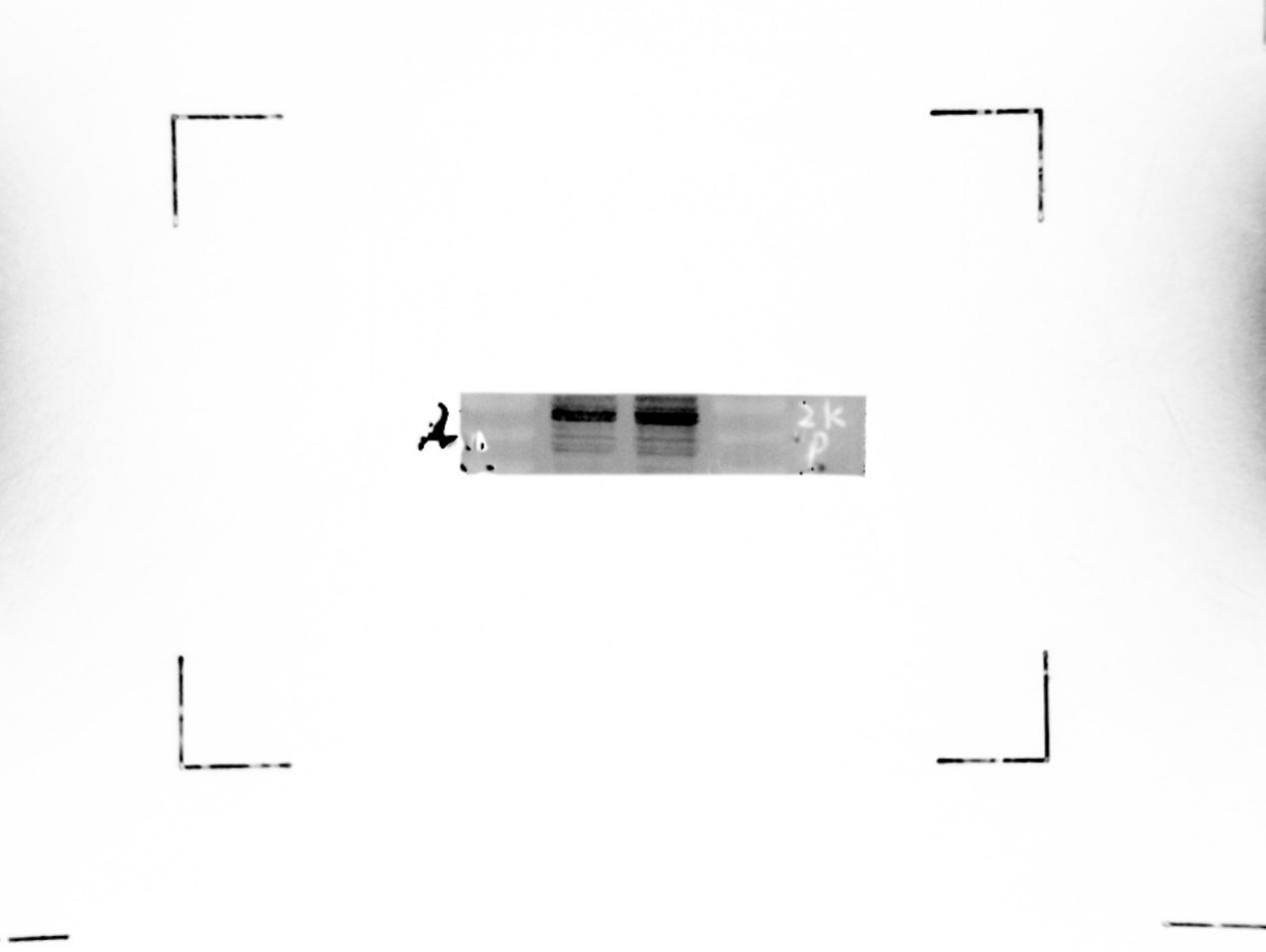


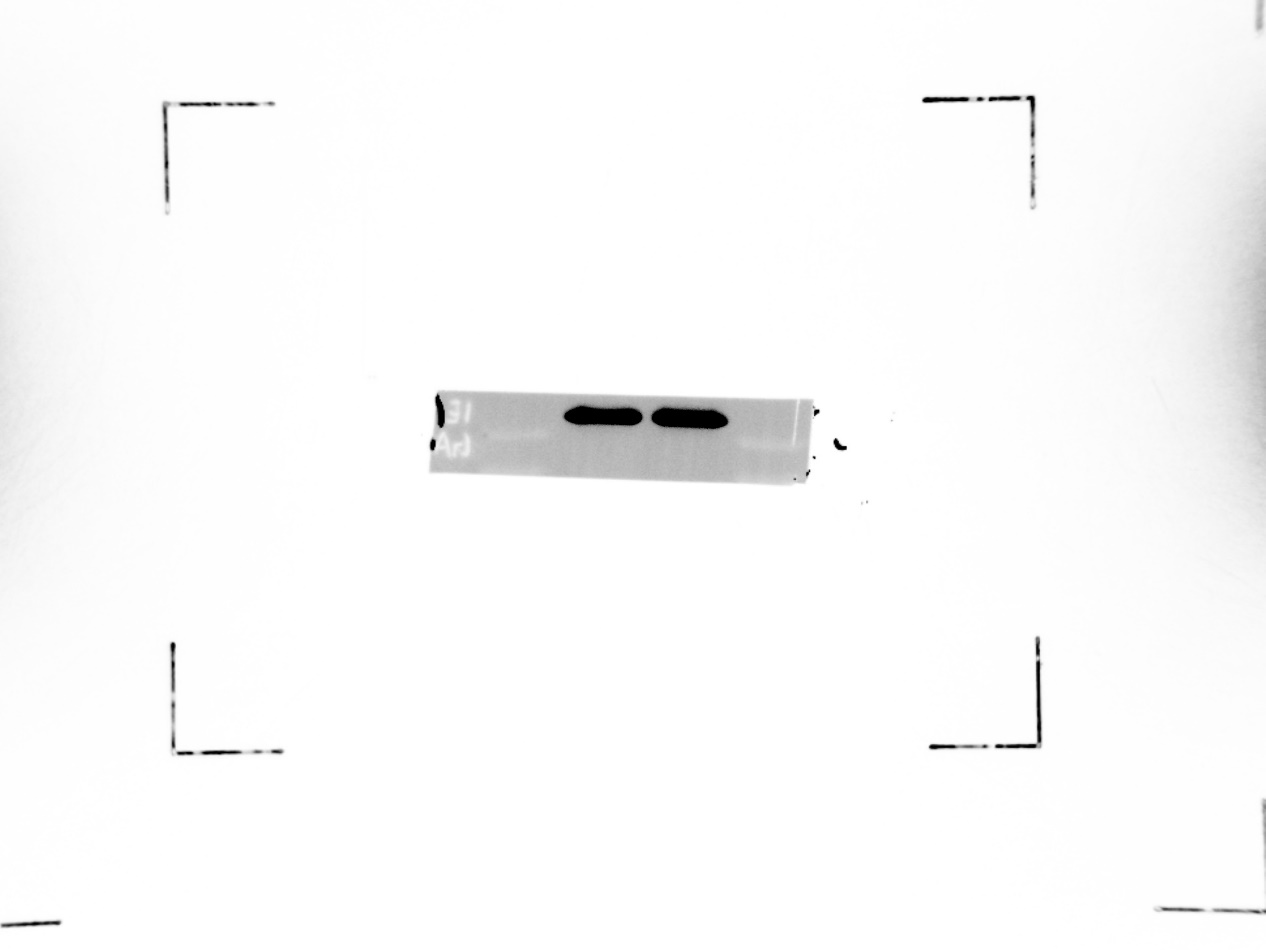

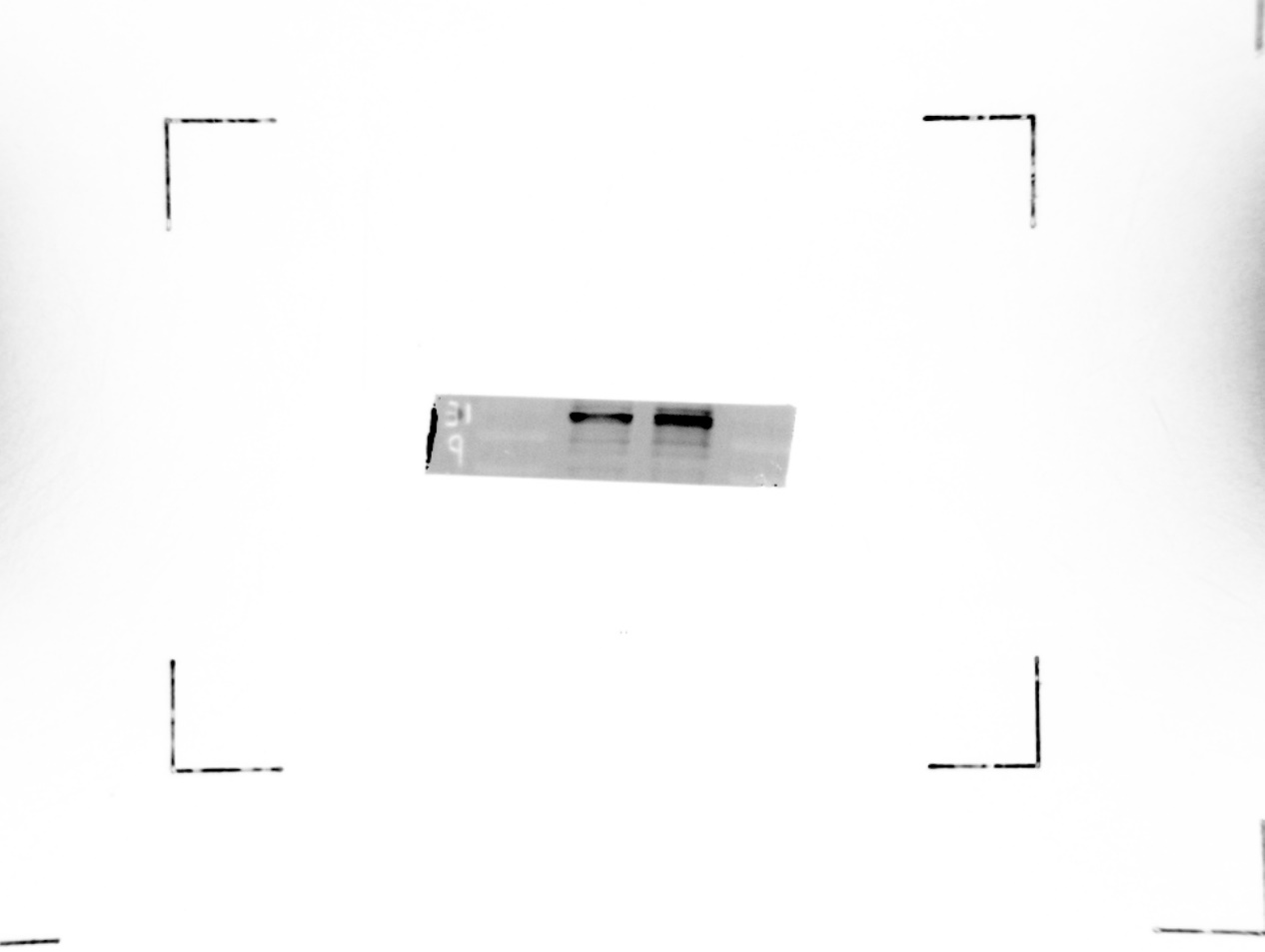


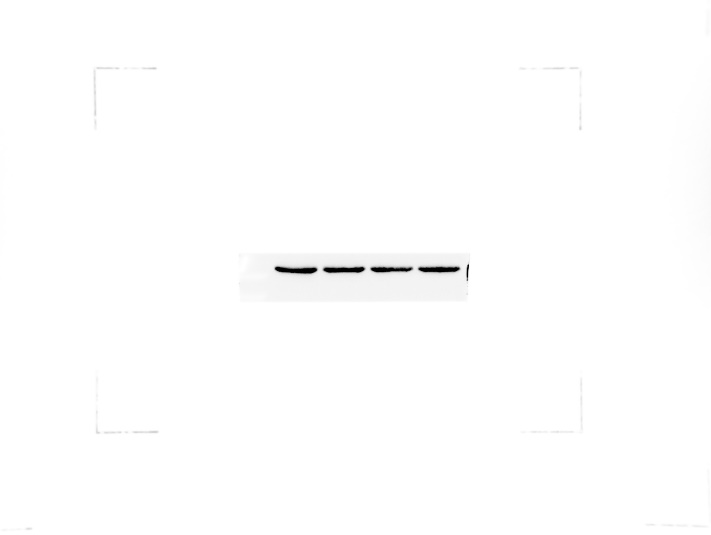

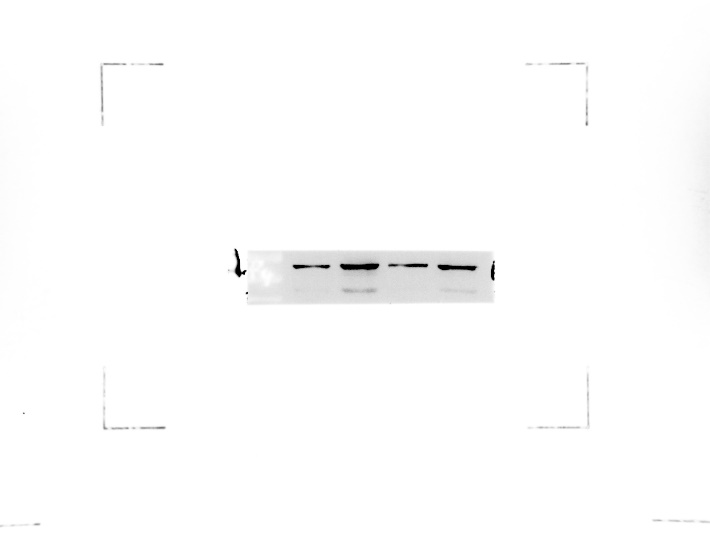


Figure 2:


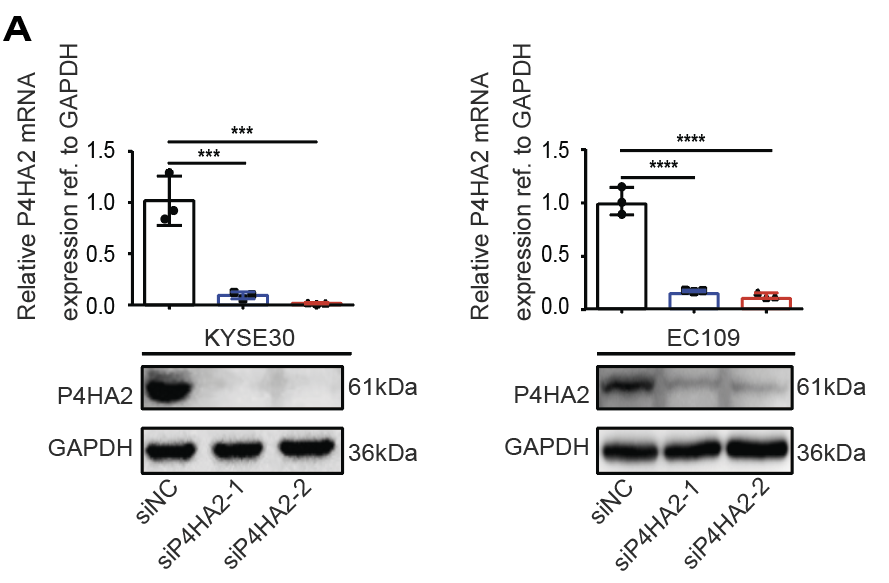


A:


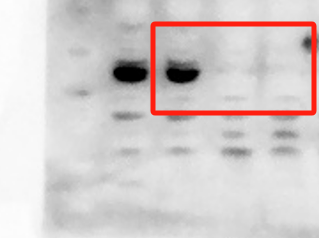

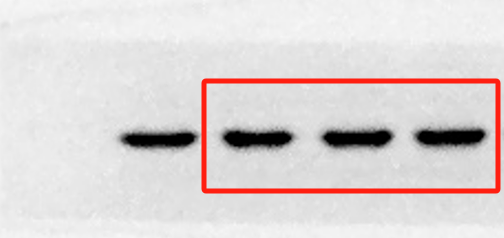


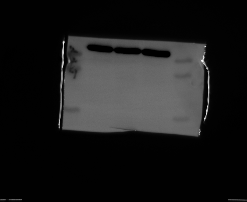

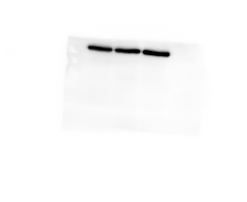

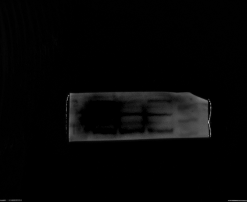

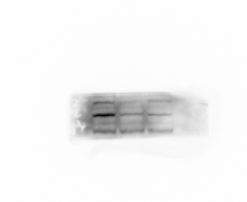


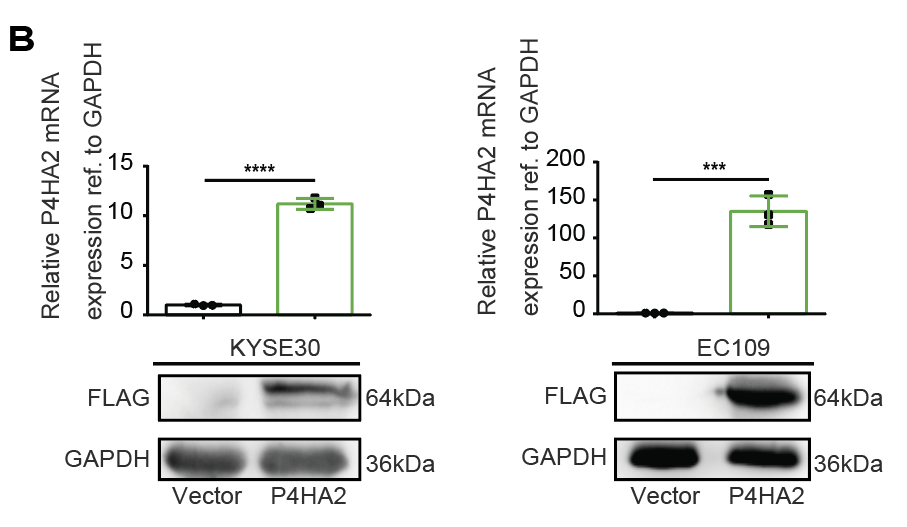


B:


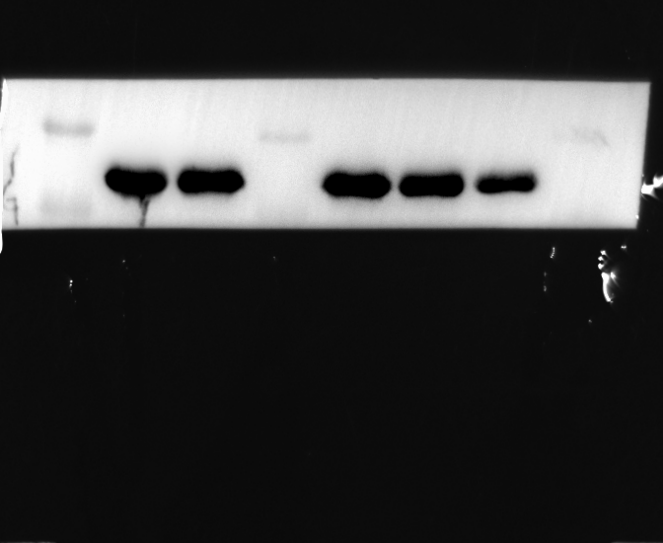

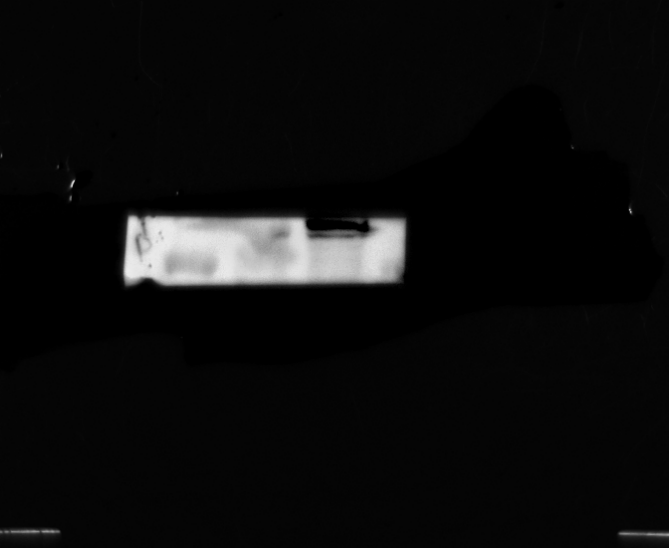


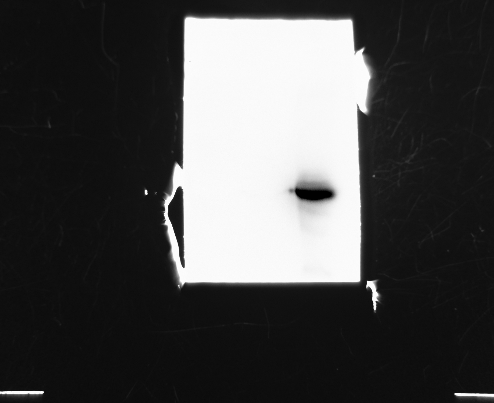

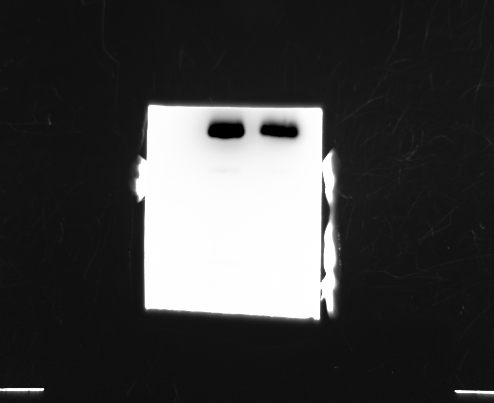


Figure 3F:


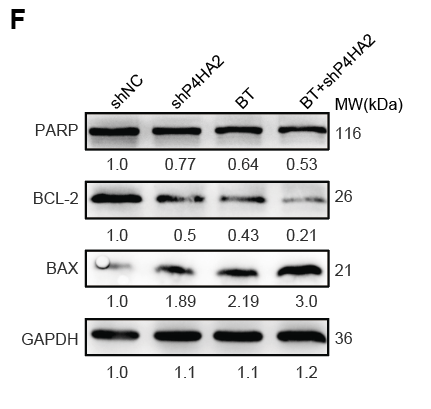


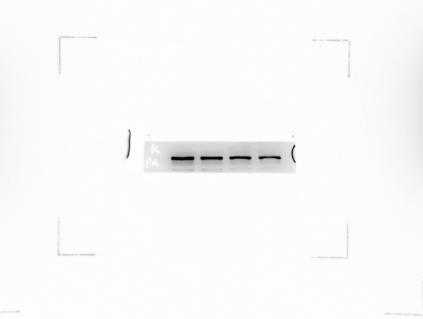

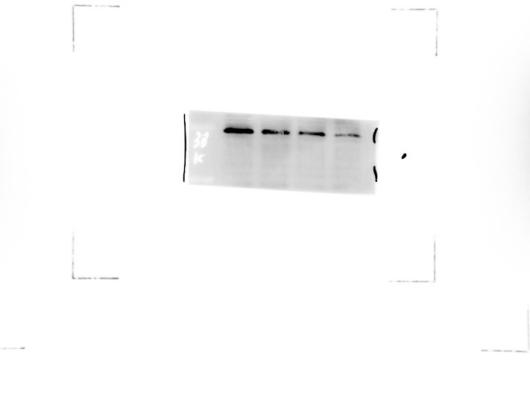

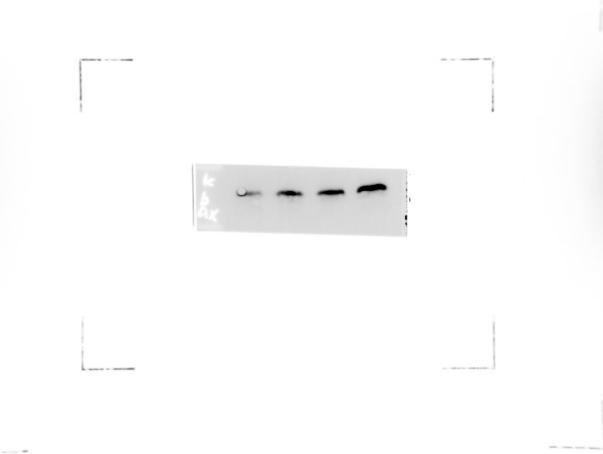

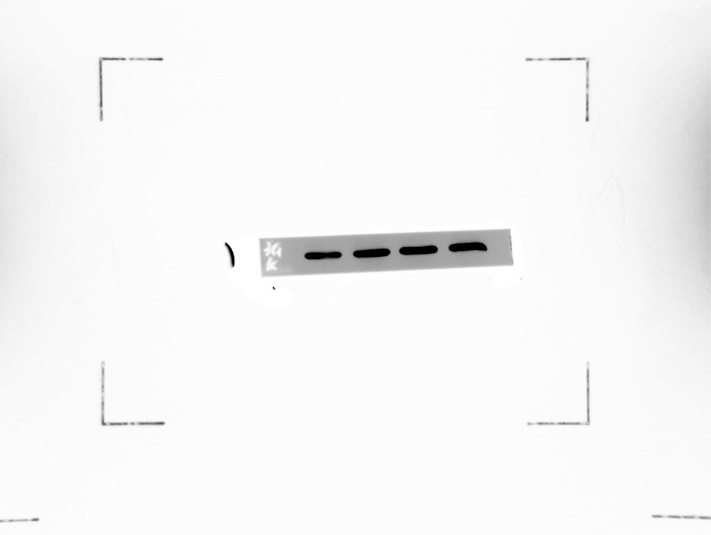


Figure 4:


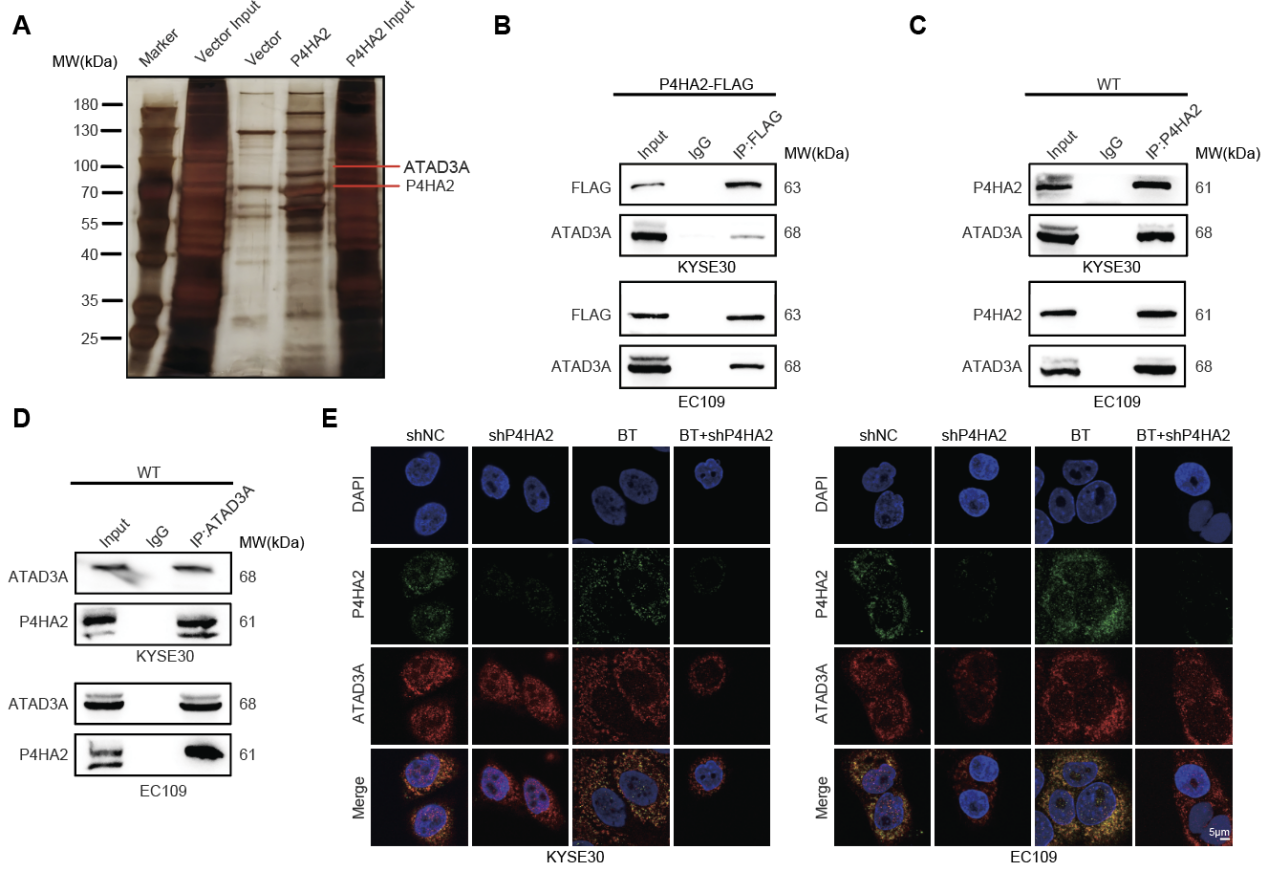


B:


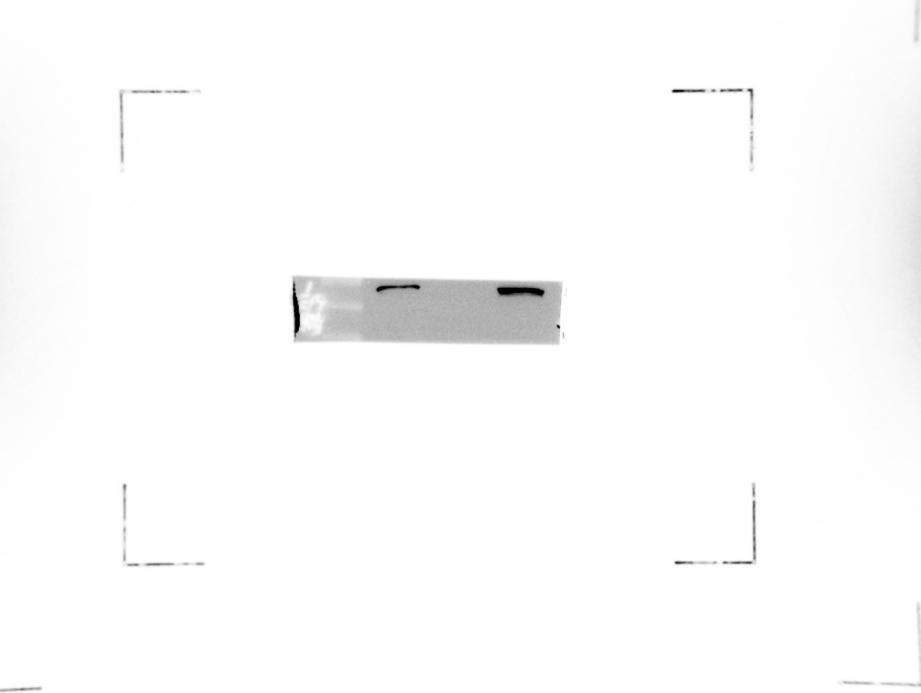

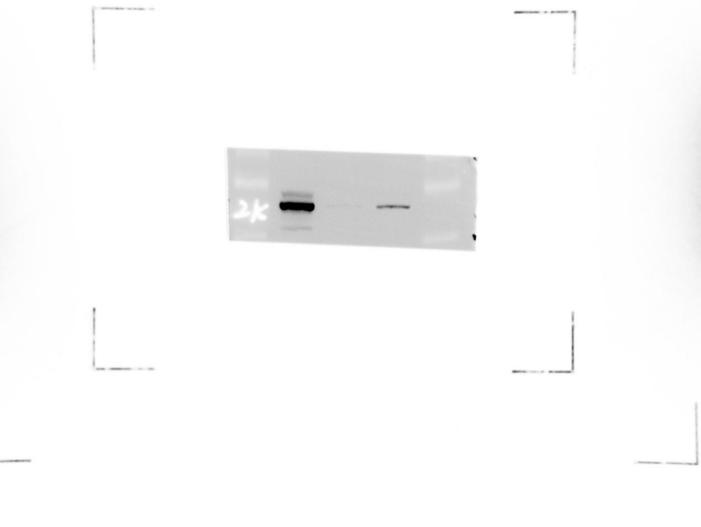


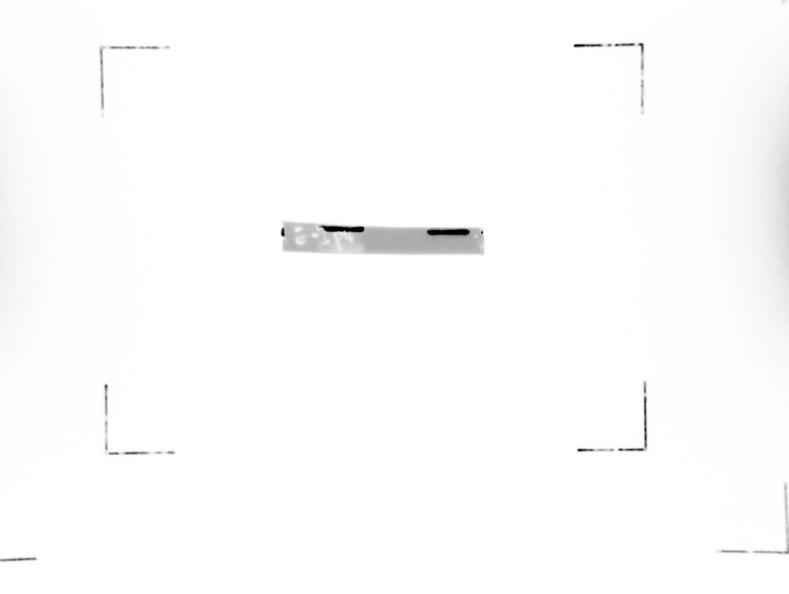

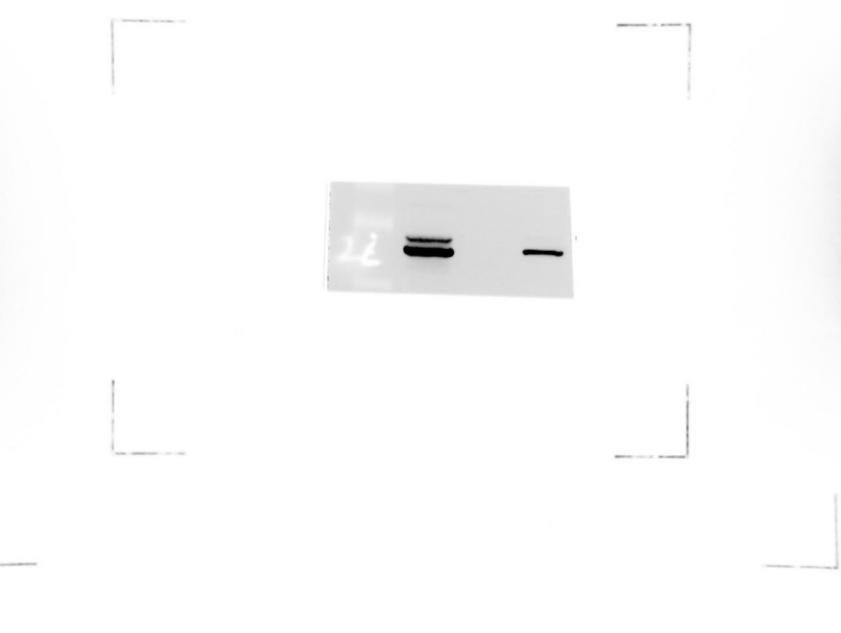


C:


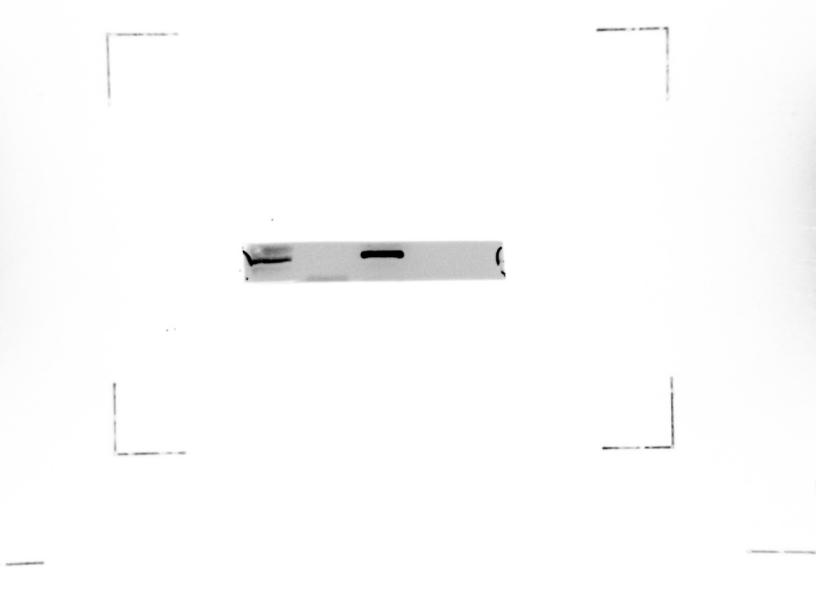

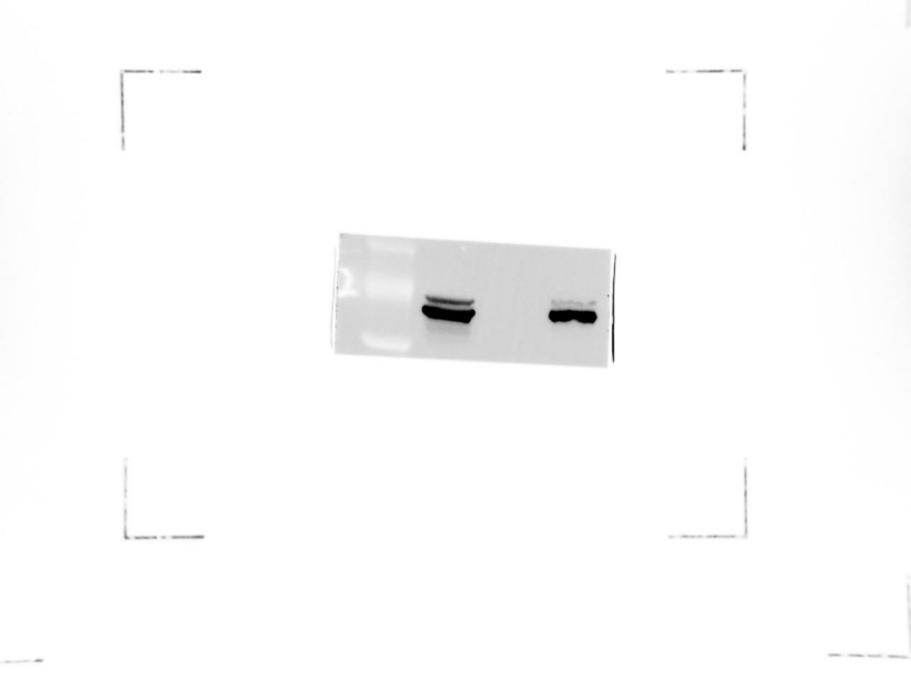


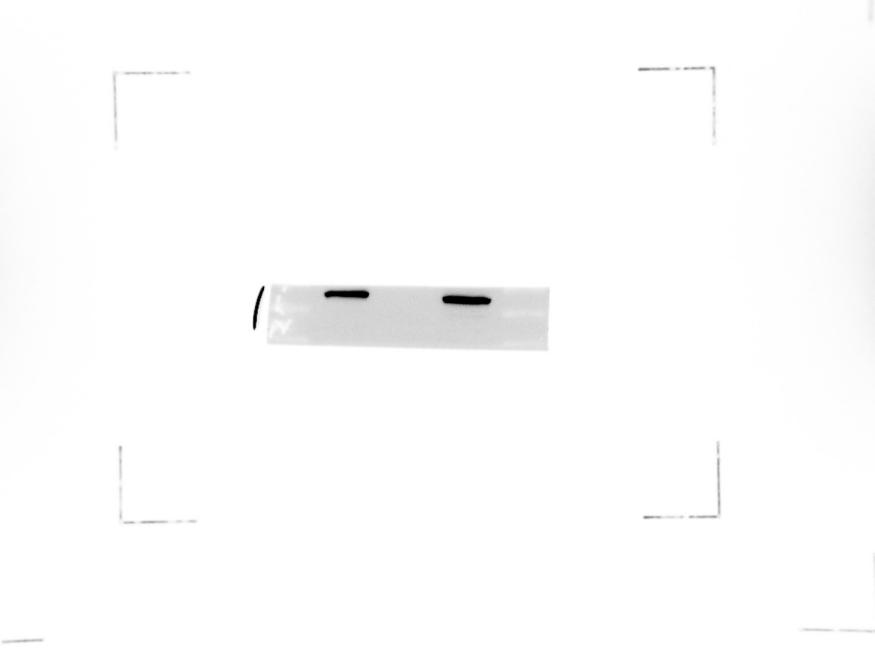

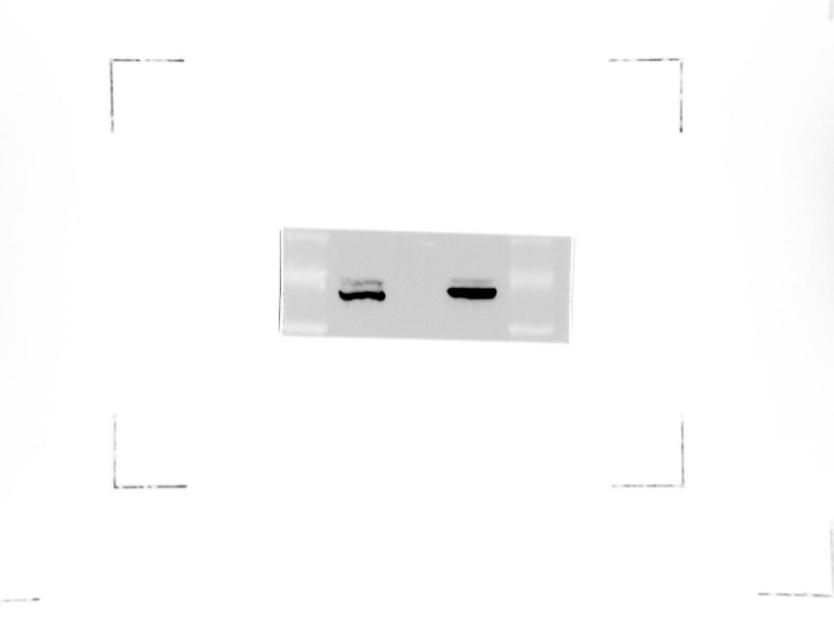


D:


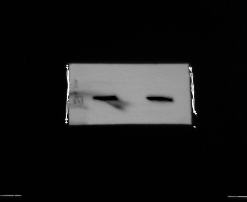

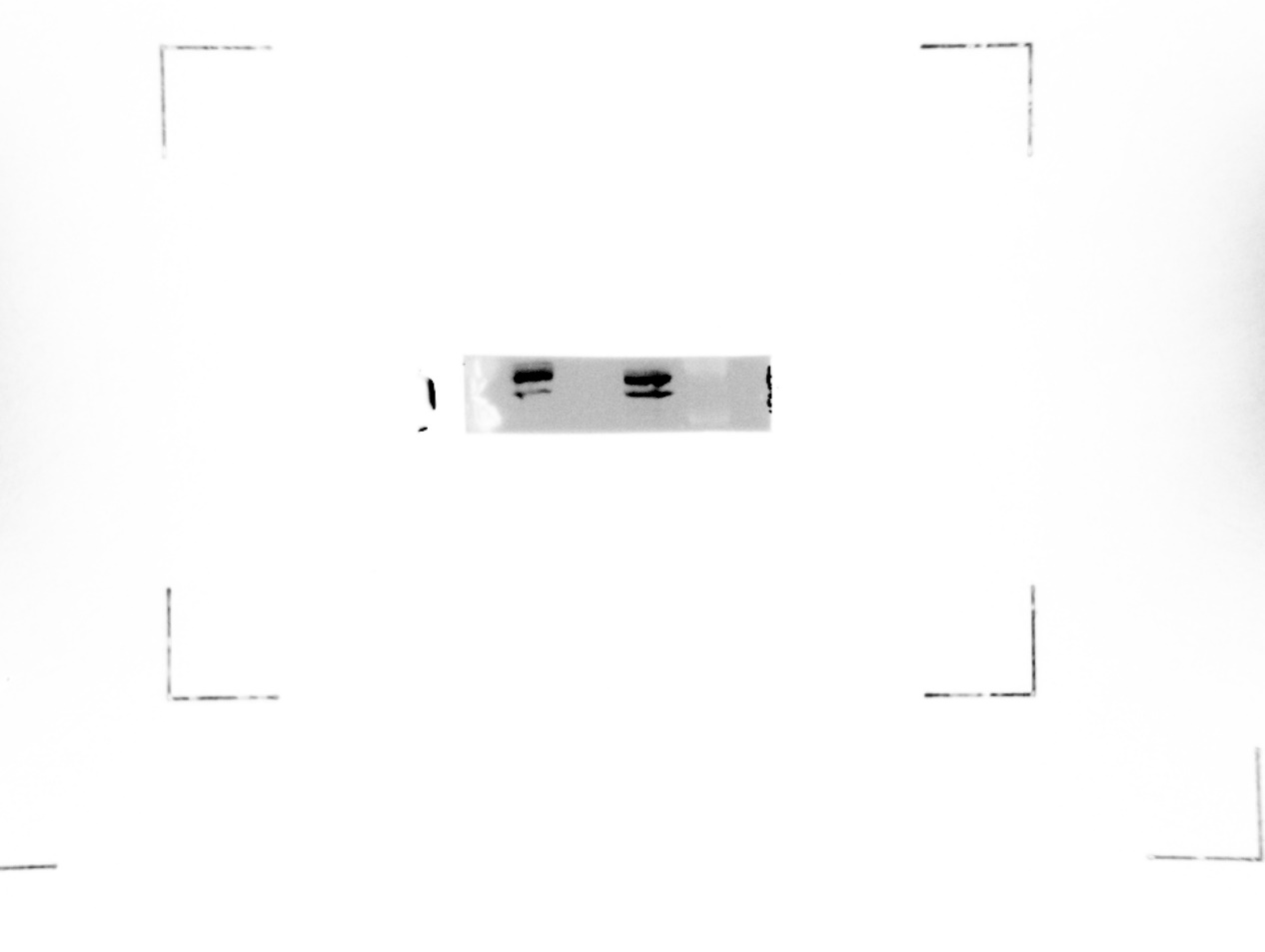


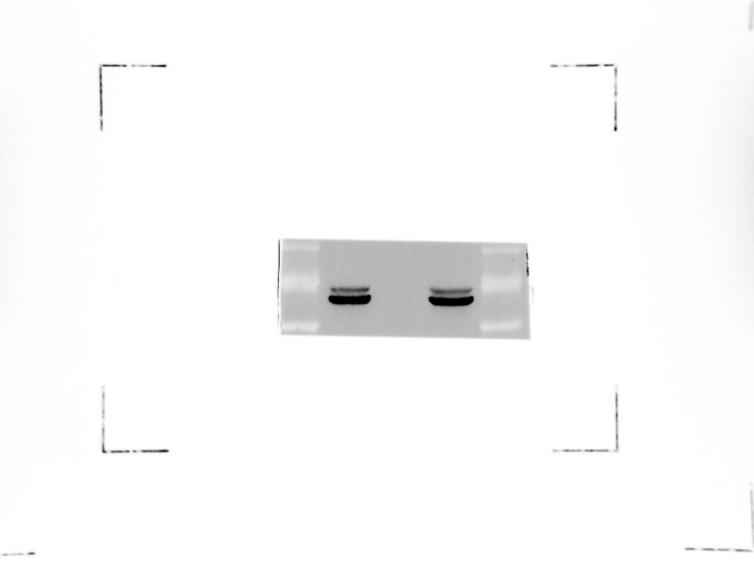

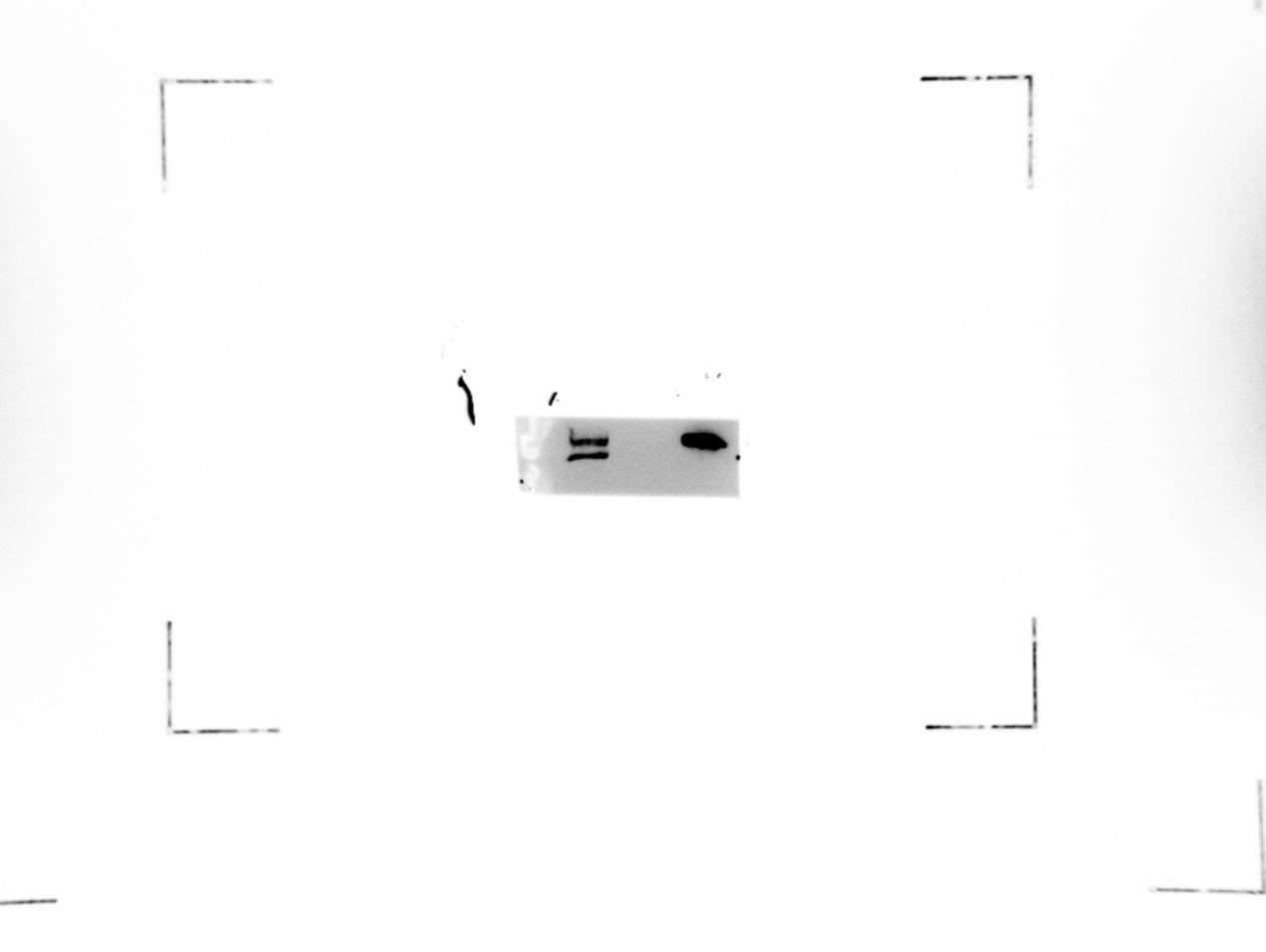


Figure 4


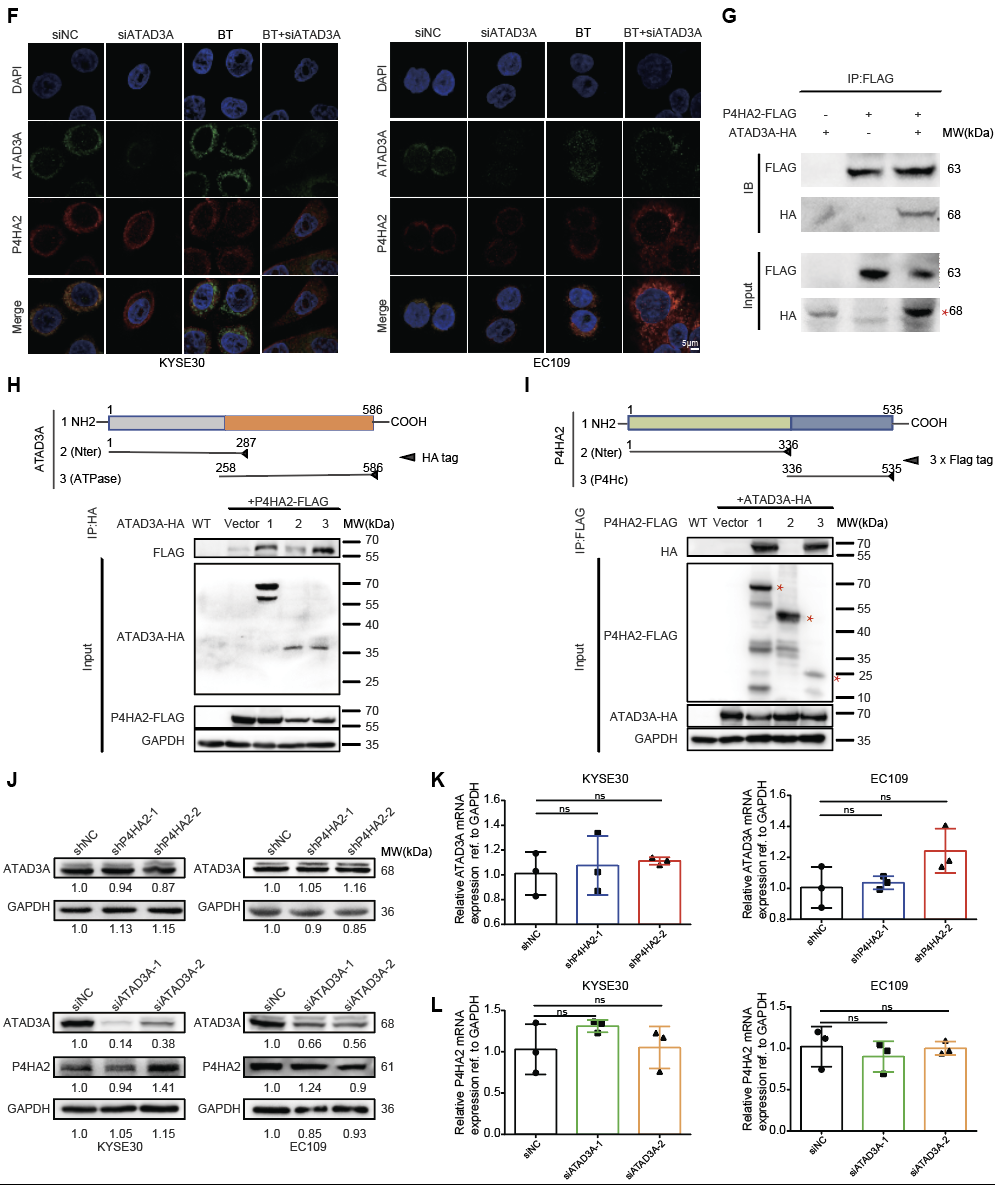


G:


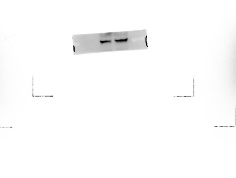

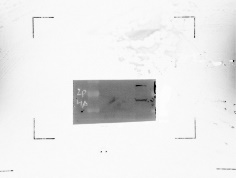

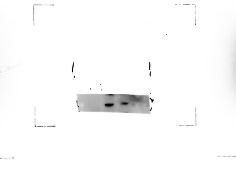

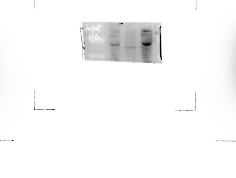


J:


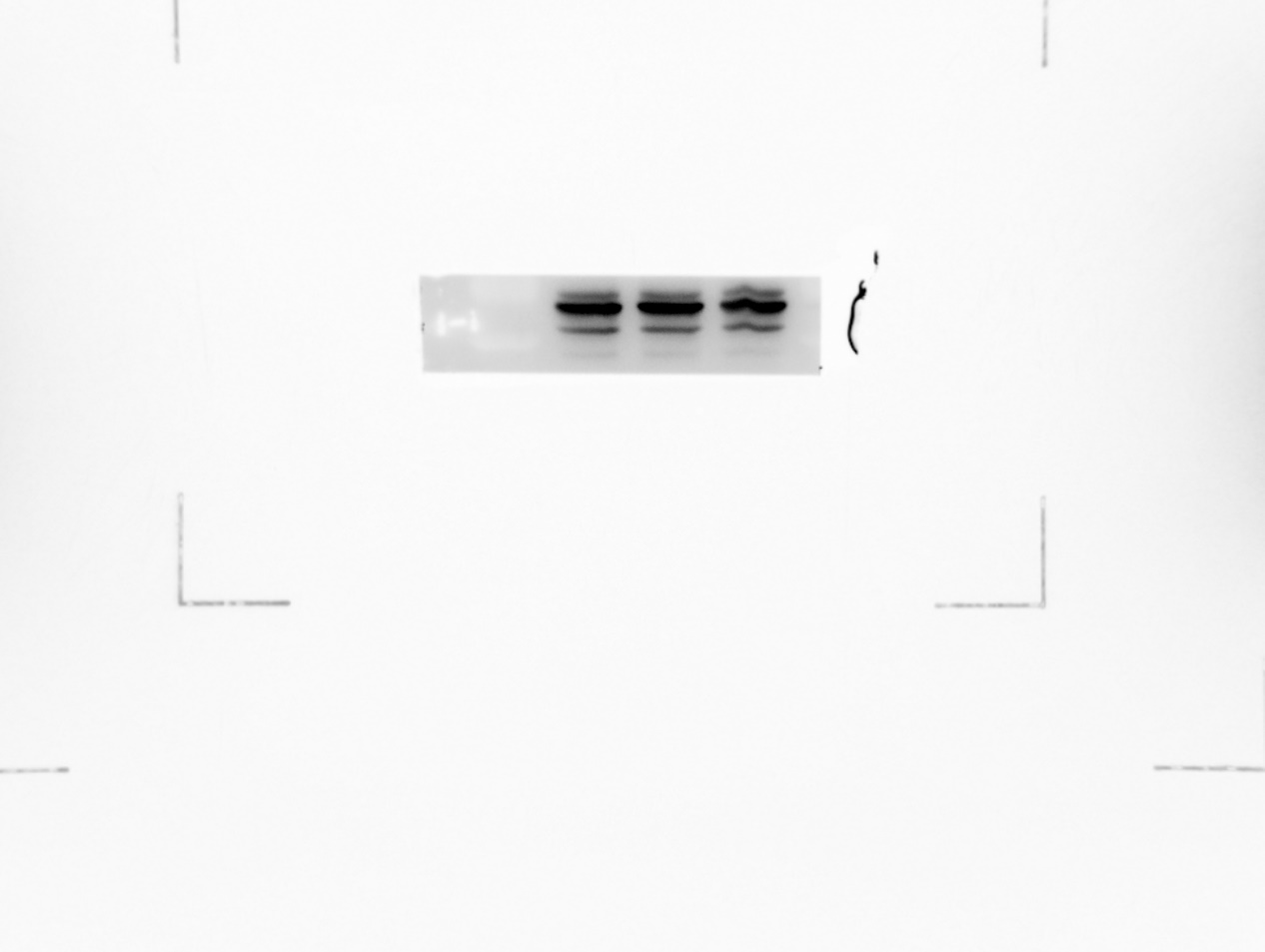

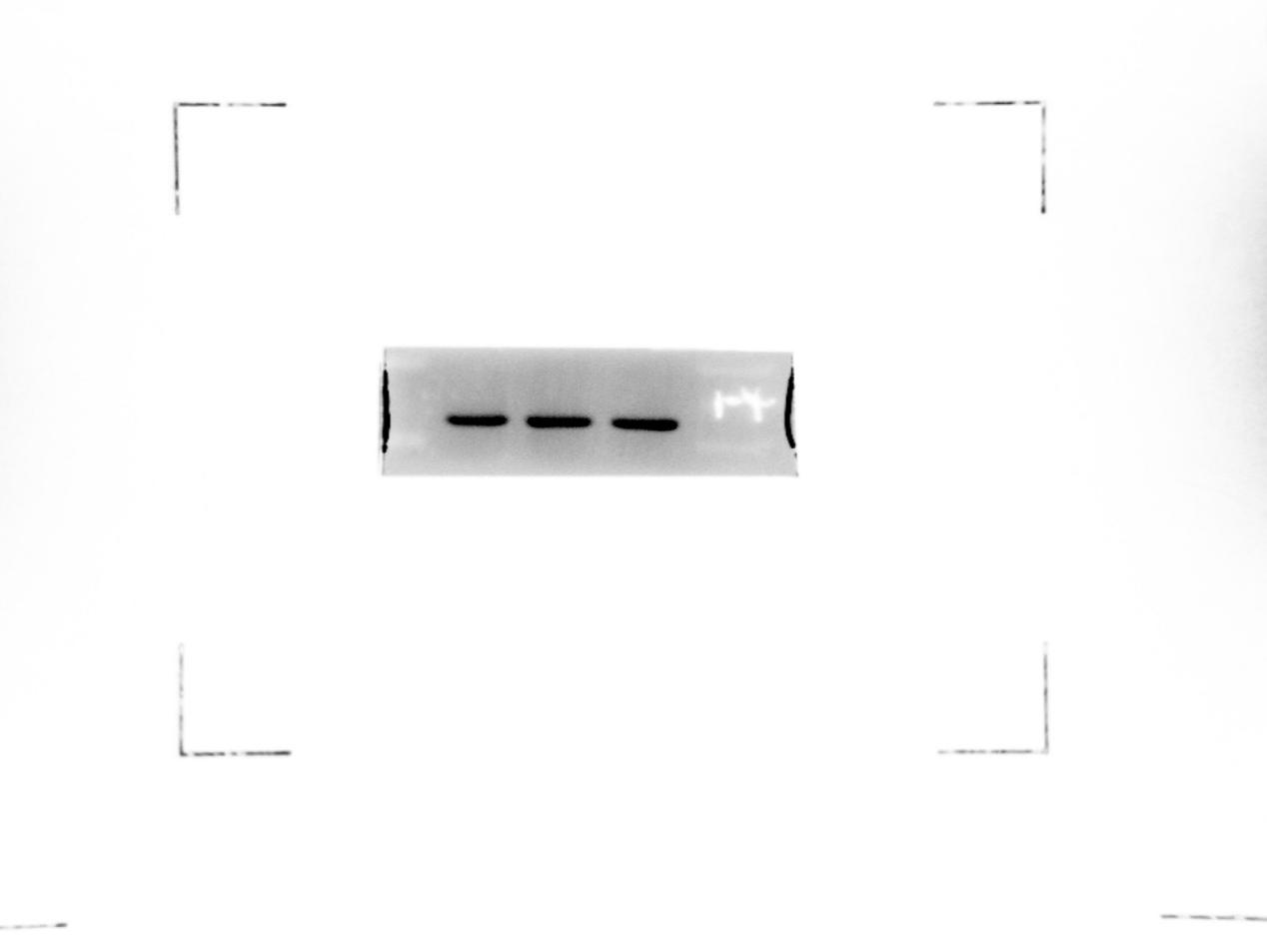


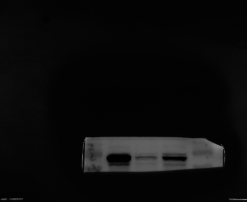

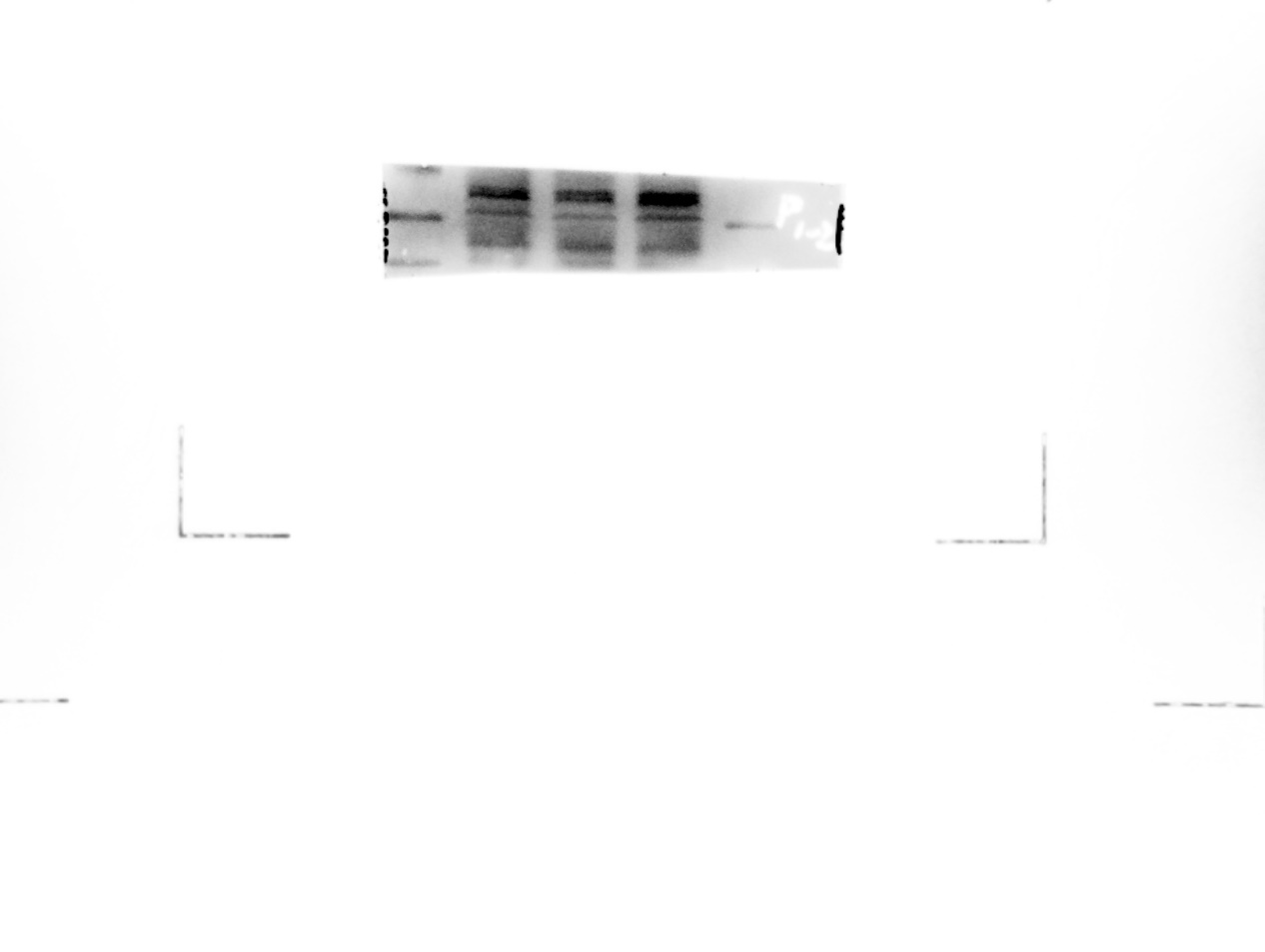

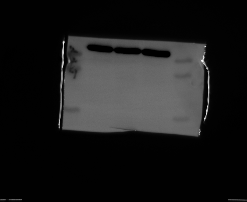


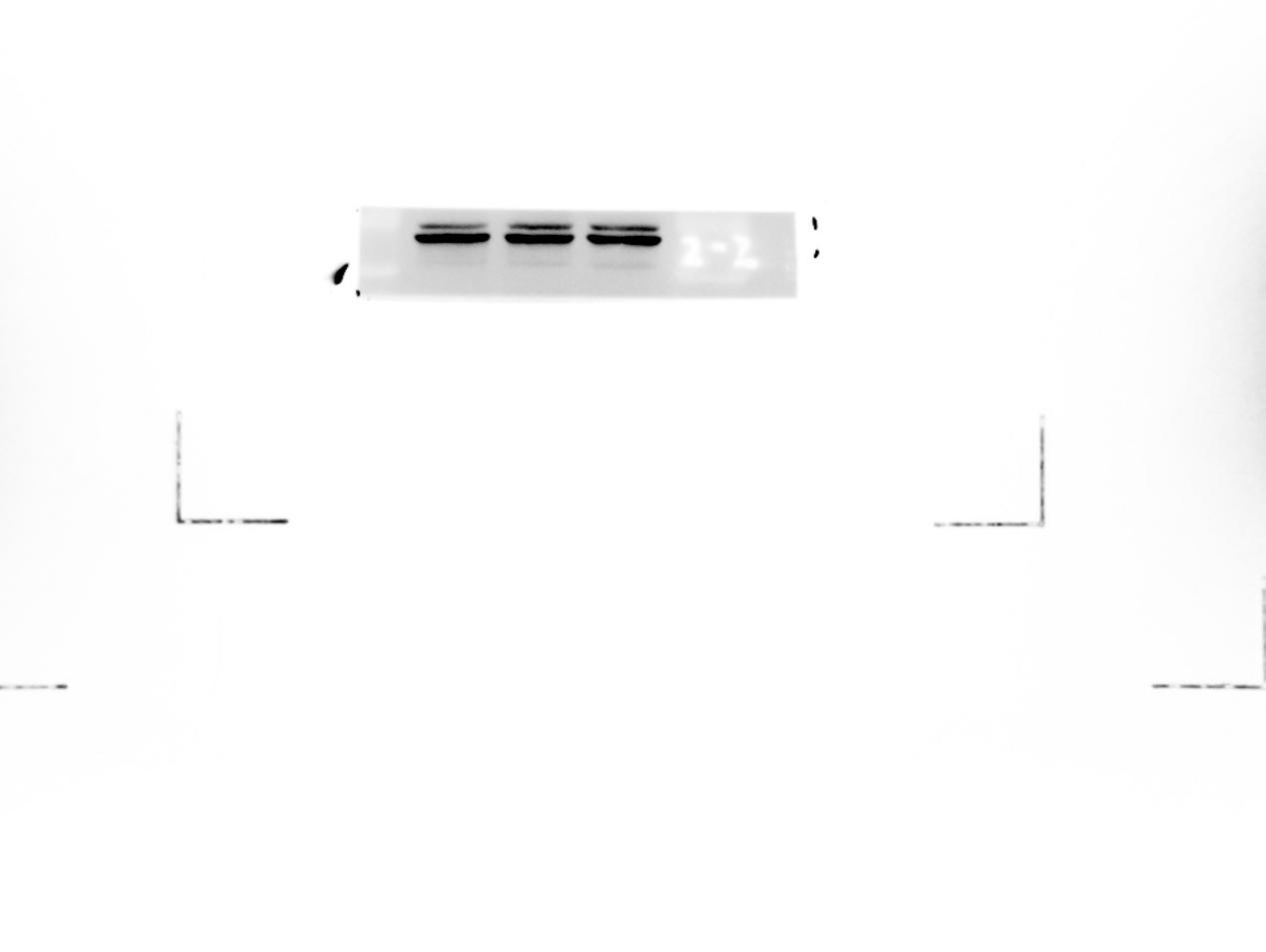

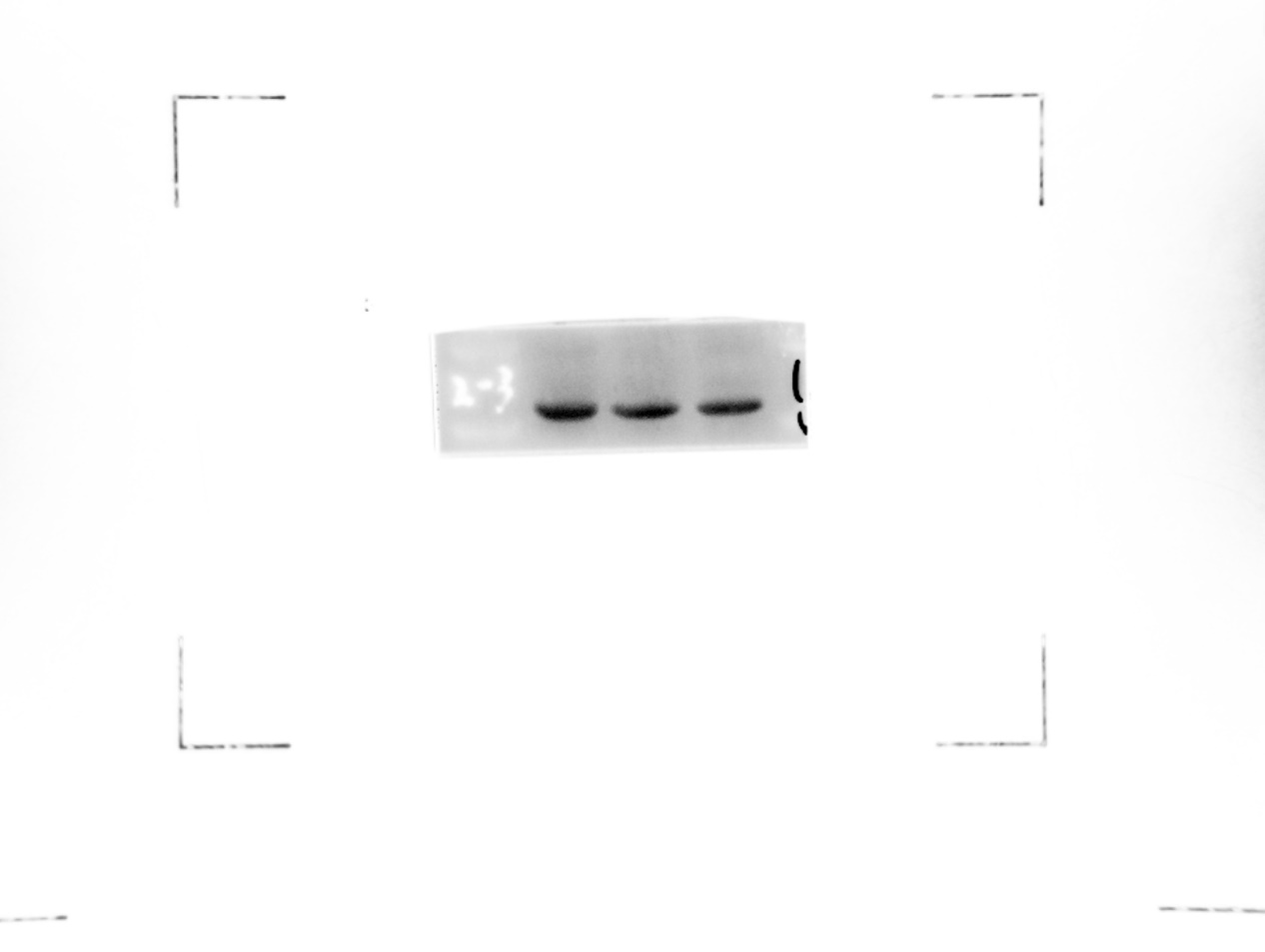


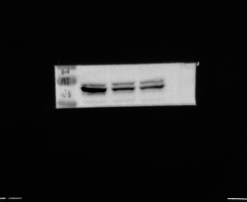

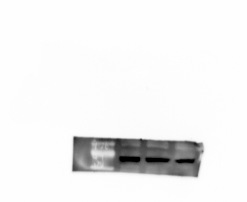

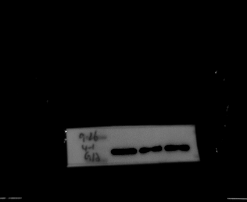


Figure 5


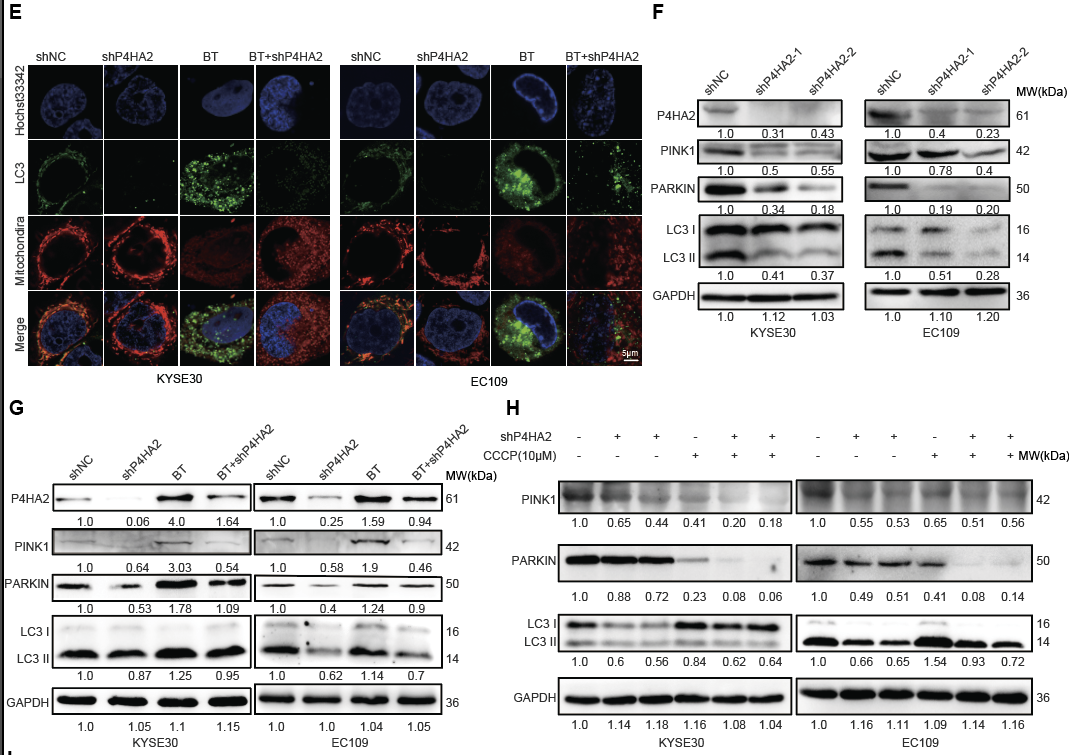


F:


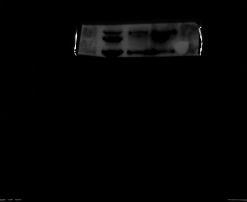

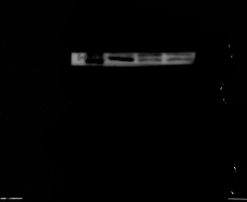

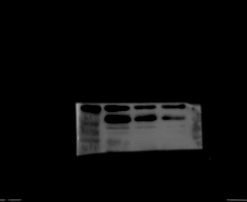

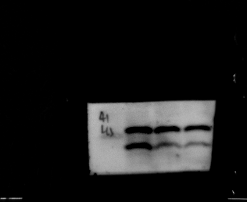

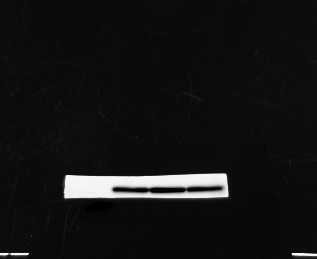

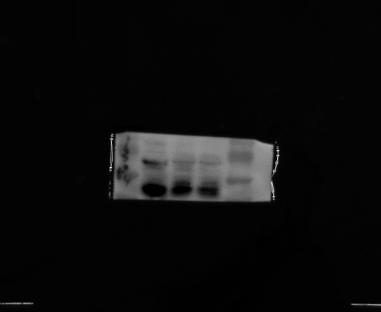

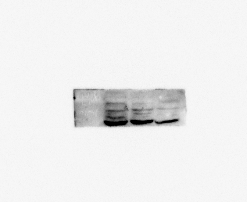

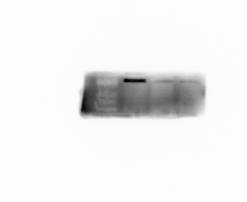

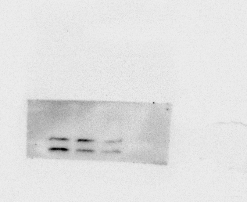

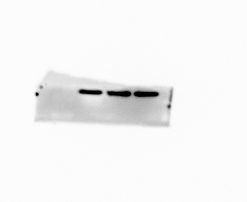


G:


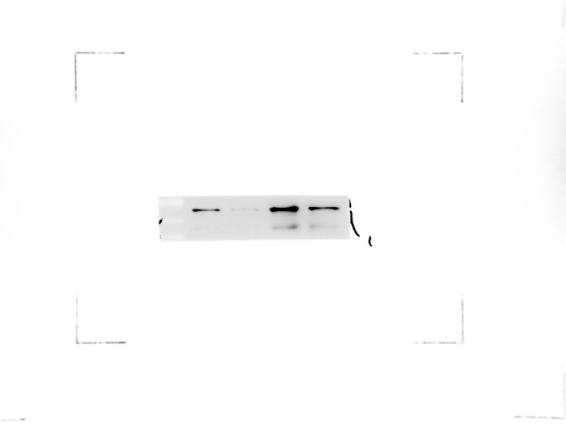

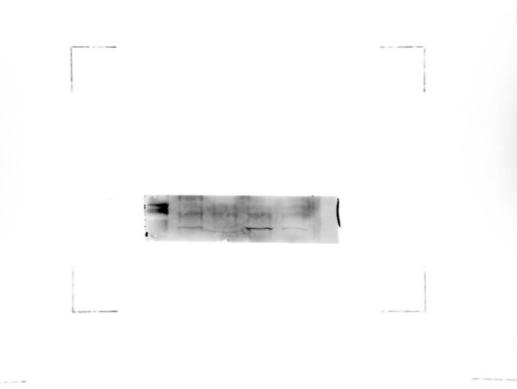

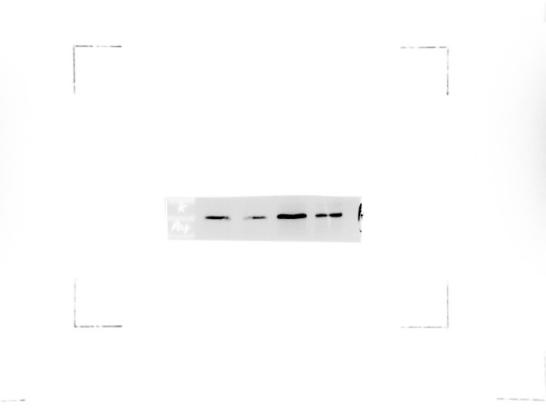

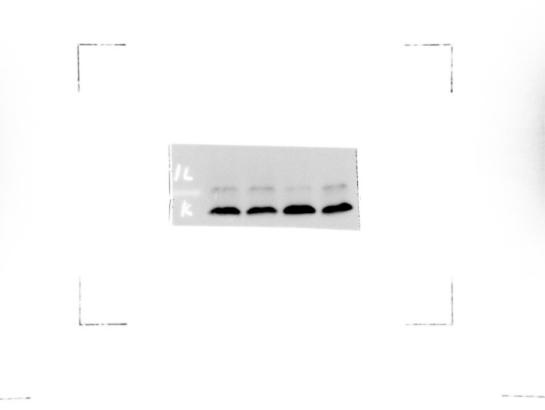

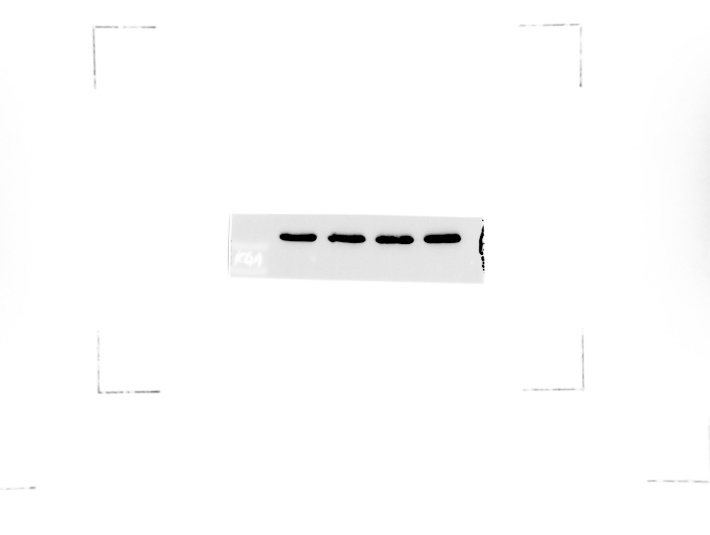

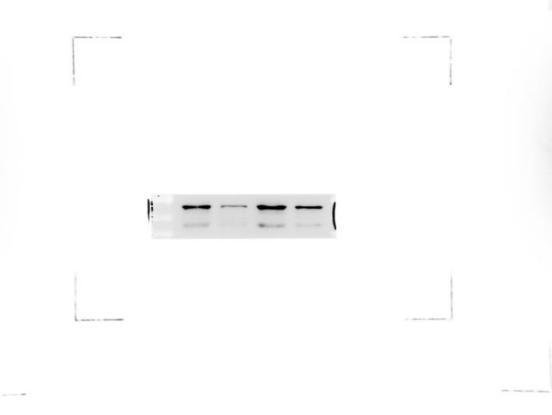

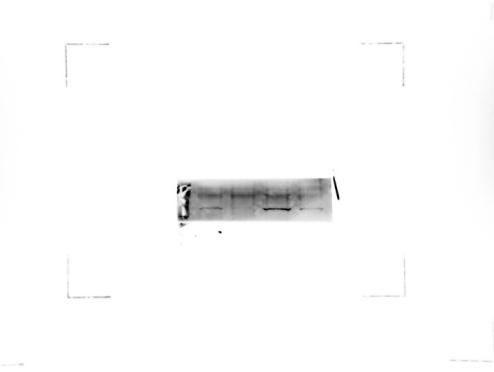

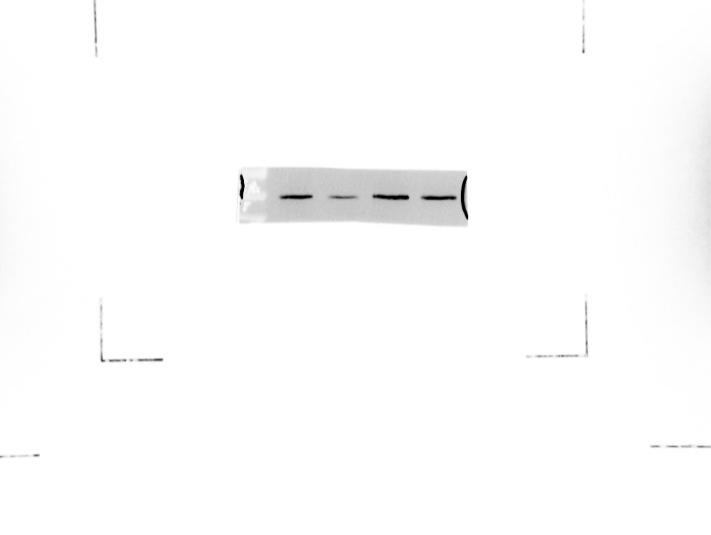

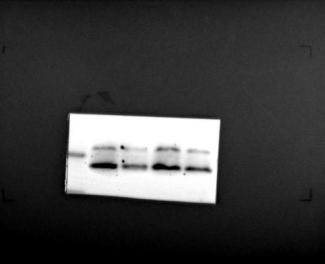

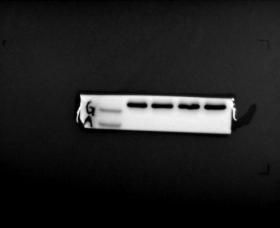


H:


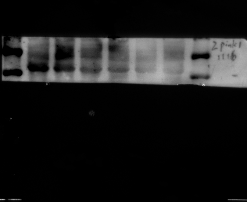

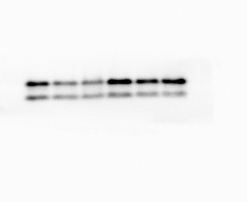

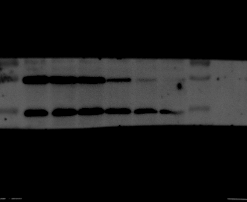

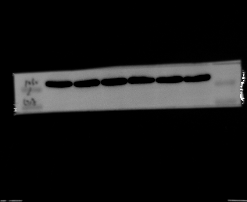

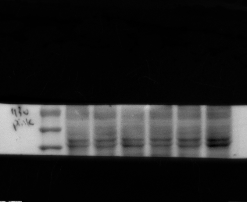

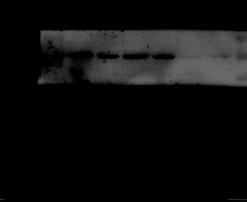

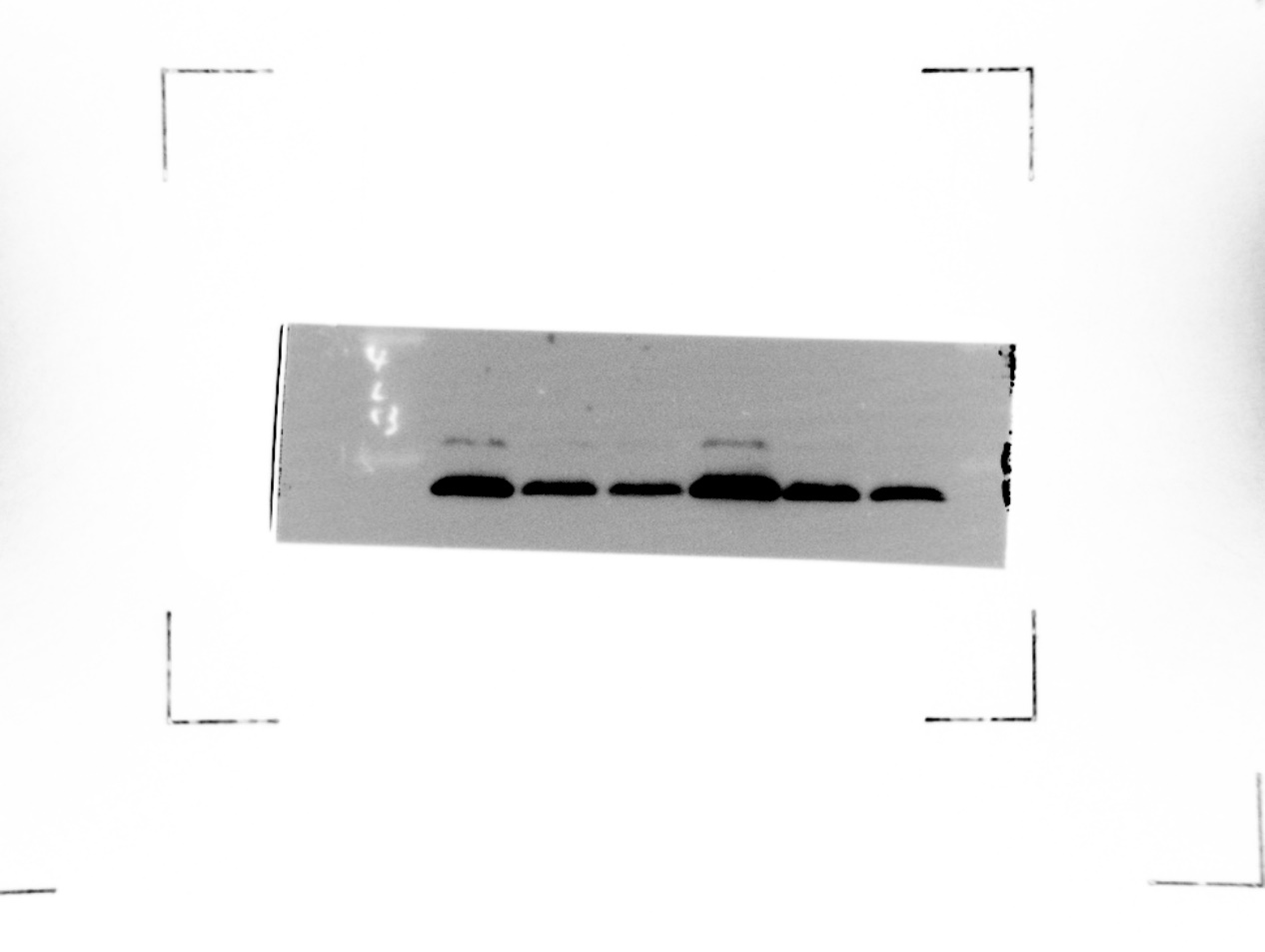

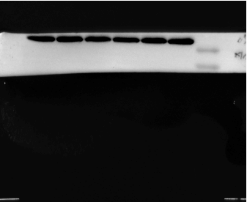


Figure 6:


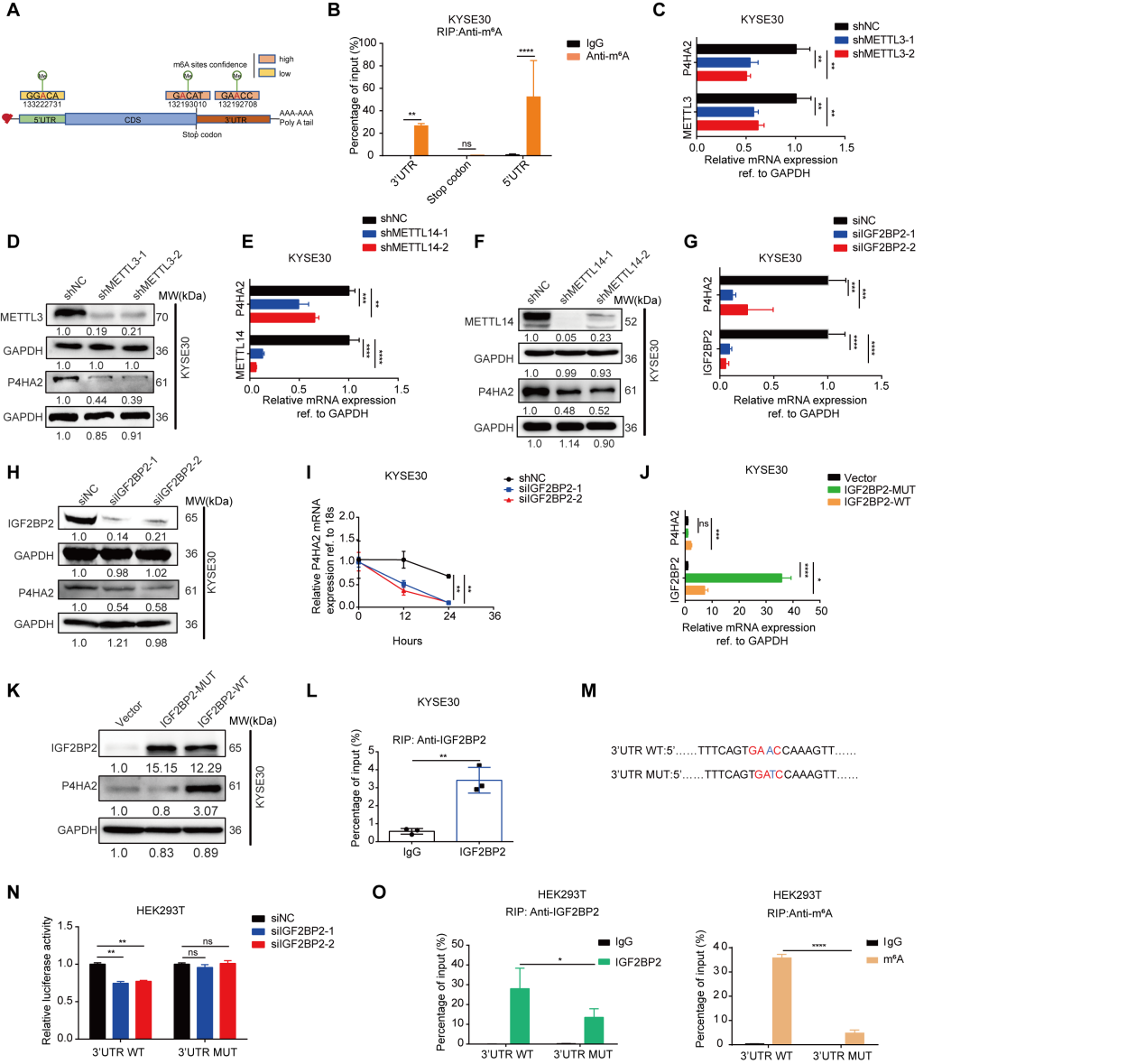


D:


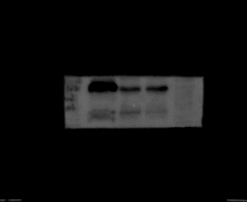

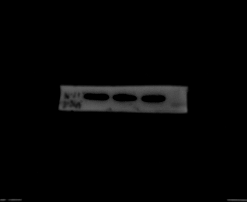

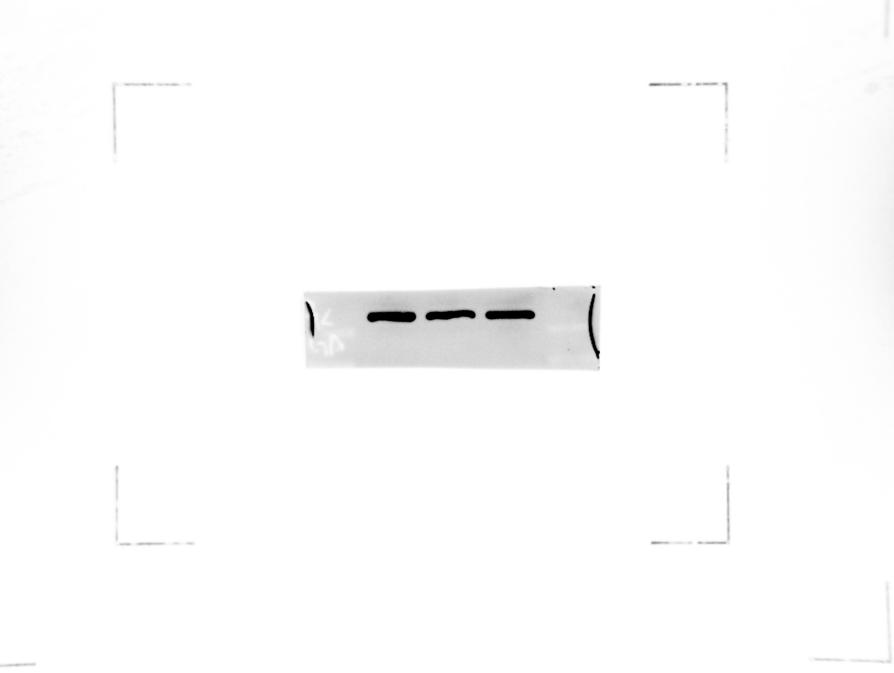

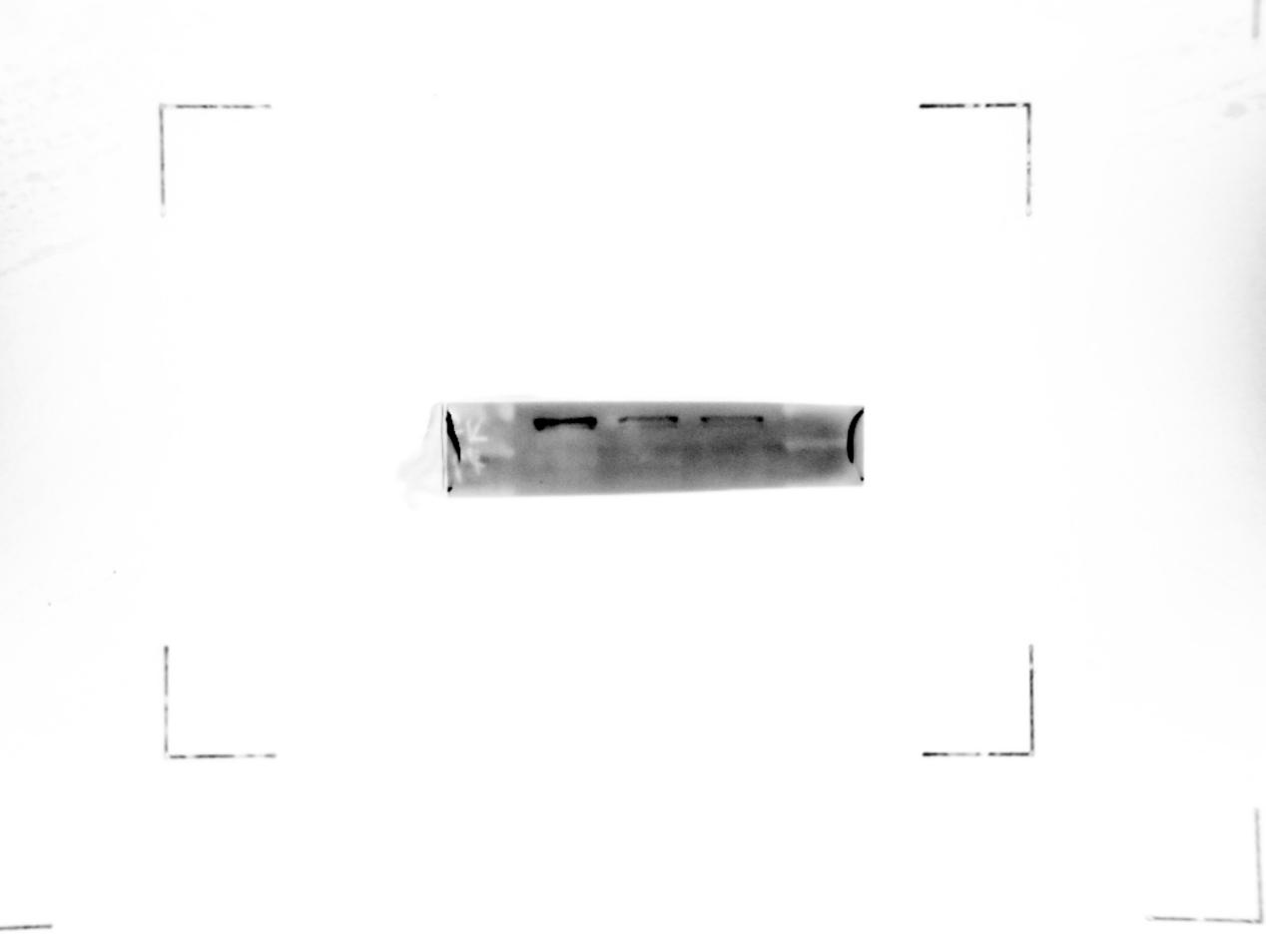


F:


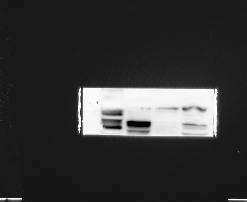

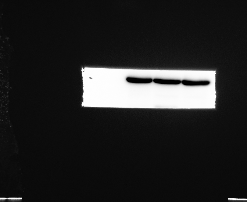

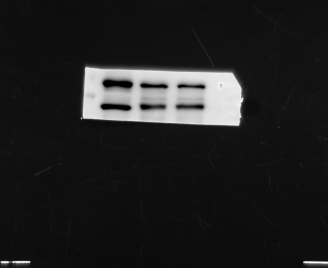

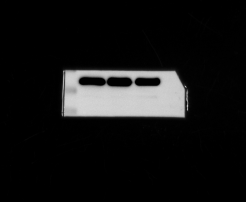


H:


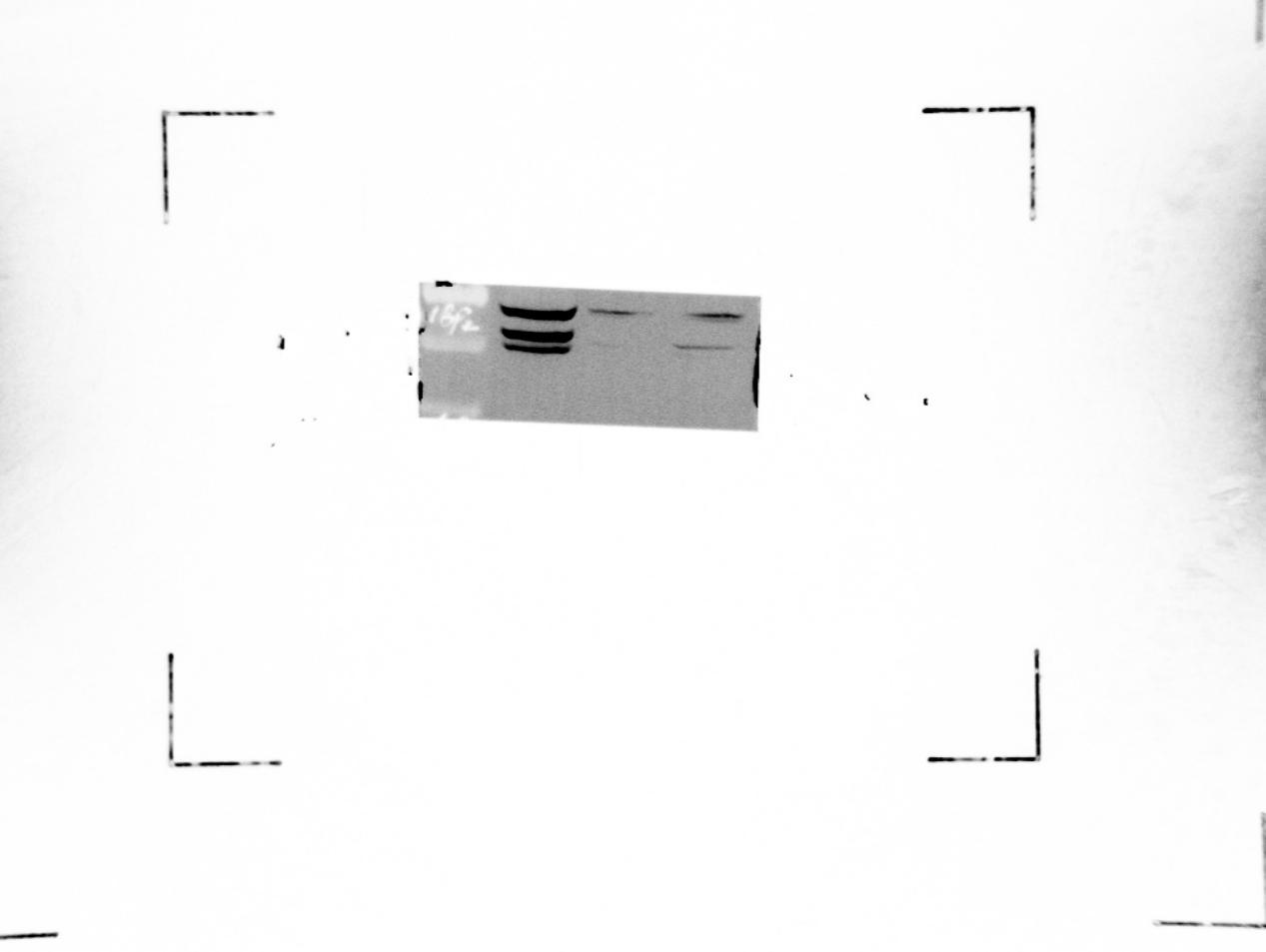

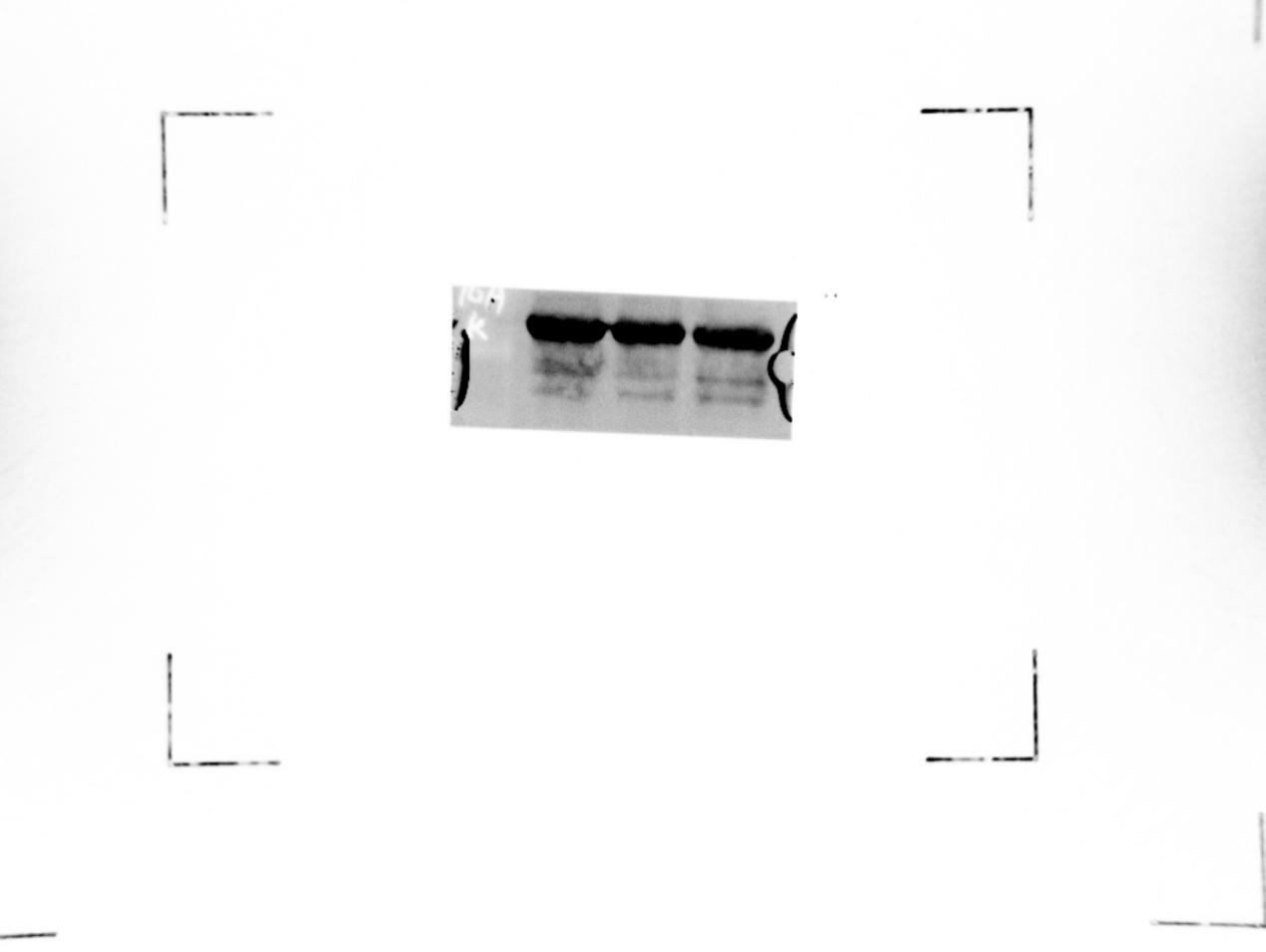


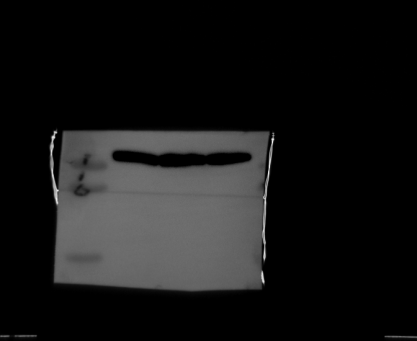

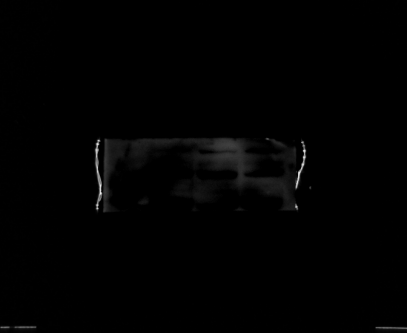

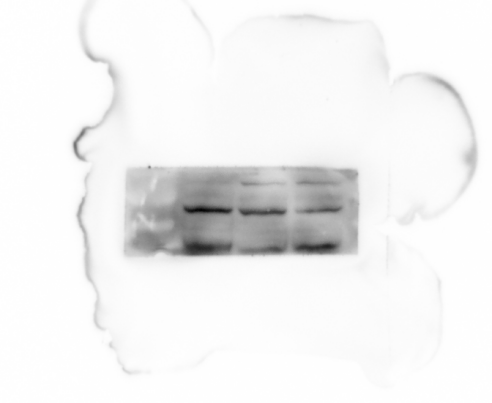


K:


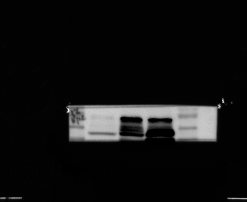

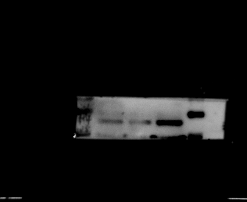

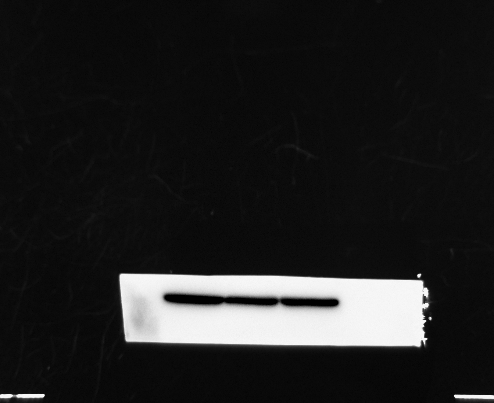

Supplement: Supplementary file 3 — Raw data for western blot of the manuscript [file 41419_2025_7864_MOESM3_ESM.docx]

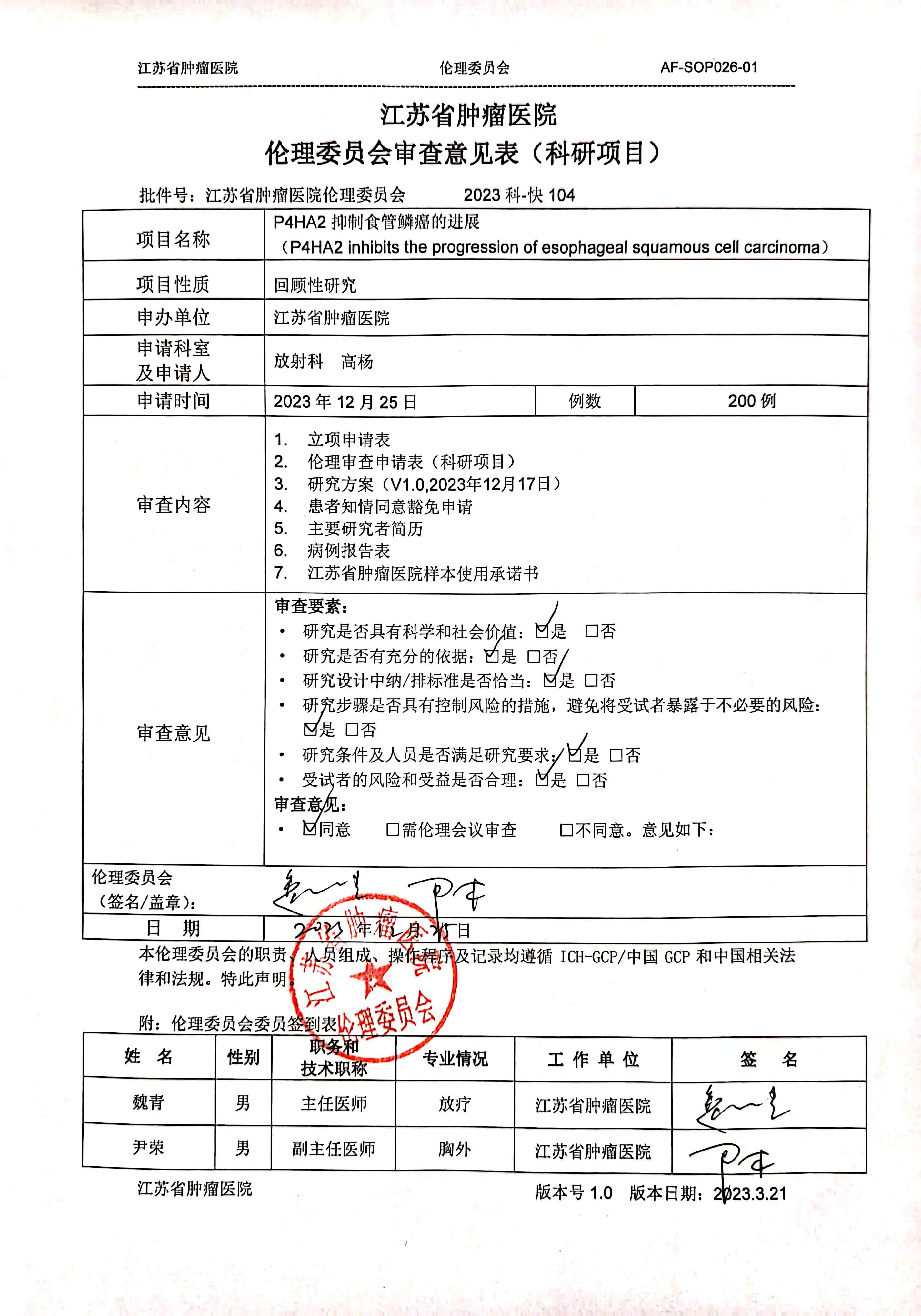

Supplement: Supplementary file 4 — patients ethics [file 41419_2025_7864_MOESM4_ESM.jpg]
