# Supplementary material for: P4HA2 interacted with ATAD3A to modulate PINK1/parkin-dependent mitophagy and 125I brachytherapy sensitization in esophageal carcinoma
Source: Cell Death Dis. 2025 Oct 6;16(1):685. doi: 10.1038/s41419-025-07864-x (PMC12501296; doi:10.1038/s41419-025-07864-x)
Supplement: Supplementary file 5 — animal ethics [file 41419_2025_7864_MOESM5_ESM.pdf]

东南大学动物实验伦理审查表

Animal Experimental Ethical Inspection Form of Southeast University

编号（No）: 20190225004

申请人填写的相关信息 (Related information filled by applicant)

申请单位 Name of organization	东南大学 School of Medicine, Southeast University	经费代码 Funding code	
申请人 Applicant	郭金和 Guo Jinhe	申请人学历 Education of applicant	博士 Doctor
技术职称 Professional title	主任医师 Chief physicians	课题级别 Grade of Project	国家自然基金 NSFC(Natural Science Foundation of China)
实验名称 Experiment title	TGF-β/Smads 信号通路调控 125I 粒子内照射支架治疗食管癌中放射抵抗的机制研究 The Mechanisms of TGF-β/Smads Signaling Pathway Induced Radioresistance during the Brachytherapy of Esophageal Cancer via Irradiation Stent Loaded with 125I Seeds		
质量合格证编号 Number of Quality certification		动物实验设施合格证编号 Number of Experimental Facilities certification	SYXK（苏）2016-0014 SYXK（Su）2016-0014

拟进动物情况

动物来源 Source of animal	实验开始时，由实验动物中心代购 Animals are purchased by Laboratory Animal Center for the applicant during the experiment.				
品种品系 Species of strain	BALB/c 裸鼠 BALB/c nude mouse	等级 Grade	SPF	规格 Specifications	4-6 周龄 4-6 weeks old
数量 Number	♂ 0 只、♀ 120 只，共 120 只	申请日期 Application date	2019 年 2 月 24 日		
进驻日期 Entering date	2022 年 1 月 1 日	结束日期 Ending date	2023 年 12 月 31 日		

实验要点，包括实验目的、实验方法、观测指标、实验结束后处死动物的方法等(Aim of experiment , Outline of experiments, experimental methods, observational index, executing animal method, et al)

为验证干预 TGF-β/Smads 信号通路在提高 ^{125}I 粒子治疗食管癌疗效中的作用，拟构建人食管癌裸鼠皮下移植瘤模型，植入 ^{125}I 粒子，根据实验要求，进行不同手段的干预治疗，记录肿瘤体积变化，并在实验结束时，氟烷麻醉后吸入二氧化碳处死,并收集肿瘤组织和器官。

The objective of this experiment is to investigate the roles of TGF-β/Smads signaling pathway inhibition in brachytherapy of esophageal cancer via 125I seeds. After construction of human esophageal cancer xenograft model, 125I seeds will be implanted. According to the experiment requirement, different interventional and therapeutic methods will be applied and the change of tumor size will be recorded. At the end of the experiment, mice will be executed by CO_2 inhalation after halothane anesthesia, and then tumor tissues and organs will be collected for other tests.

申请人签名 Signature of applicant	(signature)	联系电话 Telephone	办公室(O)	025-83262232
			移　动(M)	13901599698

（请翻看背面）

编号（No）：201902250004

<table>
<tr><td>申请者声明
Announcement of applicant</td><td colspan="4">我将自觉遵守实验动物福利伦理原则，随时接受实验动物伦理委员会的监督与检查，如违反规定，自愿接受处罚。(I will abide by the rules of animal experimental ethics, accept the supervision and inspection of the animal experimental ethics committee, and accept the punishment if any infringement.)

声明人签名(Signature)：
2019 年 2 月 25 日</td></tr>
<tr><td>审查依据
Inspection contents</td><td colspan="4">1. 该项目是否必须用实验动物进行实验，即能否用计算机模拟、细胞培养等非生命方法替代动物或用低等动物替代高等动物进行实验(Does laboratory animal must be used in the project? Could other methods such as computer simulation. cell culture or using the low-grade animal instead of the high－grade animal?)
2. 表中所填申请人资格和所用动物的品种品系、质量等级、规格是否合适，能否通过改良设计方案或用高质量的动物来减少所用动物的数量(Are the qualification of applicant，species or strain，grade and specifications of animals suitable? Could the quantity of animals be reduced by improving the study design or using high quality animals?)
3. 能否通过改进实验方法、调整实验观测指标、改良处死动物的方法，来优化实验方案、善待动物(Could the study design and animal treatment be refined by ameliorating experimental method，adjusting observational index. executing animal method?)</td></tr>
<tr><td rowspan="2">审查结果
Results of inspection</td><td>课题负责人意见
Project director attitude</td><td>同意
Agree ☑</td><td>不同意
Disagree □</td><td>签名
Signature</td></tr>
<tr><td>实验动物伦理委员会意见
Attitude of the Animal Care & Welfare Committee</td><td>同意
Agree ☑</td><td>不同意
Disagree □</td><td>签名
Signature</td></tr>
<tr><td colspan="4">备注(Remark)</td><td>东南大学
实验动物伦理审查章
签章(Stamp)
2019 年 2 月 25 日</td></tr>
</table>

说明：

1. 编号由东南大学实验动物中心分配并填写。
2. 表格所有填写内容请用签字笔填写或电脑打印(签名处除外)。项目负责人、执行人及合作单位负责人均需在声明人签字栏签字。
3. 需随本表递交相关审查资料如实验方案、课题标书等。要求写明项目的意义、必要性、项目中有关实验动物的用途、饲养管理或实验处置方法、预期出现的对动物的伤害、处死动物的方法、项目进行中涉及动物福利和伦理问题的详细描述。
