# Supplementary material for: P4HA2 interacted with ATAD3A to modulate PINK1/parkin-dependent mitophagy and 125I brachytherapy sensitization in esophageal carcinoma
Source: Cell Death Dis. 2025 Oct 6;16(1):685. doi: 10.1038/s41419-025-07864-x (PMC12501296; doi:10.1038/s41419-025-07864-x)
Supplement: Supplementary file 6 — STR for EC109 cell line [file 41419_2025_7864_MOESM6_ESM.pdf]

Report of Human Cell Line Authentication

Ⅰ. Sample

Sample Name: labeled as 'EC109'

Ⅱ. Method and Procedure

1. PCR is amplified with STR Multi-Amplification Kit (PowerPlex 21D System);
2. PCR products are assayed with 3100 DNA Analyzer (Applied Biosystems®).
3. Amplification of gene COX1 and electrophoresis are employed to survey the species of the sample.

Ⅲ. Results

1. The STR profiles of the cell line sample are in the attached table and figure.
2. The search result in ATCC and DSMZ databases.
3. The electrophoresis figure of gene COX1.

EC109: ①No loci has tri-alleles or tetra-alleles. Contamination of other human cell lines are not found (Figure 1 & Table 1). ②Compared the STR data of EC109 cell line in the databases of ATCC and DSMZ, the alleles of EC109 were no more than 80% matched with the alleles of any cells found in both cell banks. ③To the data of an assay (Fang Ye *et al*, 2015*), this cell line named as "EC109". ④ The sample is a human cell line. Contamination of other species cells are not found in the sample (Figure 4).

To all above, the sample is a single cell line, and it is EC109 cell line.

* Ye F, Chen C, Qin J, et al. Genetic profiling reveals an alarming rate of cross-contamination among human cell lines used in China[J]. Faseb Journal Official Publication of the Federation of American Societies for Experimental Biology, 2015, 29(10):4268.

Operator: Xiaohua Mo Auditor: Xuanyi Liang

Guangzhou Cellcook Biotech Co., Ltd

GUANGZHOU CELLCOOK BIOTECH CO.,LTD
11 Apr 2023
CELL STR PROFILING

（Notice:This authentication report is restricted to the cell sold from Guangzhou Cellcook Biotech Co., Ltd, and the date with seal is the date of delivery.）

Figure 1. STR profiles of EC109 cell line

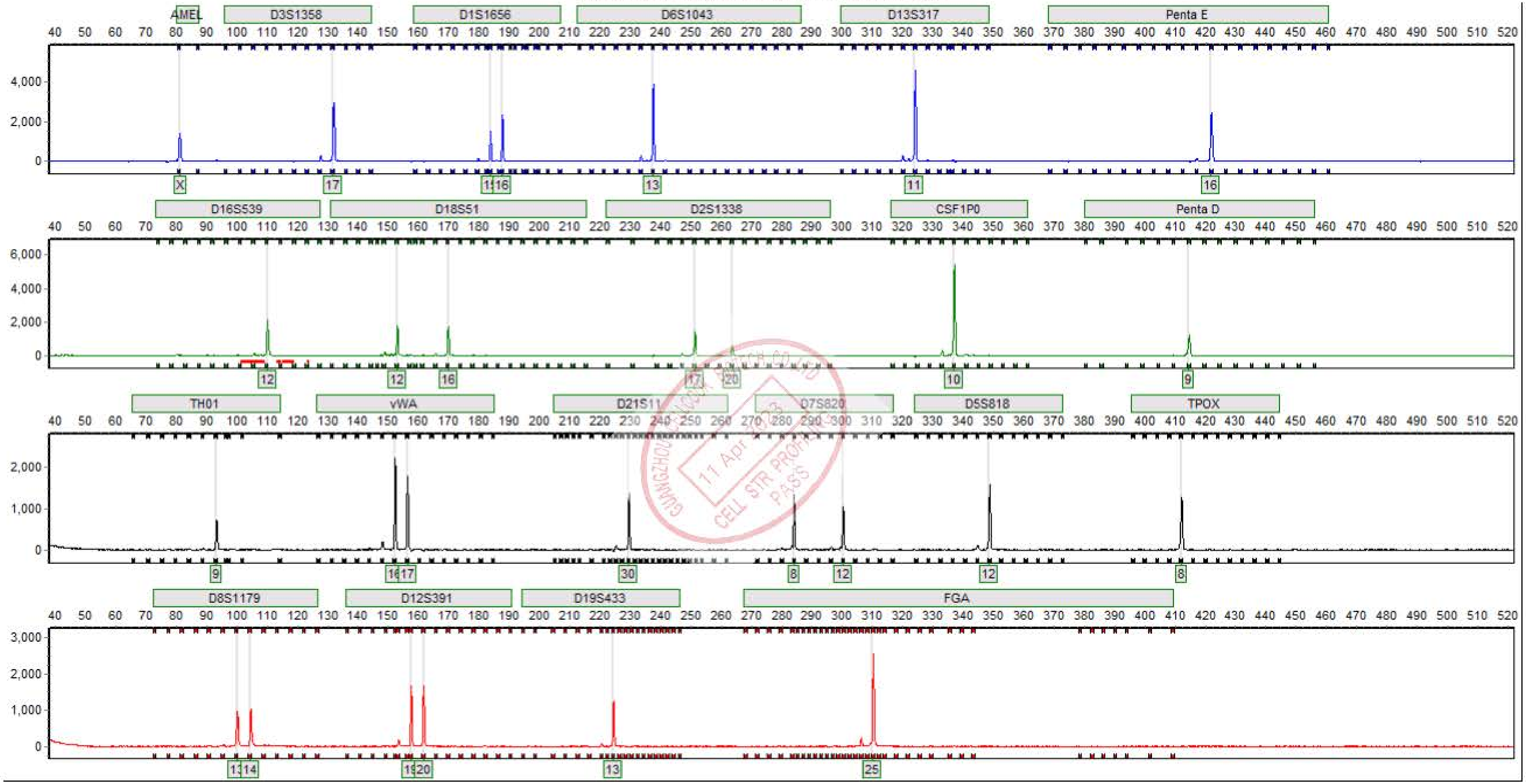

Table 1. STR profiles of EC109 cell line

21	Allele1	Allele2
AMEL	X	
D3S1358	17	
D1S1656	15	16
D6S1043	13	
D13S317	11	
Penta E	16	
D16S539	12	
D18S51	12	16
D2S1338	17	20
CSF1PO	10	
Penta D	9	
TH01	9	
vWA	16	17
D21S11	30	
D7S820	8	12
D5S818	12	
TPOX	8	
D8S1179	13	14
D12S391	19	20
D19S433	13	
FGA	25	

Figure 2. Search result in ATCC database

SEARCH THE STR DATABASE

As part of our continuing efforts to characterize and authenticate the cell lines in the Cell Biology collection, ATCC has developed a comprehensive database of short tandem repeat (STR) DNA profiles for all of our human cell lines. View our brief tutorial before starting.

1. STR Profiling Analysis
2. Matching Algorithm
3. Interrogating the Database

There are no results.

Disclaimer: Reference to this database and the data contained therein may be cited in publications, and ATCC encourages such citation or reference. While every reasonable effort has been made to assure the accuracy of these data, no warranty, express or implied, is made by ATCC as to their accuracy.

Figure 3. Search result in DSMZ database

Result of STR matching analysis by your data.

- DSMZ Profile Database -

A graphical presentation is shown at the bottom of this page.

EV	Cell No.	Cell name	Locus names									Figures
			D5S818	D13S317	D7S820	D16S539	VWA	TH01	AM	TPOX	CSF1PO	
	Query (Your Cell)		*12, 12*	*11, 11*	*8, 12*	*12, 12*	*16, 17*	*9, 9*	*x, x*	*8, 8*	*10, 10*	
0.72(26/36)	196	COLO-699	11, 12	11, 11	11, 13	12, 12	17, 17	9, 9.3	X, X	8, 8	10, 10	-
0.72(26/36)	CRL-4012	NuLi-3	12, 13	11, 11	8, 10	10, 11	16, 17	9, 9	X, X	8, 11	10, 10	-
0.67(24/36)	377	TCC-SUP	12, 12	11, 11	8, 9	9, 11	14, 16	6, 9.3	X, X	8, 8	10, 10	-
0.67(24/36)	502	H-1184	12, 12	11, 11	10, 12	11, 12	16, 17	6, 9.3	X, Y	8, 9	10, 10	-
0.67(24/36)	508	"RS4;11"	12, 12	11, 11	8, 12	11, 12	14, 17	6, 9.3	X, X	8, 8	11, 12	-

Figure 4. Authentication of the species of the sample

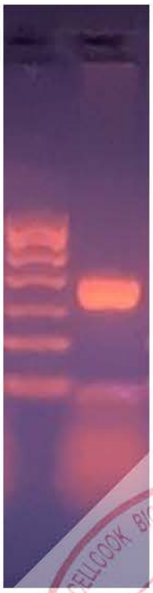

M: Marker. As the size of 700, 600, 500, 400, 300, 200 and 100bp from up to down.

Nine species are checked, as follow: *Homo sapiens* 391bp, *Cricetulus griseus*315bp, *Macaca mulatta*287bp, *Cercopithecus aethiops*222bp, *Rattus norvegicus*196bp, *Canis familiaris*172bp, *Mus musculus*150bp, *Bos Taurus*102bp, IC 70bp

The sample: The band size is 391bp which matches the size of human.
