# Supplementary material for: P4HA2 interacted with ATAD3A to modulate PINK1/parkin-dependent mitophagy and 125I brachytherapy sensitization in esophageal carcinoma
Source: Cell Death Dis. 2025 Oct 6;16(1):685. doi: 10.1038/s41419-025-07864-x (PMC12501296; doi:10.1038/s41419-025-07864-x)
Supplement: Supplementary file 7 — STR for KYSE30 cell line [file 41419_2025_7864_MOESM7_ESM.pdf]

Cell Line Authentication – STR Profiling Report

Sample Type: Cell Line
Testing Type: STR

Sample code:

Table 1. Sample Code

Customer's code	Company Code
942	20210913-03

Sample Number:1

Sample Type: Cell line

Testing Type: STR

Sample From: Shanghai Zhong Qiao Xin Zhou Biotechnology Co.,Ltd.

Testing Method:

DNA was extracted by a commercial kit from CORNING (AP-EMN-BL-GDNA-250G). Twenty short tandem repeat (STR) loci plus the gender determining locus, Amelogenin, were amplified by six multiplex PCR and separated on ABI 3730XL Genetic Analyzer. The signals were then analyzed by the software GeneMapper.

Data Interpretation:

Cell lines were authenticated using Short Tandem Repeat (STR) analysis as described in 2012 in ANSI Standard (ASN-0002) by the ATCC Standards Development Organization (SDO) and in Capes-Davis et al., Match criteria for human cell line authentication: Where do we draw the line? Int J Cancer. 2013;132(11):2510-9.

中乔新舟

Test Results:

1. Result

Table 2. Matching information on the cell lines

Sample Code	Multi-allele	Cell line matched	Cell Bank	EV
20210913-03	YES	KYSE-30	DSMZ	1.0

- Multi-allele means some STR contain more than two loci.

2. Sample Description

20210913-03:

A. The STR results showed that multiple alleles were found in this cell line, and no cross contamination of human cells was found in the cell line.

B. The DNA of the cell lines found to match the type of cell lines in a cell line retrieval, DSMZ database shows that cells called **KYSE-30,** corresponding to the cell number **351.**

3. Genotyping Result

Table 3. STR and Amelogenin Genotyping Results of Cell line 20210913-03

Loci	Sample information			Cell Bank information		
	Sample name：942			Cell line name：KYSE-30		
	**Allele1**	**Allele2**	**Allele3**	**Allele1**	**Allele2**	**Allele3**
D5S818	11	11		11	11	
D13S317	9	9		9	9	
D7S820	11	12		11	12	
D16S539	10	12		10	12	
VWA	16	18	19	16	18	19
TH01	9	9		9	9	

AMEL	X	X	X	X
TPOX	9	9	9	9
CSF1PO	10	10	10	10
D12S391	17	19		
FGA	24	24		
D2S1338	23	23		
D21S11	28	28		
D18S51	14	14		
D8S1179	12	15		
D3S1358	15	16		
D6S1043	11	20		
PENTAE	13	13		
D19S433	14.2	15.2		
PENTAD	12	12		
D1S1656	15	16		

The allele match algorithm compares the 8 core loci plus amelogenin only, even though alleles from all loci will be reported when available.

中乔

Others:

1. Genotyping Strategy and Site Distribution

Attached Table. Experimental Strategy and Sites

	Strategy 1	Strategy 2	Strategy 3	Strategy 4
1	D3S1358	D8S1179	D19S433	AMEL
2	VWA	D21S11	TH01	D1S1656
3	D7S820	D16S539	D13S317	D5S818
4	CSF1PO	D2S1338	TPOX	D12S391
5	PENTAE	PENTAD	D18S51	FGA
6			D6S1043	

2. DSMZ tools was used to carry on the cell line comparison, which contains 2455 cell lines STR data from ATCC, DSMZ, JCRB, ECACC, GNE and RIKEN databases. If the cell is not included in the above cell library, users need to compared with other databases.

中乔新舟

Certificate of STR Analysis

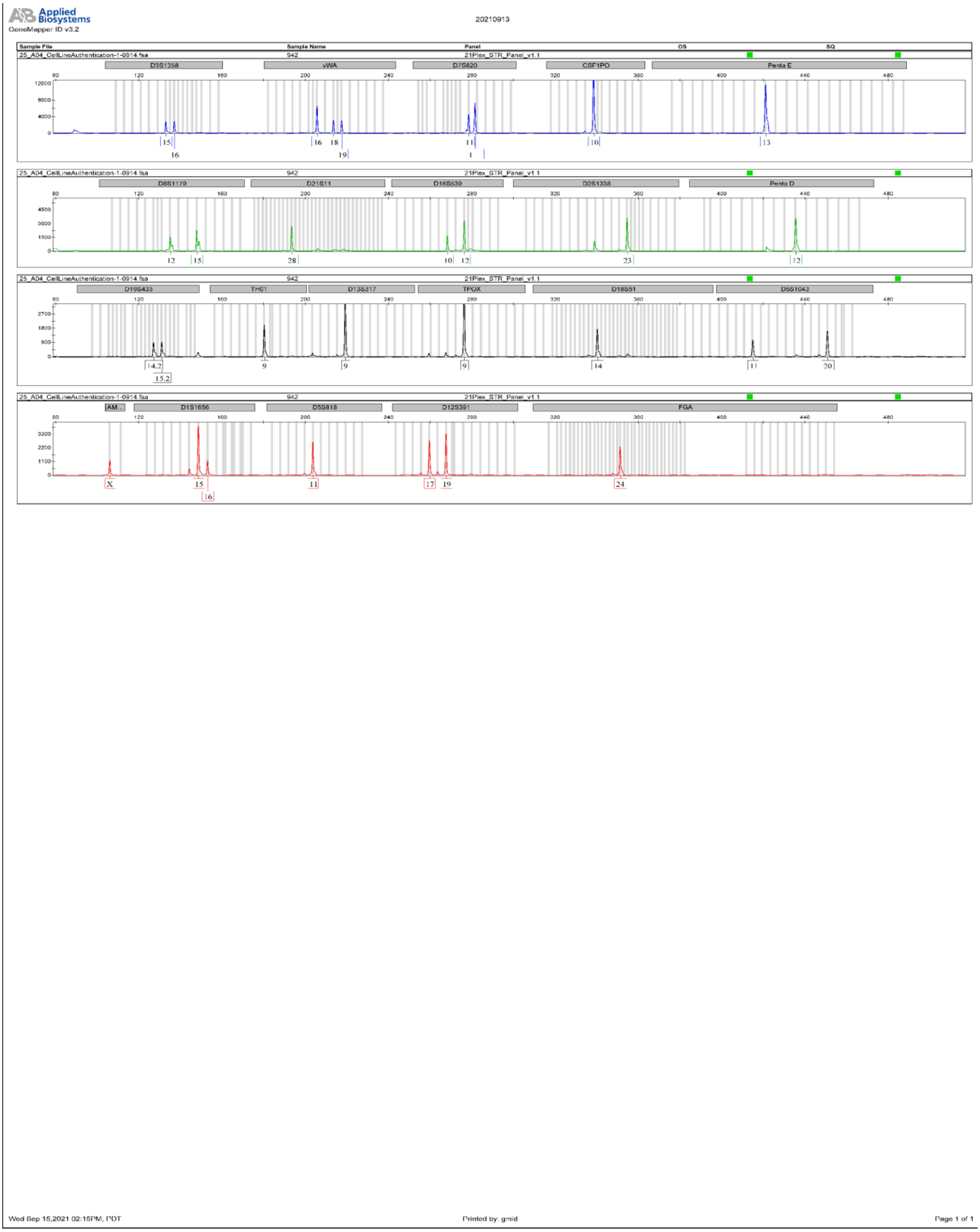

Report Date:
Sep., 2021
