# Supplementary material for: P4HA2 interacted with ATAD3A to modulate PINK1/parkin-dependent mitophagy and 125I brachytherapy sensitization in esophageal carcinoma
Source: Cell Death Dis. 2025 Oct 6;16(1):685. doi: 10.1038/s41419-025-07864-x (PMC12501296; doi:10.1038/s41419-025-07864-x)
Supplement: Supplementary file 8 — STR for KYSE150 cell line [file 41419_2025_7864_MOESM8_ESM.pdf]

Short Tandem Repeat（STR） Report

1. Sample info.

Sample Label： **KYSE-150**

Item No.： **TCH-C236**

2. Data Interpretation

1) Human Cell Line Authentication Standardization of Short Tandem Repeat (STR) Profiling ASN-0002 Revised version 2021
2) The Pharmacopoeia of the People's Republic of China, Volume III General Requirements, Requirements for Preparation and Control of Animal Cell Substrates Used for Production of Biologics

3. Methodology

Sample DNA was extracted with genome extraction kit (Axygen) and PCR amplified with STR Multi-amplification Kit (MicroreaderTM21 ID System). PCR products were assayed with ABI 3730xl DNA Analyzer (Applied Biosystems®). Appropriate positive and negative controls were run and confirmed for each sample submitted. Data of 20 STR loci plus the Amelogenin locus were analyzed and extracted using Gene Mapper ID-X software and designated as the STR profile of the test sample.

4. Identify Result

The results of the negative and positive control match expectations.

The STR profiles of the test sample are clear, which are shown in the attached table and figure.

Test results for Multiple Alleles[3]: Number of multiple alleles: 0, Locus: None.

5. Explanation of Test Results

1) The results of cellular STR typing of the sent cell DNA showed that no cross contamination of human cells was found in this cell line.
2) The cell DNA typing of this strain found a cell line matching its cell typing in the cell line search, and the DSMZ database showed a cell name of KYSE-150 with a data match rate EV of 1.0.
3) No multiple alleles were found in this cell line in this assay.

Note: This report is only responsible for the samples submitted for this inspection.
Conclusion: According to ATCC ASN-0002-2021，Cell lines with ≥80% match at 13 STR loci or ≥90% match at eight STR loci are considered to be related.

QC Review By Kathy Fang
Haixing Biosciences.

为科研加速，为工业赋能！
海星商城二维码 公众号二维码
CRISPR/Cas9细胞基因编辑
载体构建/病毒包装 分子诊断标准品/突变基因标准品/融合基因标准品
稳转细胞株 HyCyte®干细胞/原代细胞/细胞系产品 HyCyte®细胞培养试剂

Results of database comparison (the highest percent match to the query):

	DSMZ		Sample	
Cell Name	KYSE-150		KYSE-150	
Cell ID	Allele		——	
D19S433	15	15.2		
D5S818	12	13	12	13
D21S11	30	31		
D18S51	14	14		
D6S1043	18	20		
AMEL	X	X	X	X
D3S1358	15	16		
D13S317	8	11	8	11
D7S820	10	11	10	11
D16S539	9	11	9	11
CSF1PO	12	13	12	13
PentaD	10	10		
D2S441	10	11		
vWA	16	17	16	17
D8S1179	10	15		
TPOX	8	8	8	8
PentaE	12	18		
TH01	7	9	7	9
D12S391	19	22		
D2S1338	25	25		
FGA	21	24		
Matching[5]	100%			

Interpretation[6] Likely to be from the same donor.

Remarks

为科研加速，为工业赋能！
海星商城二维码 公众号二维码
CRISPR/Cas9细胞基因编辑
载体构建/病毒包装 分子诊断标准品/突变基因标准品/融合基因标准品
稳转细胞株 HyCyte®干细胞/原代细胞/细胞系产品 HyCyte®细胞培养试剂

1. The short tandem repeat (STR) profile is indicative only of the sample sent to Suzhou Haixing Co., Ltd at the time it was sent. Effective peaks are real PCR bands, small peaks and non-specific bands are ignored in calculation.

2. The STR genotyping profile of the submitted cell is compared with ATCC, DSMZ and ExPASy databases, in which STR genotypes of over 100,000 human cell lines from ATCC, DSMZ, JCRB, ECACC, Riken etc. were recorded. Cells that are not included in above databases could not be compared. For failed sample, it's genotyping and figure result would be shown as NA.

3. Both mixed cultures and MSI positive cell lines can show additional STR alleles in one or more STR loci. In short, two fundamentally different causes can be the reason for multiple alleles at a locus: drifted alleles by MSI or some other DNA replication error or the presence of a mixed culture containing a contaminating second human cell line.

4. The number of STR loci shared by the submitted sample and the cells in database. Amelogenin does not belong to STR locus.

5. The matching score of DSMZ and ExPASyis "Tanabe"— 2 x (number of alleles matching) / (total number of alleles in database profile and query sample） x 100%. The matching score of ATCC is (the number of shared alleles between query sample and database profile) ∕ （total number of alleles in database profile） x 100%.

6. The interpretation is based on "ATCC ASN-0002-2021"."Two samples are considered possibly related when their STR profiles match at 80-90% of alleles across the 13 core STR loci, as calculated using the matching algorithm. At matches of 90% or greater, the cell lines have very likely originated from the same donor. Samples between 60 and 79% match tend to be unrelated, but need may further investigation to confirm that they are unrelated. Two common causes of related cell lines scoring below 80% are (1) if the cell line has extra alleles due to it being MSI unstable, and (2) misidentification of the cell line's origin. Generally, cell lines are authentic (i.e., are derived from the same original tissue donor) when they show the expected percent match (≥ 80% at 13 STR loci or > 90% match at eight STR loci) to other results from that donor (tissue or other cell line samples within the database)."

7. The testing results were calculated and generated by each cell database ONLINE tool. In some case, there may be different profiles of same cell line in each database and possibly lead to different matching results. In addition, limited by the algorithm of the calculation model (the less the number of shared STR sites, the higher the probability of matching unrelated cells), non-cell line(s)human cells/sample(s) may also match to cell(s) in database. In these conditions, the comparison results in this report are for reference only.

8. The results are judged according to industry standards or consensus and can only be used for cell line inspection and authentication. We are NOT responsible for any purpose or interpretation other than the purpose specified.

为科研加速，为工业赋能！
海星商城二维码
公众号二维码
CRISPR/Cas9细胞基因编辑
载体构建/病毒包装　分子诊断标准品/突变基因标准品/融合基因标准品
稳转细胞株　HyCyte®干细胞/原代细胞/细胞系产品　HyCyte®细胞培养试剂

Figure1: Genotyping of STR and Amelogenin loci in cells

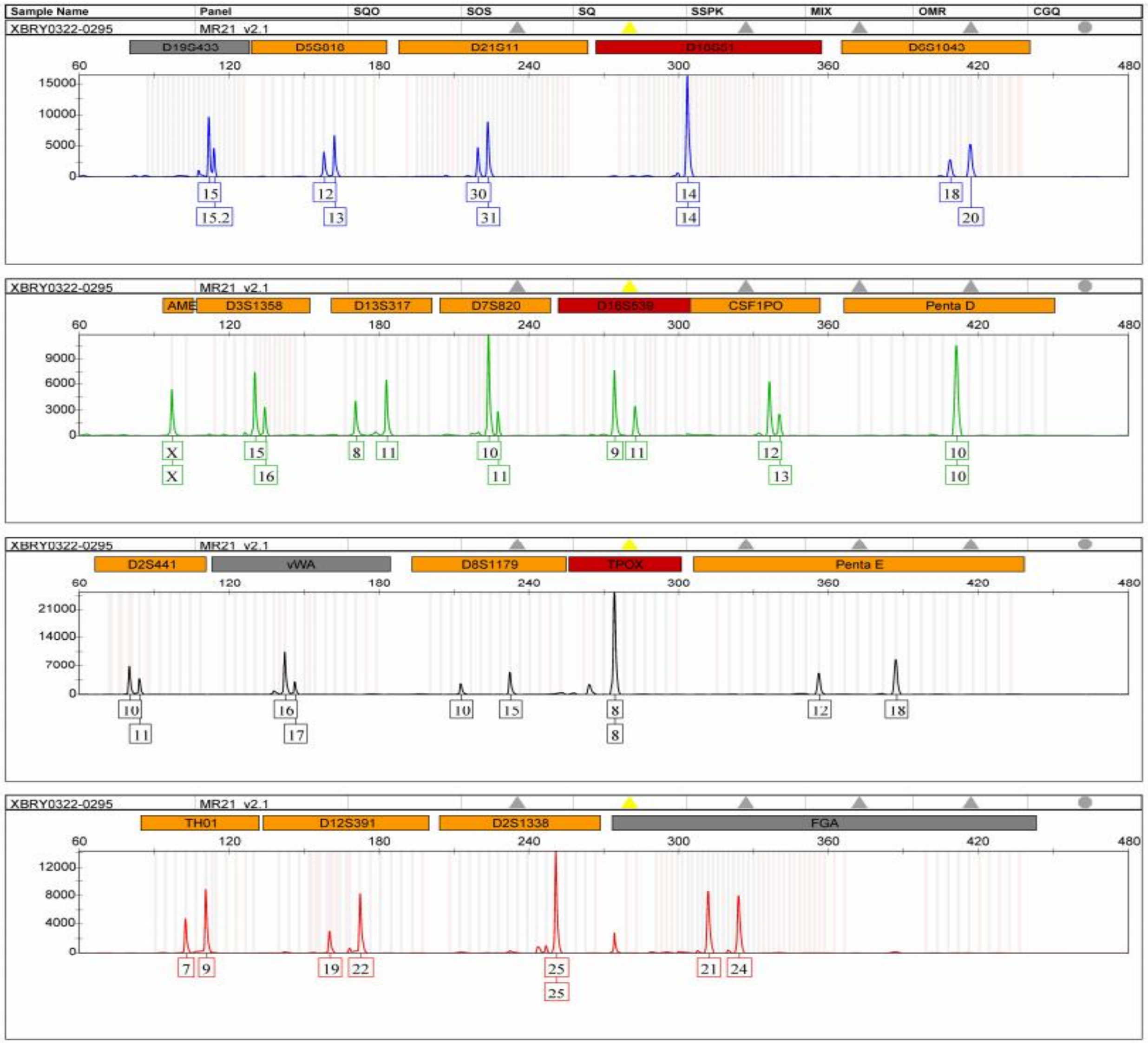

为科研加速，为工业赋能!
关注海星公众号
关注海星视频号
CRISPR/Cas9细胞基因编辑
载体构建/病毒包装 分子诊断标准品/突变基因标准品/融合基因标准品
稳转细胞株 HyCyte®干细胞/原代细胞/细胞系产品 HyCyte®细胞培养试剂

Figure2: Result of STR matching analysis in DSMZ STR database

STR Profile Search

Help

The human STR profile database includes data sets of 2455 cell lines from ATCC, DSMZ, JCRB and RIKEN.

Refine search | Start new search

Similarity	Cell line	Source	D5S818			D13S317		D7S820		D16S539		vWA			TH01			TPOX		C[illegible]
?	Your query		12	13		8	11	10	11	9	11	16	17		7	9		8	8	12
100 %	KYSE-150	DSMZ: ACC-375	12	13		8	11	10	11	9	11	16	17		7	9		8	8	12
72.2 %	JHONA	RCB2365	12	13		**9**	11	10	**10**	9	11	**15**	17		**6**	9		8	8	12
70.3 %	SF8404	RCB0582	**10**	**12**		8	11	**11**	11	9	11	16	17		**6**	9	**9.3**	8	**11**	12
68.4 %	SF8543	RCB0608	**10**	**11**	**12**	8	11	**11**	**12**	9	11	16	17	**19**	7	9		8	8	**11**
66.7 %	HCEC-H9C1	DSMZ: ACC-649	**11**	**11**		8	11	10	11	**11**	11	**14**	17		**9**	9		8	8	12
66.7 %	SKNO-1	DSMZ: ACC-690	**10**	13		8	11	10	**12**	**10**	11	**14**	17		7	**7**		8	**9**	12
66.7 %	HCC-1599BL	DSMZ: ACC-894	12	**12**		8	11	10	11	**12**	**13**	**17**	17		**9.3**	**9.3**		8	8	12
66.7 %	SK-N-DZ	CRL-2149	12	**12**		8	11	**12**	**13**	9	11	16	**18**		**6**	9		8	8	12
66.7 %	HCC1599 BL	CRL-2332	12	**12**		8	11	10	11	**12**	**13**	**17**	17		**9.3**	**9.3**		8	8	12
66.7 %	Hs 755 (B) .T	CRL-7489	12	**12**		8	11	**11**	11	9	11	16	17		7	**8**		8	**11**	**10**

为科研加速，为工业赋能！

关注海星公众号　关注海星视频号

CRISPR/Cas9细胞基因编辑
载体构建/病毒包装　分子诊断标准品/突变基因标准品/融合基因标准品
稳转细胞株　HyCyte®干细胞/原代细胞/细胞系产品　HyCyte®细胞培养试剂
