# Supplementary material for: P4HA2 interacted with ATAD3A to modulate PINK1/parkin-dependent mitophagy and 125I brachytherapy sensitization in esophageal carcinoma
Source: Cell Death Dis. 2025 Oct 6;16(1):685. doi: 10.1038/s41419-025-07864-x (PMC12501296; doi:10.1038/s41419-025-07864-x)
Supplement: Supplementary file 9 — STR for TE-1 cell line [file 41419_2025_7864_MOESM9_ESM.pdf]

Report of Human Cell Line Authentication

（Notice:This authentication report is restricted to the cell sold from Guangzhou Cellcook Biotech Co., Ltd, and the date with seal is the date of delivery. ）

Ⅰ. Sample

Sample Name: labeled as ‘TE-1’

Ⅱ. Method and Procedure

1. PCR is amplified with STR Multi-amplification Kit (PowerPlex™16HS System);
2. PCR products are assayed with 3100 DNA Analyzer (Applied Biosystems®).
3. Amplification of gene COX1 and electrophoresis are employed to survey the species of the sample.

III. Results

1. The STR profiles of the cell line sample are in the attached table and figure.
2. The search result in ATCC and DSMZ databases.
3. The electrophoresis figure of gene COX1.

TE-1: ①No loci has tri-alleles or tetra-alleles. Contamination of other human cell line is not found (Figure 1 & Table 1). ②100% matched cell lines are found in DSMZ data bank. And the cell line named as “TE-1” et *al.* 100% matched cell lines are not found in ATCC data bank (Figure 2 & Figure 3).. ③The sample is a human cell line. Contamination of other species cells are not found in the sample (Figure 4).

GUANGZHOU CELLCOOK BIOTECH CO.,LTD
19 NOV 2018
CELL STR PROFILING
PASS

Operator: Xiaohua Mo Auditor: Xuanyi Liang

Guangzhou Cellcook Biotech Co., Ltd

Figure 1. STR profiles of TE-1 cell line

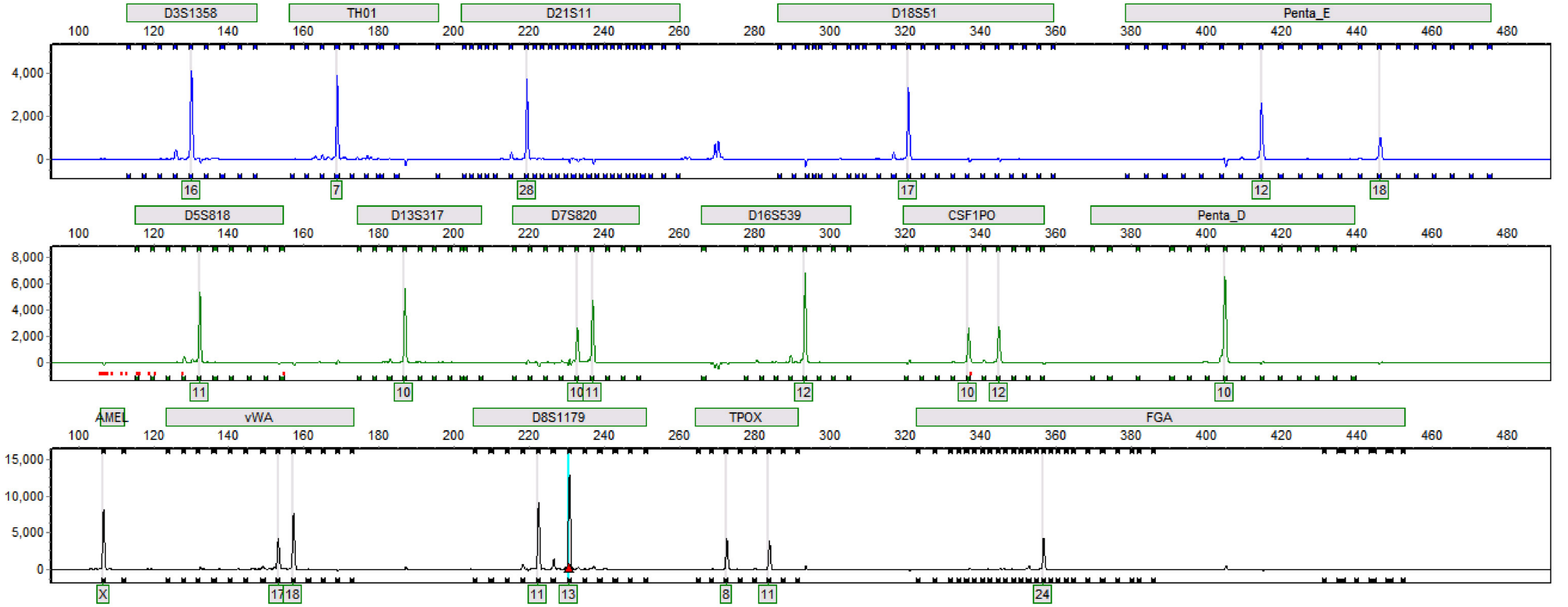

Table 1. STR profiles of TE-1 cell line

	Allele1	Allele2
D3S1358	16	
TH01	7	
D21S11	28	
D18S51	17	
Penta_E	12	18
D5S818	11	
D13S317	10	
D7S820	10	11
D16S539	12	
CSF1PO	10	12
Penta_D	10	
AMEL	x	
vWA	17	18
D8S1179	11	13
TPOX	8	11
FGA	24	

Figure 2. Search result in ATCC database

SEARCH THE STR DATABASE

As part of our continuing efforts to characterize and authenticate the cell lines in the Cell Biology collection, ATCC has developed a comprehensive database of short tandem repeat (STR) DNA profiles for all of our human cell lines. View our brief tutorial before starting.

1. STR Profiling Analysis
2. Matching Algorithm
3. Interrogating the Database

There are no results.

Figure 3. Search result in DSMZ database

Result of STR matching analysis by your data.

- DSMZ Profile Database -

A graphical presentation is shown at the bottom of this page.

EV	Cell No.	Cell name	Locus names									Figures
			D5S818	D13S317	D7S820	D16S539	VWA	TH01	AM	TPOX	CSF1PO	
	Query (Your Cell)		*11*	*10*	*10,11*	*12*	*17,18*	*7*	*x*	*8,11*	*10,12*	
1.16(36/31)	RCB1894	TE-1	11,11	10,10	10,11	12,12	17,18	7,7	X,X	8,11	10,12	-
0.97(30/31)	CRL-5863	NCI-H1334	11,11	12,12	11,11	11,12	17,17	7,7	X,X	8,8	12,12	-
0.90(28/31)	706	OE-33	11,11	14,14	9,10	12,12	17,17	7,7	X,X	8,11	10,11	-
0.90(28/31)	CRL-5810	NCI-H522 [H522]	11,11	10,10	9,10	12,12	17,17	7,9.3	X,X	9,10	10,10	-
0.90(28/31)	CRL-5889	NCI-H1703 [H1703]	11,11	10,10	10,12	10,12	16,17	7,7	X,Y	8,11	12,12	-
0.90(28/31)	CRL-5908	NCI-H1975 [H-1975, H1975]	11,12	10,13	8,11	9,12	18,18	7,7	X,X	8,11	12,12	-
0.90(28/31)	CRL-5976	NCI-H64 [H64]	11,11	10,10	10,12	9,12	17,18	6,6	X,X	8,8	10,10	-
0.90(28/31)	JCRB1153	HCA	11,11	12,12	10,11	11,11	18,18	7,7	X,X	8,11	12,12	-
0.90(28/31)	CRL-5806	NCI-H322	11,11	12,12	11,11	11,12	17,17	7,7	X,Y	8,8	12,12	-

Figure 4. Authentication of the species of the sample

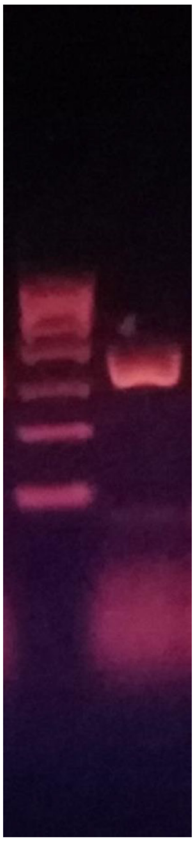

M: Marker. As the size of 700, 600, 500, 400, 300, 200 and 100bp from up to down.

Nine species are checked, as follow: *Homo sapiens* 391bp, *Cricetulus griseus*315bp, *Macaca mulatta*287bp, *Cercopithecus aethiops*222bp, *Rattus norvegicus*196bp, *Canis familiaris*172bp, *Mus musculus*150bp, *Bos Taurus*102bp, IC 70bp

The sample: The band size is 391bp which matches the size of human.
